# In Vitro Efficacy of Bacterial Cellulose Dressings Chemisorbed with Antiseptics against Biofilm Formed by Pathogens Isolated from Chronic Wounds

**DOI:** 10.3390/ijms22083996

**Published:** 2021-04-13

**Authors:** Karolina Dydak, Adam Junka, Agata Dydak, Malwina Brożyna, Justyna Paleczny, Karol Fijalkowski, Grzegorz Kubielas, Olga Aniołek, Marzenna Bartoszewicz

**Affiliations:** 1 Department of Pharmaceutical Microbiology and Parasitology, Medical University of Wroclaw, 50-556 Wroclaw, Poland; karolina.dydak@umed.wroc.pl (K.D.); malwina.brozyna@student.umed.wroc.pl (M.B.); justyna.paleczny@student.umed.wroc.pl (J.P.); marzenna.bartoszewicz@umed.wroc.pl (M.B.); 2 Faculty of Biological Sciences, University of Wroclaw, 51-148 Wroclaw, Poland; agata.dydak@op.pl; 3 Department of Microbiology and Biotechnology, Faculty of Biotechnology and Animal Husbandry, West Pomeranian University of Technology, Szczecin, Piastow 45, 70-311 Szczecin, Poland; kfijalkowski@zut.edu.pl; 4 Faculty of Health Sciences, Wroclaw Medical University, 50-996 Wroclaw, Poland; grzegorz.kubielas@umed.wroc.pl; 5 Faculty of Medicine, Lazarski University, 02-662 Warsaw, Poland; olga.aniolek@lazarski.pl

**Keywords:** bacterial cellulose, dressing, antiseptics, chronic wounds

## Abstract

Local administration of antiseptics is required to prevent and fight against biofilm-based infections of chronic wounds. One of the methods used for delivering antiseptics to infected wounds is the application of dressings chemisorbed with antimicrobials. Dressings made of bacterial cellulose (BC) display several features, making them suitable for such a purpose. This work aimed to compare the activity of commonly used antiseptic molecules: octenidine, polyhexanide, povidone-iodine, chlorhexidine, ethacridine lactate, and hypochlorous solutions and to evaluate their usefulness as active substances of BC dressings against 48 bacterial strains (8 species) and 6 yeast strains (1 species). A silver dressing was applied as a control material of proven antimicrobial activity. The methodology applied included the assessment of minimal inhibitory concentrations (MIC) and minimal biofilm eradication concentration (MBEC), the modified disc-diffusion method, and the modified antibiofilm dressing activity measurement (A.D.A.M.) method. While in 96-well plate-based methods (MIC and MBEC assessment), the highest antimicrobial activity was recorded for chlorhexidine, in the modified disc-diffusion method and in the modified A.D.A.M test, povidone-iodine performed the best. In an in vitro setting simulating chronic wound conditions, BC dressings chemisorbed with polyhexanide, octenidine, or povidone-iodine displayed a similar or even higher antibiofilm activity than the control dressing containing silver molecules. If translated into clinical conditions, the obtained results suggest high applicability of BC dressings chemisorbed with antiseptics to eradicate biofilm from chronic wounds.

## 1. Introduction

### 1.1. Non-Healing Wound Infections—A Current Medical Problem

The disease entity referred to as chronic wound is predominantly a result of patient comorbidities such as diabetes, obesity, and disorders of the immune and/or cardiovascular system. Long-term effects of these diseases translate into disturbances of chronic wound healing. The open cavity of a chronic wound, often filled with an abundant volume of exudate (wound fluid), which is rich in nutrients, constitutes a perfect environment for microbial development [1,2,3,4]. The presence of microorganisms in open wounds is a natural phenomenon and does not always delay the healing process [5,6]. However, along with microbial multiplication in the wound, the capacity of the immune system to control it declines together with the wound condition. This phase is referred to as “critical colonization”, and its symptoms are often difficult to grasp before an obvious burst of local infection. Critical colonization is a phase when local antisepsis should be included in the therapeutic algorithm. The established scales, such as wound at-risk score (WAR) or therapeutic index for local infections score (TILI), are helpful in capturing critical colonization [7,8,9,10,11].

**Figure 1:**
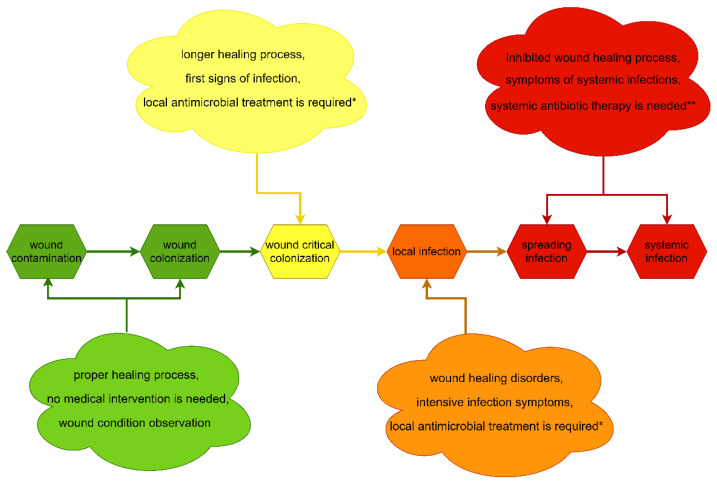
Stages of wound infection and corresponding clinical symptoms [9,10,12]. * antisepsis, wound debridement, antimicrobial dressings, non-antibiotic local treatment. Local antibiotic therapy is not recommended. ** systemic empiric antibiotic therapy depends on the clinical condition of the patient. Empirical treatment should consider the expected pathogens, the site and nature of the infection, and cover the broadest possible spectrum of microorganisms. Empirical antibiotic therapy should be turned into targeted antibiotic therapy as soon as possible after obtaining microbiology test results.

Wound infections are caused by biofilms–complex structures consisting of a large bacterial/yeast cell population communicating with each other and producing a heterogeneous, protective, extracellular matrix. Biofilm formation within the wound causes therapeutic issues, mostly due to the high tolerance of this structure to physical and chemical eradicative agents. A biofilm-based wound infection can also develop into a life-threatening, systemic infection [2,3,4]. The process of development of a biofilm-based infection in a wound and the corresponding clinical symptoms are presented in Figure 1.

To counteract biofilm-based wound infections, the European Wound Management Association (EWMA) introduced and recommended the TIME strategy, which in 2019 was extended to TIMERS. The four main pillars of the TIME strategy were: T—tissue debridement, I—infection and inflammation control, M—moisture balance, E—edges, epithelization stimulation, and the two new ones added in the TIMERS strategy are R—Repair of tissue and regeneration and S—Social factors that impact healing [13,14,15,16].

Considering the topic at hand, the “I” component is the crucial one. Antiseptics need to be applied to prevent/fight against wound infection [12,17]. The most ubiquitous and commonly used antiseptic products are octenidine dihydrochloride (OCT), polyhexamethylene biguanide hydrochloride (polyhexanide, PHMB), iodine povidone (PVP-I), chlorhexidine (CHX) and ethacridine lactate (EL), although the latter is not recommended by any acknowledged wound care organization. Recently, a new class of antiseptic agents (or rather a new formula of already-known type of antiseptics) has been introduced and become widespread, namely super-oxidized hypochlorite solutions (SOH) [1,2,18].

### 1.2. Polyhexamethylene Biguanide Hydrochloride (Polyhexanide, PHMB)

PHMB selectively affects the cytoplasm and the cytoplasmic cell membrane and causes microbial DNA damage [19,20]. PHMB is active against Gram-positive (including methicillin-resistant *Staphylococcus aureus* (MRSA) and vancomycin-resistant *Enterococcus* (VRE)) strains and Gram-negative bacteria, in both planktonic and biofilm forms, spore-forming bacteria, intracellular bacteria, yeast, and the human immunodeficiency virus (HIV) [19,21]. The anti-biofilm activity of PHMB was evaluated in numerous in vitro and in vivo studies (including clinical ones). The results show that PHMB substantially removes biofilm and can prevent biofilm formation [22,23,24,25,26,27,28]. PHMB is reported as an effective antibacterial agent with prolonged action (minimum 2–3 h after exposure) and a low potential to induce microbial resistance [17,20,29]. Studies have shown that PHMB is well-tolerated when administered topically on the skin, mucosa, or the wound has low toxicity to eukaryotic cells and rarely causes allergies [17,19,30]. The wound’s higher pH stimulates the antimicrobial and anti-biofilm activity of PHMB. Moreover, PHMB improves the process of granulation and wound healing [17,31,32].

### 1.3. Octenidine Dihydrochloride (OCT)

The action of OCT is based on its interaction with the cell wall and membrane structures, leading to enzymatic systems damage, cellular function disorders, cytoplasm leakage and, consequently, cell death. Pathogen cell damage causes chemotaxis of granulocytes, which, in turn, reduces the duration of the infection [17,33]. OTC has a broad spectrum of action, including Gram-positive and Gram-negative bacteria, fungi, viruses and protozoa, MRSA and multi-drug resistant (MDR) strains [33,34,35]. Also, the anti-biofilm activity of OCT was proven in in vitro and in vivo studies. The results showed that OCT not only effectively eradicates fully-grown biofilm but also prevents biofilm from forming [26,27,36,37,38]. Similar to PHMB, OCT has high antimicrobial effectiveness and prolonged action time [17,39]. Until recently, OCT has been considered as not inducing resistance. However, in 2018 there was observed a reduced susceptibility to OCT in *Pseudomonas* and *Burkholderia* strains [40,41]. OCT has low cytotoxicity to eukaryotic cells and is biocompatible. Allergies are reported rarely [17,30,42].

### 1.4. Chlorhexidine (CHX)

CHX binds to the microbial cytoplasmic membrane and leads to its disruption. A low concentration of CHX (causing destruction of cell membrane components and dehydrogenase stimulation) is bacteriostatic, while high concentrations of CHX are bactericidal. In such a high concentration, CHX inhibits enzymes and coagulates proteins and cytoplasm components. Higher wound pH enhances CHX activity [43]. CHX has a broad spectrum of action, which includes Gram-positive and Gram-negative bacteria, fungi, enveloped viruses, and protozoa [44,45,46]. Studies have proved that CHX has a bactericidal effect against biofilm, but it does not effectively remove biofilm from surfaces [45,47,48]. Anti-biofilm effectiveness of CHX against dental plaque was also proved [49,50,51]. CHX applied to the skin or to an abiotic surface has prolonged activity [44,45,46]. Noteworthy, CHX induces bacterial resistance and leads to cross-resistance to antibiotics. Despite that, CHX is still widely used in wound treatment all over the world [17,52]. Unfortunately, there are numerous side effects related to its application, such as cytotoxicity, anaphylactic reaction, and risk of hydrolysis to cancerogenic 2-chloroaniline [42,53].

### 1.5. Povidone-Iodine (PVP-I)

PVP-I irreversibly binds to proteins, lipids and nucleic acids of bacterial cells and induces pores in their cell walls. Changes in phospholipids’ unsaturated fatty acids structure lead to cell membrane damage. Because of protein oxidation, microbial enzymes are inactivated; moreover, the iodization of the derivatives of pyrimidine bases and amino acids leads to DNA structure damage [54,55,56]. PVP-I is active against Gram-positive, and Gram-negative bacteria, fungi, viruses, protozoa, bacterial spores and can also inactivate bacterial toxins [17,55,56]. Strong anti-biofilm activity of PVP-I has been reported in in vitro and in vivo studies [55,57,58,59,60,61,62,63,64]. PVP-I does not have a prolonged mode of action [17]. There is no evidence of induction of bacterial resistance resulting from PVP-I use [55,56,65]. PVP-I cytotoxicity is low, but there is a risk of penetration of free iodine into the blood. Therefore, PVP-I should not be used longer than seven days, and it is not recommended for patients with thyroid problems. Povidone-iodine has a brown color that fades with the loss of povidone antimicrobial activity [17,29,55].

### 1.6. Ethacridine Lactate (EL)

The action of EL involves inhibition of bacterial nucleic acid synthesis. EL acts against vegetative forms of Gram-positive and Gram-negative bacteria and fungi [66]. The anti-biofilm activity of EL is very slight, especially against the biofilm formed by Gram-negative rods [10,35,67]. There is no evidence of EL’s prolonged time of action. Reports show that the nosocomial pathogen *Pseudomonas aeruginosa* can survive and multiply in EL solutions [68,69]. Studies have shown that EL has average antimicrobial activity compared to other antiseptics and may delay the healing process, causes allergies as well as cytotoxic, genotoxic, and mutagenic effects. [35,70]. EL is presently considered an obsolete antimicrobial compound, and its usefulness is questionable due to many side effects. However, EL is still in use in hospitals and for outpatients because of its low price, easy access, and force of habit [17].

### 1.7. Super-Oxidized Hypochlorite Solutions (SOH)

Hypochlorites have been used as antimicrobial agents for a long time. Modern super-oxidized solutions of hypochlorites include a combination of NaOCl and HOCl, and a have stable formula [17]. After SOH application, reactive oxygen species are formed. HOCl denatures and increases the permeability of the microbial cell wall, which leads to the inflow of water into the cell and to its destruction as a result of osmotic pressure [17,71]. The purported spectrum of SOH activity includes Gram-positive and Gram-negative bacteria, MRSA and VRE strains, fungi, and viruses [17,71,72,73]. Noteworthy, there are some inconsistencies in research results regarding SOH’s antimicrobial and anti-biofilm activity. In some studies, strong efficacy of SOH against biofilm was shown [74,75,76,77,78], whereas other studies described SOH as poorly working or not working at all. These discrepancies can be due to using various methods and different concentrations of NaOCl and HOCl. While the antimicrobial activity of high chlorine concentrations is undisputable, the extrapolation of favorable results to all SOH-containing products (regardless of the chlorine content) seems to be inappropriate [27,79,80,81,82,83]. There is a lack of evidence for a prolonged time of action of SOH. In low concentrations, SOH is considered safe in use (towards eukaryotic cells) and not causing cytotoxic effects. The use of SOH can support autolytic wound cleansing processes and can have anti-inflammatory effects [17,71,80].

### 1.8. Modern Multifunctional Dressings for Non-Healing Wounds

**Figure 2:**
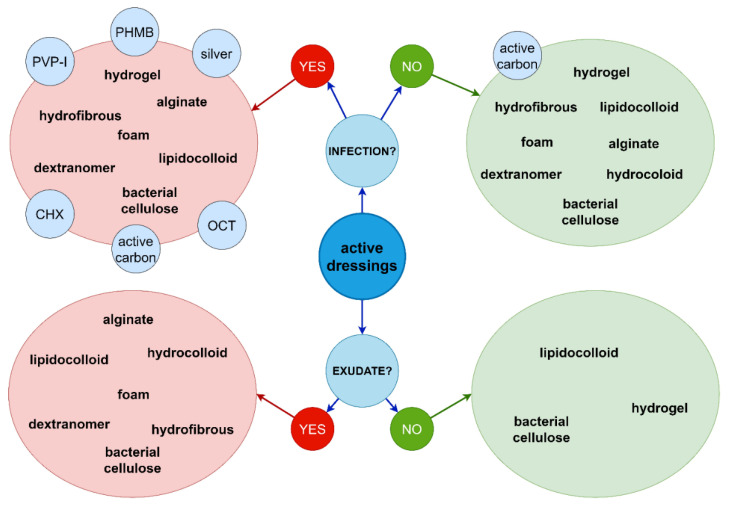
Examples of commercially available modern active dressings and their applications [84,86,87,88,89,90,91,92,93,94,95,96]. PHMB—polyhexanide; PVP-I—povidone-iodine; CHX—chlorhexidine; OCT—octenidine.

The novel active dressings for non-healing wounds are medical products, which support all of the above-mentioned pillars of the TIMERS wound care strategy. The wide range of commercially available active dressings allows choosing the optimum product for the treatment of a specific wound type at any stage. There are dressings supporting wound debridement, a number of dressings keeping the wound environment moist and certain types improving the epithelization process. The addition of antimicrobial compounds allows limiting wound colonization, prevent infection and support local treatment [84,85]. Examples of antimicrobial dressings are shown in Figure 2.

### 1.9. Bacterial Cellulose as an Excellent Base Material for Non-Healing Wound Dressings

Bacterial cellulose (BC) is a biopolymer produced by specific bacterial species, among which *Komagataeibacter xylinus* is considered the most effective one [97,98]. BC purified from bacteria is a structure composed of glucose chains, organized into parallel structures, which form nanofibers. The crosslinked structure of BC affects its mechanical strength and, thanks to its hydrophilic nature, provides BC with a high ability to absorb fluids. Thanks to its high purity (no hemicellulose, pectins, waxes typically for plant cellulose), nanofiber and polysaccharide structure, BC is highly biocompatible and does not induce any immunological response [99,100,101]. Several studies have proved that BC also displays very low cytotoxic and genotoxic effects [97,102,103,104,105,106]. Moreover, human enzymes are basically incapable of digesting BC, which is a desirable feature in implantation medicine. BC material can be subjected to autoclaving (to gain sterility) and to a number of in situ and ex situ modifications [99,107,108]. Therefore, BC is of high interest to the medical and pharmaceutical industry and may be applied in wound dressings, bone and cartilage implants, as a material for tissue reinforcements, contact lenses, biosensors, drug delivery systems, hernia meshes or artificial skin [99,100,109].

Regarding dressings for wound healing, it should be stressed that BC can absorb high amounts of exudate and keep the wound environment moist. Owing to BC’s highly adhesive properties, application and removal are painless and do not damage the newly healed tissue. Native BC is transparent, which allows controlling of wound conditions without removing the dressing. As mentioned above, BC is non-cytotoxic and non-genotoxic, has high biocompatibility and does not cause allergies [97,99,100,109]. Studies have proved that the application of BC dressings can reduce wound pain, accelerate and facilitate re-epithelization, reduce total healing time and visibility of scars [110,111,112].

**Figure 3:**
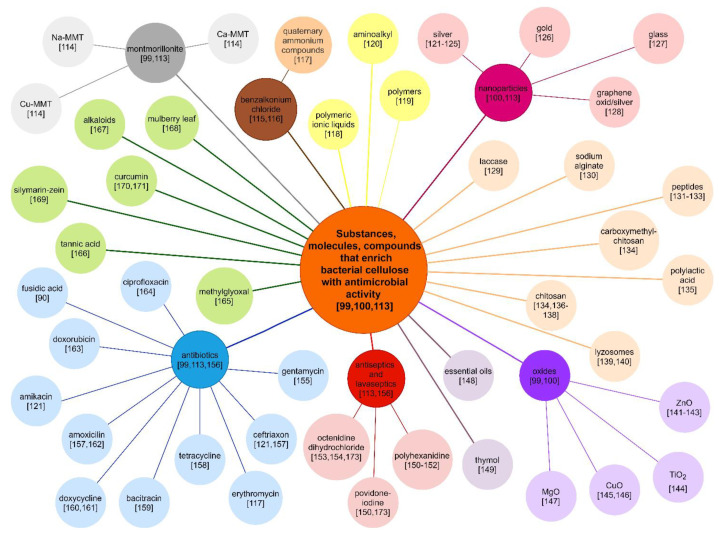
Examples of compounds/substances/molecules introduced to bacterial cellulose (BC) as an antimicrobial additive. MgO—magnesium oxide; CuO—copper oxide; TiO_2_—titanium dioxide; ZnO—zinc oxide; Na-MMT—sodium montmorillonite; Ca-MMT—calcium montmorillonite; Cu-MMT—copper montmorillonite [92,99,100,113,114,115,116,117,118,119,120,121,122,123,124,125,126,127,128,129,130,131,132,133,134,135,136,137,138,139,140,141,142,143,144,145,146,147,148,149,150,151,152,153,154,155,156,157,158,159,160,161,162,163,164,165,166,167,168,169,170,171,172,173].

The studies listed above have shown that bacterial cellulose is safe for use as a dressing for non-healing wounds. Native BC has no antimicrobial activity. However, BC’s ability to absorb high amounts of fluids and wide possibilities of its modification enable enrichment of BC with antimicrobial substances. There are some commercially available BC dressings—some of them made of native cellulose—But there are also dressings containing such substances as sodium hyaluronate or antimicrobial polyhexanide, or chlorhexidine. The interest in BC functionalization with various antimicrobial compounds is constantly growing. The examples of compounds, substances and molecules that are the subject of research (as additives to BC affecting its bactericidal activity) are presented in Figure 3 [92,99,100,113,114,115,116,117,118,119,120,121,122,123,124,125,126,127,128,129,130,131,132,133,134,135,136,137,138,139,140,141,142,143,144,145,146,147,148,149,150,151,152,153,154,155,156,157,158,159,160,161,162,163,164,165,166,167,168,169,170,171,172,173].

To the authors’ best knowledge, there is presently only one commercially available BC dressing displaying antimicrobial activity (thanks to chemisorption with PHMB).

Therefore, in this research, we have evaluated the antimicrobial and antibiofilm activity of bacterial cellulose dressings enriched ex situ with other commonly used antimicrobial compounds (OCT, PVP-I, CHX, EL and SOH) against biofilm-forming nosocomial pathogens. Moreover, we have provided a rich set of control settings (BC chemisorbed with PHMB; dressing chemisorbed with active silver) to get a broad picture of the phenomena observed.

## 2. Results

### 2.1. Evaluation of Test Strain Resistance Mechanisms

Out of 57 strains, 29 (50.8%) had one or more resistance mechanisms. All 6 *S. aureus* strains were methicillin-resistant, and all clinical strains of *S. aureus* were MLS_B_ (+). None of the *S. aureus* strains were vancomycin-resistant. Only one clinical strain of *S. epidermidis* was MLS_B_ (+). All clinical strains of *E. faecium* were vancomycin-resistant and HLAR (+). Among *K. pneumoniae*, one clinical strain was KPC (+), two clinical strains were MBL (+) and OXA-48 (+) and all clinical strains were ESBL (+). One of the clinical *K. pneumoniae* strains showed all the investigated resistance mechanisms. Two clinical strains of *E. coli* were ESBL (+). The reference strain and four clinical strains of *P. aeruginosa* were MBL (+). Three clinical strains of *E. cloacae* were MBL (+), four were ESBL (+), and two were OXA-48 (+). None of the *A. baumannii* strains had resistance mechanisms. Detailed data are summarized in Table S1 of the Supplementary Materials.

### 2.2. Comparison of the Amount of Formed Biofilm and Metabolic Activity of Bacteria/Yeast Cells in Biofilm Structure

**Figure 4:**
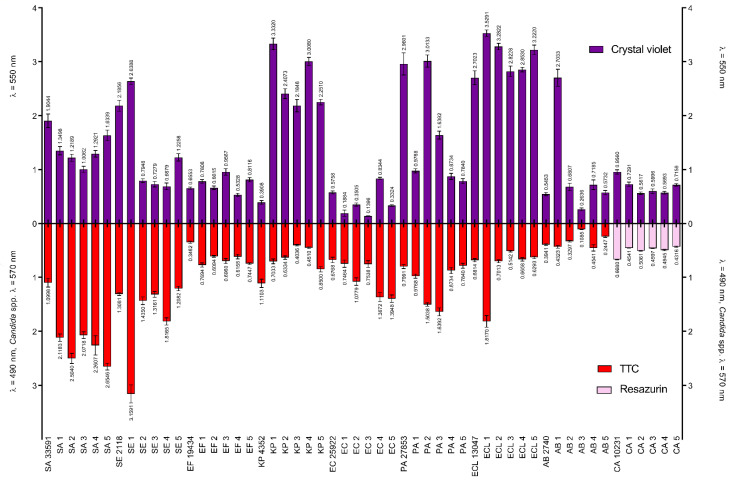
Comparison of metabolic activity of cells in the biofilm to total biofilm mass of the analyzed strains. The amount of formed biofilm was measured using crystal violet (λ = 550 nm), and biofilm metabolic activity was measured with 2, 3, 5- triphenyl tetrazolium chloride (TTC, λ = 490 nm), and with resazurin (λ = 570 nm, only *Candida* spp.). Tested strains: SA—*Staphylococcus aureus*, SE—*Staphylococcus epidermidis*, EF—*Enterococcus faecium*, KP—*Klebsiella pneumoniae*, EC—*Escherichia coli*, PA—*Pseudomonas aeruginosa*, ECL—*Enterobacter cloacae*, AB—*Acinetobacter baumannii*, CA—*Candida albicans*.

The strains, which produced the highest amount of biofilm biomass were: ECL1 > KP1 > ECL2 > ECL5 > PA2. The weakest biofilm production was shown by strains: EC3, EC1, AB3, EC5 and EC2. Biofilm formed by strains: SE1, SA5, SA2 and SA1 showed the highest metabolic activity, while the lowest metabolic activity was displayed by AB3, AB5, AB2 and by the reference strain *E. faecium* ATCC 19434. *C. albicans* ATCC 10231 was the most metabolically active among the tested yeast strains, followed by (in descending order): CA2, CA4, CA3. CA1 and CA5. The results of both experiments are shown in Figure 4.

Noteworthy, the pooled amount of biofilm biomass (Figure 4, upper part) of Gram-negative species was statistically higher (Mann-Whitney (M-W) test, *p* < 0.001) than the analogical parameter recorded for Gram-positive species. The opposite trend was observed concerning metabolic activity; the biofilms of Gram-negative species were in total significantly less active than the biofilms of Gram-positive species (M-W test, *p* < 0.001).

### 2.3. Evaluation of Minimal Inhibitory Concentration (MIC) and Minimal Biofilm Eradication Concentration (MBEC) of Tested Substances

#### 2.3.1. MIC

**Figure 5:**
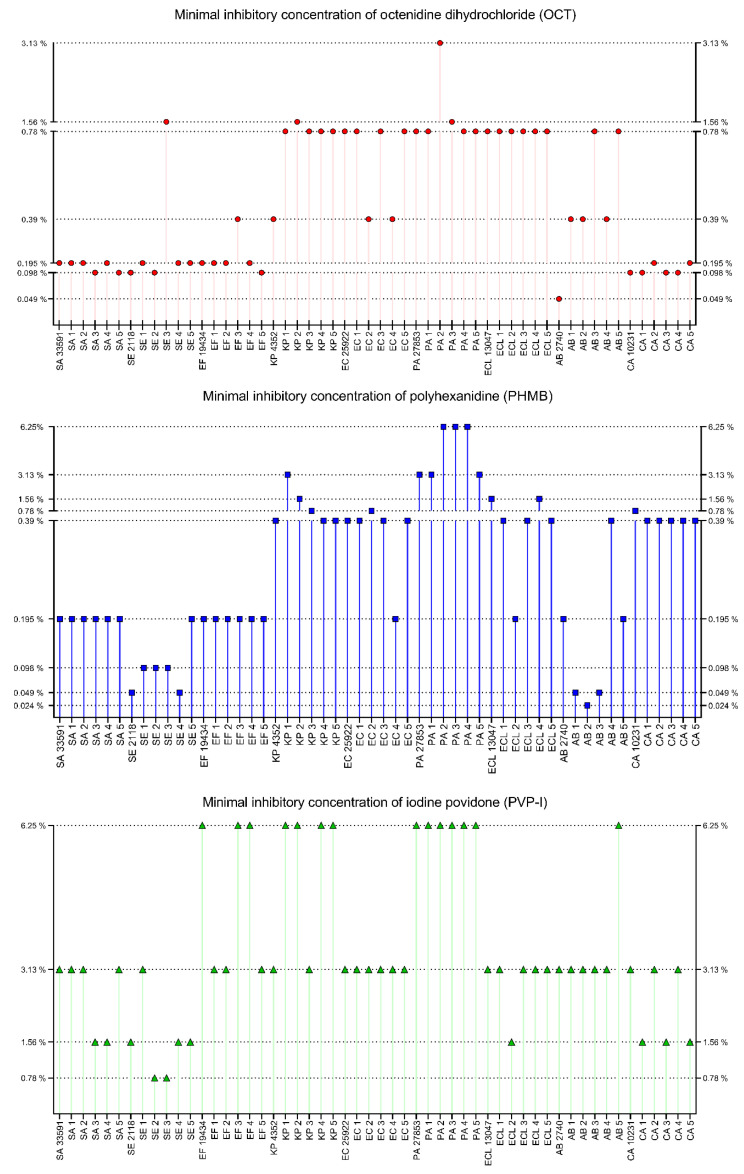
Minimal inhibitory concentrations of OCT, PHMB and PVP-I, presented as a percentage of working solutions (concentration provided by the manufacturer). Tested compounds: OCT—octenidine dihydrochloride, PHMB—polyhexanide, PVP-I—povidone-iodine (Octenilin^®^ 0.05% of OCT, Prontosan^®^ 0.1% of PHMB and Braunol^®^ 7.5% of PVP-I). Tested strains: SA—*Staphylococcus aureus*, SE—*Staphylococcus epidermidis*, EF—*Enterococcus faecium*, KP—*Klebsiella pneumoniae*, EC—*Escherichia coli*, PA—*Pseudomonas aeruginosa*, ECL—*Enterobacter cloacae*, AB—*Acinetobacter baumannii*, CA—*Candida albicans*.

All substances were tested in the range of 50–0.098% of working solution (concentration provided by the manufacturer). If MIC was not observed, the concentration range was extended to 0.0015%. OCT inhibited the growth of all tested microorganisms within the tested concentration range. MIC values were observed between 0.049% and 3.13% of OCT working solution (0.5% of OCT). The strongest effects (the lowest MIC) were observed for *A. baumannii* PMC 2740 strain (0.049%), *C. albicans*, *S. aureus*, *S. epidermidis* and *E. faecium* strains (0.098–0.195%, except for two strains: SE3 and EF3). The weakest effects were observed for PA2 (3.13%), PA3, KP2 and SE3 (1.56%), and intermediate effects for EF3, *K. pneumoniae* ATCC 4352, EC2, EC4, AB1, AB2 and AB4 (0.39%) and for the rest of the tested strains (*n* = 20, 0.78%). OCT acted stronger on Gram-positive cocci and yeast than on Gram-negative rods. PHMB was effective against all tested strains. The range of product working solution for MIC was 0.024–6.25%. The strongest effects were observed for AB2 (0.024%), AB1, AB3, *S. epidermidis* PCM 2118 and SE4 (0.049%) and for SE1, SE2 and SE3 (0.098%). The weakest effects were observed for PA2, PA3, PA4 (6.25%), KP1, PA1, PA5, *P. aeruginosa* ATCC 27853 (3.13%) and for KP2, ELC4, *E. cloacae* ATCC 13047 (1.56%). Intermediate effects were observed for KP3, EC2, *C. albicans* ATCC 10231 (0.78%), *K. pneumoniae* ATCC 4352, KP4, KP5, *E. coli* ATCC 25922, EC1, EC3, EC5, ECL1. ECL3, ECL5, AB4, CA1–5, (0.39%) and 0.195% for the remaining strains (*n* = 17). PHMB acted better against Gram-positive cocci and *A. baumannii* strains than against the rest of Gram-negative rods and yeast. PVP-I also was effective against all tested strains. MIC values were observed between 0.78% and 6.25% of product working solution. The strongest effects were observed for SE2 and SE3 (0.78%), the weakest for *E. faecium* ATC 19434, EF3, EF4, KP1, KP2, KP4, KP5, *P. aeruginosa* ATCC 27853, PA1–PA5 and AB5 (6.25%) and intermediate for SA3, SA4, *S. epidermidis* PCM 2118, SE4, SE5, ELC2, CA1, CA3, CA5 (1.56%) and for the rest of tested strains (29, strains, 3.13%). The best action of PVP-I was observed against *S. epidermidis* and *C. albicans* strains and the weakest against *P. aeruginosa* and *K. pneumoniae.* The results for OCT, PHMB and PVP-I, are presented in Figure 5.

**Figure 6:**
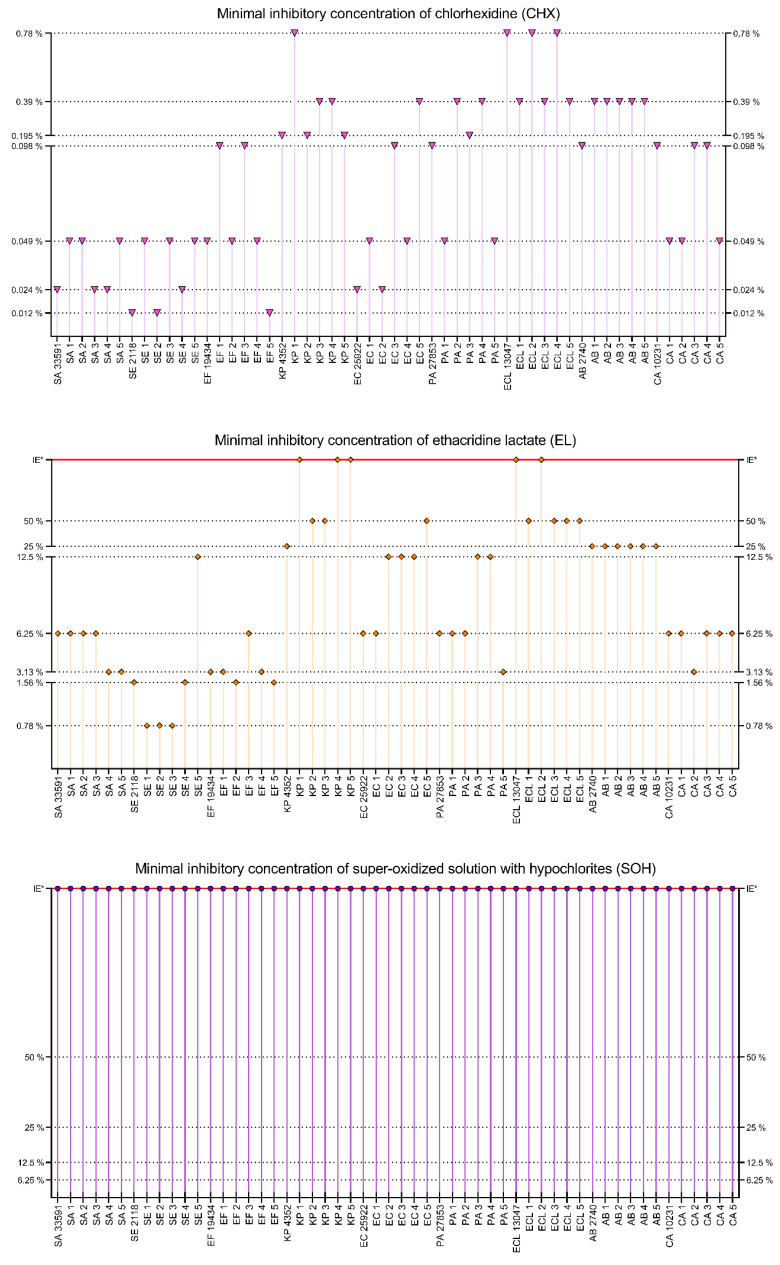
Minimal inhibitory concentrations of CHX, EL and SOH, presented as a percentage of working solutions (concentration provided by the manufacturer). Tested compounds: CHX—chlorhexidine, EL—ethacridine lactate, SOH—super-oxidized hypochlorites solution (water solution of chlorhexidine diluted to 0.5% of CHX, Rivanol^®^ 0.1% of EL and Microdacyn^®^ 0.004% + 0.004% of NaOCl and HOCl). Tested strains: SA—*Staphylococcus aureus*, SE—*Staphylococcus epidermidis*, EF—*Enterococcus faecium*, KP—*Klebsiella pneumoniae*, EC—*Escherichia coli*, PA—*Pseudomonas aeruginosa*, ECL—*Enterobacter cloacae*, AB—*Acinetobacter baumannii*, CA—*Candida albicans*. IE *—tested compound was ineffective in the concentration range 50–0.098%.

CHX was effective against all tested strains. The range of product working solutions for MIC was 0.0031–0.78%. The strongest effects were observed for *S. epidermidis* PCM 2118, SE2, EF5 (0.0031%), *S. aureus* ATCC 33591, SA3, SA4, SE4, *E. coli* ATCC 25922 and EC2 (0.012%). The weakest effects were observed for: KP1, *E. cloacae* ATCC 13047, ECL2, ECL4 (0.78%), KP3, KP4, EC5, PA2, PA4, ECL1, ECL3, ECL5 and AB1–AB5 (0.39%), and intermediate for: *K. pneumoniae* ATCC 4352, KP2, KP5, PA3 (0.195%), EF1, EF3, EC3, *P. aeruginosa* ATCC 27853, *A. baumannii* PCM 2740, *C. albicans* ATCC 10231, CA3, CA4 (0.098%) and the rest of the tested strains (*n* = 16, 0.049%). The highest activity of CHX was observed for *Staphylococci* and the weakest for *E. cloacae*, *A. baumannii* and *K. pneumoniae.* EL was effective for 49/54 strains in the tested concentration range. Strains KP1, KP4, KP5, *E. cloacae* ATCC 13047 and ECL2 were resistant to EL in 50% concentration. Against the rest of the tested strains, EL was effective in a concentration range from 0.78% to 50%. The strongest effects were observed for: SE1, SE2, SE3 (0.78%), *S. epidermidis* PCM 2118, SE4, EF2 and EF5 (1.56%). The weakest effects were observed for KP2, KP3, EC5, ECL1, ECL3, ECL4, ECL5 (50%), *K. pneumoniae* ATCC 4352, *A. baumannii* PCM 2740 and AB1–AB5 (25%) and intermediate for SE5, EC2, EC3, EC4, PA3, PA4 (12.5%), SA4, SA5, *E. faecium* ATCC 19434, EF1, EF4, PA5, CA2 (3.13%) and for the rest of the tested strains (15 strains, 6.25%). The best activity of EL was observed for *S. epidermidis and E. faecium* and the weakest for *E. cloacae*, *A. baumannii* and *K. pneumoniae.* SOH was not effective against any of the tested strains in the concentration range from 50 to 0.098%. The results for CHX, EL and SOH, are presented in Figure 6.

**Figure 7:**
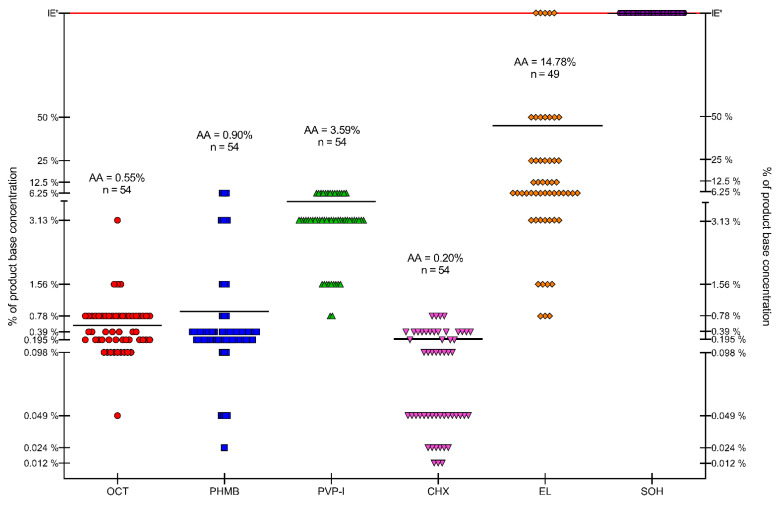
Distribution of MIC values of the tested substances in the pool of strains. OCT—octenidine dihydrochloride, PHMB—polyhexanide, PVP-I—povidone-iodine, CHX—chlorhexidine, EL—ethacridine lactate, SOH—super-oxidized hypochlorites solution. AA—arithmetic average of MIC values for all strains, n—number of strains included in AA, IE *—compound ineffective in tested concentration range.

The distribution of results for all tested compounds is shown in Figure 7. The lowest MIC values were observed for CHX, with an average MIC of 0.2% (*n* = 54). OCT and PHMB demonstrated lower activity, with an average MIC of 0.55% (*n* = 54) and 0.90% (*n* = 54), respectively. Average MIC for PVP-I was 3.59% (*n* = 54). The weakest compounds were EL and SOH. The average MIC for EL was 23.60% (*n* = 49, no antimicrobial effect against 5 strains), and no antimicrobial effect was observed for SOH. PHMB and EL gave the most scattered results. Generally, Gram-positive cocci were more susceptible to the applied solutions than Gram-negative rods. Yeast showed a varied distribution of susceptibility concerning the analyzed compounds.

The results presented in Figure 7 show that EL and SOH were statistically less effective (against all pathogens tested) than OCT, PHMB, PVP-I, CHX (Kruskal-Wallis (K-W) test, *p* < 0.0001). No significant difference was recorded between OCT, PHMB and CHX; in turn, all three of the aforementioned antiseptics displayed significantly higher activity (in this type of experimental setting) than PVP-I (K-W test, *p* < 0.0001).

#### 2.3.2. MBEC

All the substances were tested in the range of 100–0.195% of working solutions. The application of OCT led to biofilm eradication in 53 out of 54 cases; the single exception was the biofilm of the pseudomonal PA2 strain. The range of effective concentrations was broad, starting from 1.56% and ending at 100% of the product working solution. The best results were observed against AB3 (1.56%), SE2, SE4, *E. faecium* ATCC 19434, EF1, EF4, AB5, CA2, CA3, CA5 (3.13%), *S. epidermidis* PCM 2118, SE5, EF2, EF3, EF5, *C. albicans* ATCC 10231, CA1 and CA4 (6.25%). The weakest results were observed against KP4, KP5, *E. coli* ATCC 25922, EC4, EC5, *P. aeruginosa* ATCC 27853, PA1, PA3, PA4, *E. cloacae* ATCC 13047, ECL1, ECL2, ECL3, ECL5 (100%), KP1, KP2, KP3, EC2, PA5, ECL4, *A. baumannii* PCM 2740, AB4 (50%), and intermediate results were recorded against SA1, EC1, EC3, AB1, AB2 (25%) and for the remaining strains (8 strains, 12.5%). The strongest eradication was observed for *S. epidermidis*, *E. faecium* and *C. albicans* strains and the weakest for Gram-negative rods. PHMB eradicated the biofilm of all tested pathogens. The effective concentration range was 1.56–100%. The best results were observed for AB3 (1.56%), CA2, CA5 (6.25%) and the weakest for *S. aureus* ATCC 6538, SA1, PA1, PA2, PA3, PA4 (100%), SA2–SA5, SE3, KP3, KP4, EC3, EC4, *P. aeruginosa* ATCC 27853, PA4, *E. cloacae* ATCC 13047, ECL1 and ECL5 (50%). Intermediate results were observed for SA2, SE4, *E. faecium* ATCC 19434, EF4, *K. pneumoniae* ATCC 4352, AB5, *C. albicans* ATCC 10231, CA1, CA3, CA4 (12.5%) and for the rest of the tested strains (21 strains, 25%). A slightly stronger activity was observed for *S. epidermidis*, *E. faecium* and *C. albicans* than for the rest of the species. The worst results were observed for *P. aeruginosa* and *S. aureus* strains.

**Figure 8:**
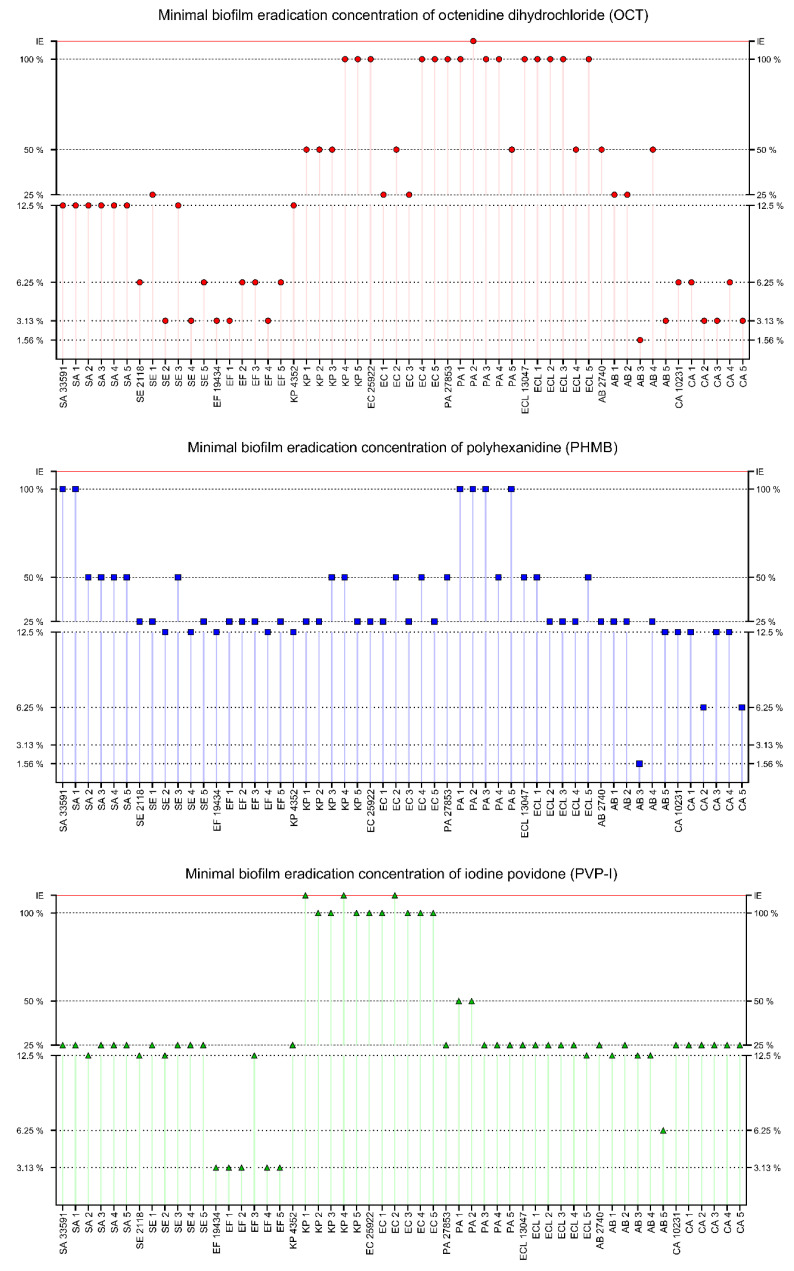
Minimal biofilm eradication concentrations of OCT, PHMB and PVP-I, presented as a percentage of working solutions (concentration provided by the manufacturer). Tested compounds: OCT—octenidine dihydrochloride, PHMB—polyhexanide, PVP-I—povidone-iodine (Octenilin^®^ 0.05% of OCT, Prontosan^®^ 0.1% of PHMB and Braunol^®^ 7.5% of PVP-I). Tested strains: SA—*Staphylococcus aureus*, SE—*Staphylococcus epidermidis*, EF—*Enterococcus faecium*, KP—*Klebsiella pneumoniae*, EC—*Escherichia coli*, PA—*Pseudomonas aeruginosa*, ECL—*Enterobacter cloacae*, AB—*Acinetobacter baumannii*, CA—*Candida albicans*. IE—compound was ineffective.

PVP-I was ineffective in three cases: KP1, KP4 and EC2. For the rest of the strains, MBEC values were in the range of 3.13–100%. The strongest effects were observed for *E. faecalis* strains (3.13%, except for EF3) and for AB5 (6.25%). The working solution of PVP-I (100%) was effective against KP2, KP3, KP5 and *E. coli* strains (except for EC2). For strains PA1 and PA2, PVP-I was effective at the concentration of 50%, and for SA2, *S. epidermidis* PCM 2118, SE2, EF3, ECL5, AB1, AB3, AB4 at the concentration of 12.5%. For the remaining strains (27 strains), PVP-I was effective at a concentration of 25%. The strongest action of PVP-I was observed against *E. faecium* strains and the weakest against *K. pneumoniae* and *E. coli*. The results of minimal biofilm eradication concentrations assay for OCT, PHMB and PVP-I are presented in Figure 8.

**Figure 9:**
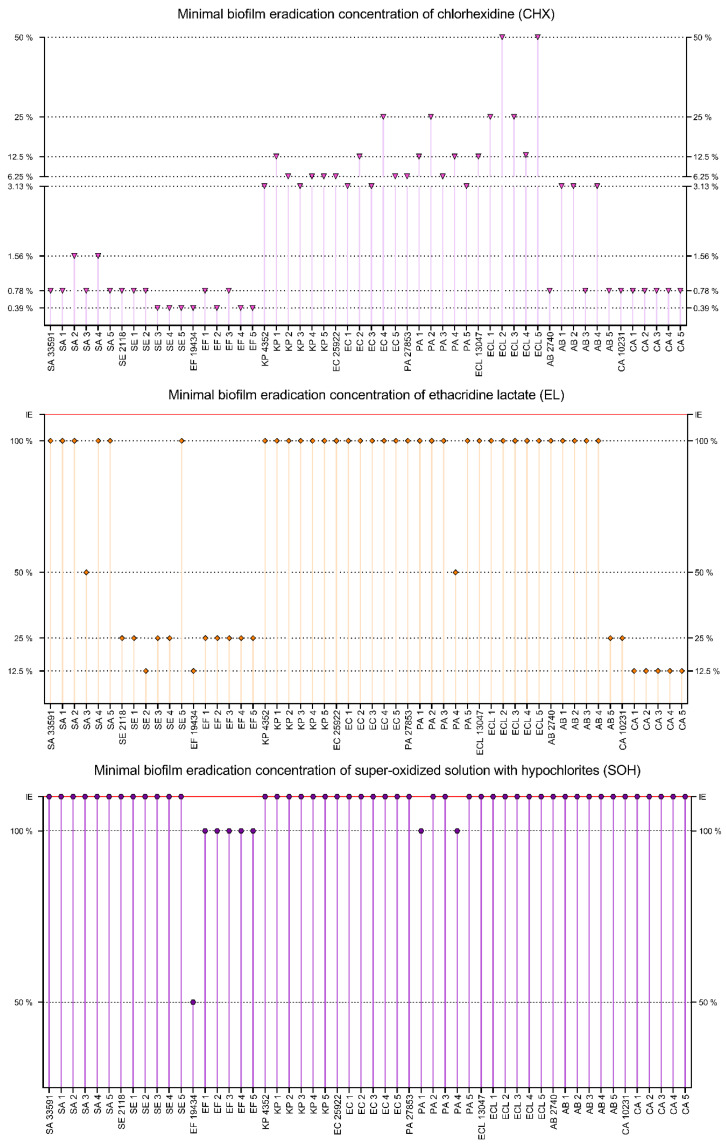
Minimal biofilm eradication concentrations of OCT, PHMB and PVP-I, presented as a percentage of working solutions (concentration provided by the manufacturer). Tested compounds: CHX—chlorhexidine, EL—ethacridine lactate, SOH—super-oxidized hypochlorites solution (water solution of chlorhexidine diluted to 0.5% of CHX, Rivanol^®^ 0.1% of EL and Microdacyn^®^ 0.004% + 0.004% of NaOCl and HOCl). Tested strains: SA—*Staphylococcus aureus*, SE—*Staphylococcus epidermidis*, EF—*Enterococcus faecium*, KP—*Klebsiella pneumoniae*, EC—*Escherichia coli*, PA—*Pseudomonas aeruginosa*, ECL—*Enterobacter cloacae*, AB—*Acinetobacter baumannii*, CA—*Candida albicans*. IE—compound was ineffective.

CHX eradicated the biofilm of all tested strains. The effective concentration range was 0.39–50%. The best results were observed for SE3, SE4, SE5, *E. faecium* ATCC 19434, EF2, EF4, EF5 (0.39%), *S. aureus* ATCC 6538, SA1, SA3, SA5, *S. epidermidis* PCM 2118, SE1, SE2, EF1, EF3, *A. baumannii* PCM 2740, AB3, AB5, *C. albicans* ATCC 10231, CA1–CA5 (0.78%), SA2 and SA4 (1.56%). Effectiveness of a high concentration of CHX was observed against ECL2, ECL5 (50%), EC4, PA2, ECL1, ECL3 (25%) and against KP1, EC2, PA1, PA4, *E. cloacae* ATCC 13047, ECL4 (12.5%). Intermediate results were observed for KP2, KP4, KP5. *E. coli* ATCC 25922, EC5, *P. aeruginosa* ATCC 27853, PA3 (6.25%) and for the rest of the tested strains (8 strains, 3.13%). CHX showed stronger activity against Gram-positive bacteria and yeast than against Gram-negative bacteria. The best results were observed for *E. faecium*, *S. epidermidis* and *C. albicans*, while the worst for *E. cloacae* and *P. aeruginosa*. EL eradicated biofilm in all cases in the concentration range 12.5–100%. The lowest active concentration was observed for SE2, *E. faecium* ATCC 19434 and CA1–CA5 (12.5%), while the highest concentrations were needed for *S. epidermidis* PCM 2118, SE1, SE3, SE4, EF1–EF5, AB5, *C. albicans* ATCC 10231 (25%), SA3, PA4 (50%) and for the rest of the tested strains (34 strains, 100%). The best activity of EL was observed against *C. albicans*, *S. epidermidis* and *E. faecium* and the worst against Gram-negative rods and *S. aureus*. SOH was effective against eight strains only. The effective SOH concentration for *E. faecium* ATCC 19434 was 50% and 100% for EF1–EF5, PA1, PA4. The biofilms formed by the rest of the tested strains (46 strains) were not eradicated by SOH, even at a working solution. The results of minimal biofilm eradication concentrations assay for CHX, EL and SOH are presented in Figure 9.

**Figure 10:**
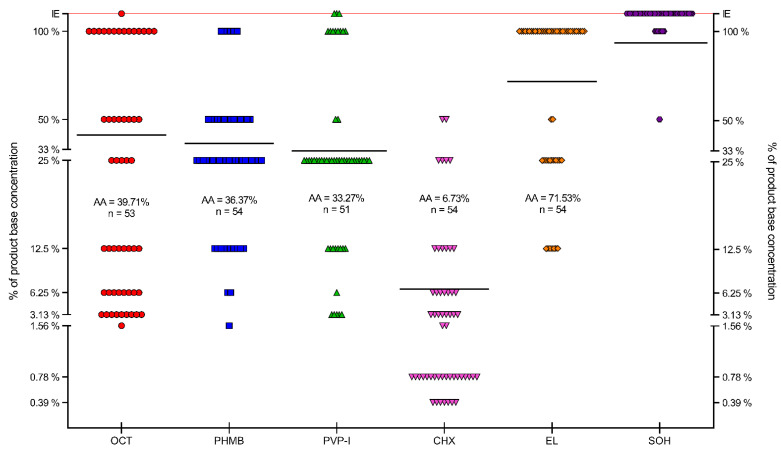
Distribution of MBEC values of the tested substances in the pool of strains. OCT—octenidine dihydrochloride, PHMB—polyhexanide, PVP-I—povidone-iodine, CHX—chlorhexidine, EL—ethacridine lactate, SOH—super-oxidized hypochlorites solution. AA—arithmetic average of MBEC values for all strains (black lines), n—number of strains included in AA, IE—compound ineffective.

The distribution of MBEC values for all strains is presented in Figure 10. The lowest MBEC values were observed for CHX, with an average MBEC of 6.73% (*n* = 54). PHMB and PVP-I demonstrated lower activity with an average MBEC of 36.37% (*n* = 54) and 33.27% (*n* = 51, no antimicrobial activity against 3 strains), respectively. The average MBEC for OCT was 39.71% (*n* = 53, no antimicrobial activity against 1 strain). The weakest compounds were EL and SOH. The average MBEC for EL was 71.53% (*n* = 54) and for SOH 93.75% (*n* = 8, no antimicrobial activity against 46 strains). For all compounds, the scatter of results was significant. Generally, biofilms formed by *Enterococcus faecium* and *Staphylococcus epidermidis* were more susceptible to the applied solutions than biofilms formed by *Staphylococcus aureus*, Gram-negative rods, or yeast. Regarding statistical significance, SOH and EL displayed significantly lower activity than the rest of the analyzed antiseptics (M-W test, *p* < 0.001), while CHX displayed significantly higher activity than OCT, PHMB and PVP-I. In turn, the activities of OCT, PHMB and PVP-I were comparable.

### 2.4. Evaluation of Antimicrobials’ Activity by Modified Disk-Diffusion Method

**Figure 11:**
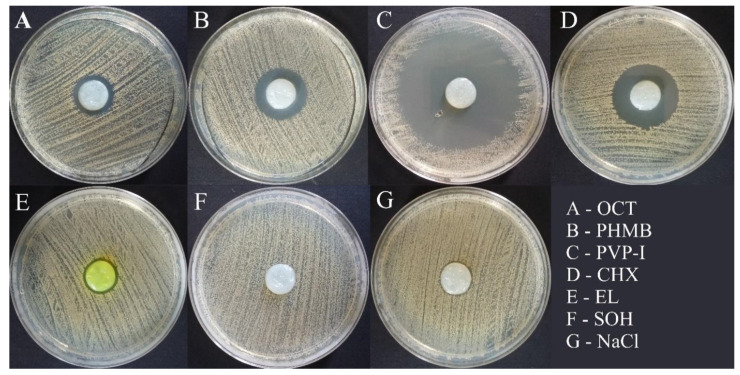
Growth inhibition zones around bacterial cellulose dressing are chemisorbed with the tested compounds. OCT—octenidine dihydrochloride, PHMB—polyhexanide, PVP-I—povidone-iodine, CHX—chlorhexidine, EL—ethacridine lactate, SOH—super-oxidized hypochlorites solution, NaCl—Sterile saline as a negative control. The picture shows results for the *Staphylococcus aureus* ATCC 33591 reference strain.

Exemplary results of the modified disc-diffusion method (applying BC as an antiseptic carrier) are shown in Figure 11.

The BC dressings chemisorbed with PVP-I and CHX were the most effective against *S. aureus* (the growth inhibition zone was 2382.16 mm^2^ vs. 660.37 mm^2^, respectively). Intermediate results were observed for BC chemisorbed with PHMB and OCT (the growth inhibition zone was 189.30 mm^2^ vs. 137.77 mm^2^, respectively) and the weakest for BC chemisorbed with EL and SOH (the growth inhibition zone was 74.94 mm^2^ vs. 20.20 mm^2^, respectively). BC dressing with EL was ineffective against one strain (SA1) and BC dressing with SOH against three strains (SA1, SA3 and SA5). BC dressings chemisorbed with PVP-I and CHX were the most effective against *S. epidermidis* (growth inhibition zone was 2038.13 mm^2^ vs. 920.60 mm^2^, respectively). Intermediate results were observed for BC chemisorbed with PHMB and EL (the growth inhibition zone was 311.02 mm^2^ vs. 168.23 mm^2^, respectively) and the weakest for BC chemisorbed with OCT (the growth inhibition zone was 103.11 mm^2^). BC chemisorbed with SOH was not effective against any *S. epidermidis* strain. BC dressings chemisorbed with PVP-I and CHX were the most effective against *E. faecium* (the growth inhibition zone was 1511.67 mm^2^ vs. 807.87 mm^2^, respectively). Intermediate results were observed for BC chemisorbed with PHMB and EL (the growth inhibition zone was 129.22 mm^2^ vs. 118.33 mm^2^, respectively) and the weakest for BC chemisorbed with OCT (the growth inhibition zone was 74.79 mm^2^). BC chemisorbed with SOH was not effective against any *E. faecium* strain. BC dressings chemisorbed with PVP-I and CHX were the most effective against *K. pneumoniae* (the growth inhibition zone was 918.70 mm^2^ vs. 393.81 mm^2^, respectively). Intermediate results were observed for BC chemisorbed with PHMB and OCT (the growth inhibition zone was 144.54 mm^2^ vs. 47.04 mm^2^, respectively) and the weakest for BC chemisorbed with EL (the growth inhibition zone was 1.79 mm^2^). BC chemisorbed with SOH was not effective against any *K. pneumoniae* strain. BC dressing chemisorbed with EL was ineffective against three strains (*K. pneumoniae* ATCC 4352, KP3 and KP4).

BC dressings chemisorbed with PVP-I and CHX were the most effective against *E. coli* (the growth inhibition zone was 720.76 mm^2^ vs. 480.38 mm^2^, respectively). Intermediate results were observed for BC chemisorbed with PHMB and OCT (the growth inhibition zone was 186.41 mm^2^ vs. 76.93 mm^2^, respectively) and the weakest for BC chemisorbed with EL (the growth inhibition zone was 16.63 mm^2^). BC chemisorbed with SOH was not effective against any *E. coli* strain. BC chemisorbed with EL was not effective against two strains (EC1 and EC5). BC dressings chemisorbed with CHX and PVP-I were the most effective against *P. aeruginosa* (the growth inhibition zone was 381.68 mm^2^ vs. 278.79 mm^2^, respectively). Intermediate results were observed for BC chemisorbed with OCT and PHMB (the growth inhibition zone was 36.32 mm^2^ vs. 26.27 mm^2^, respectively) and the weakest for BC chemisorbed with EL (the growth inhibition zone was 6.52 mm^2^). BC chemisorbed with SOH was not effective against any *P. aeruginosa* strain. BC chemisorbed with EL was ineffective against two strains (PA3 and PA5). BC dressings chemisorbed with PVP-I and CHX were the most effective against *E. cloacae* (the growth inhibition zone was 661.85 mm^2^ vs. 458.76 mm^2^, respectively). Intermediate results were observed for BC chemisorbed with PHMB and OCT (the growth inhibition zone was 145.17 mm^2^ vs. 21.23 mm^2^, respectively) and the weakest for BC chemisorbed with EL (the growth inhibition zone was 2.20 mm^2^). BC chemisorbed with SOH was not effective against any *E. cloacae* strain. BC chemisorbed with EL was effective against only one strain (ECL1). BC dressings chemisorbed with PVP-I and CHX were the most effective against *A. baumannii* (the growth inhibition zone was 1320.36 mm^2^ vs. 382.12 mm^2^, respectively). Intermediate results were observed for BC chemisorbed with PHMB and OCT (the growth inhibition zone was 107.64 mm^2^ vs. 75.14 mm^2^, respectively) and the weakest for BC chemisorbed with EL (the growth inhibition zone was 8.22 mm^2^). BC chemisorbed with SOH was not effective against any *A. baumannii* strain. BC chemisorbed with EL was ineffective against three strains (AB3, AB4 and AB5). BC dressings chemisorbed with PVP-I, CHX and PHMB were the most effective against *C. albicans* (the growth inhibition zone was 2817.70 mm^2^ vs. 1025.84 mm^2^ vs. 1021.89 mm^2^, respectively). Intermediate results were observed for BC chemisorbed with OCT and EL (the growth inhibition zone was 243.91 mm^2^ vs. 156.56 mm^2^, respectively). BC chemisorbed with SOH was not effective against any *C. albicans* strain.

Graphic representations of results for every group of strains are presented in Figure S1–S9 in the Supplementary Materials.

**Figure 12:**
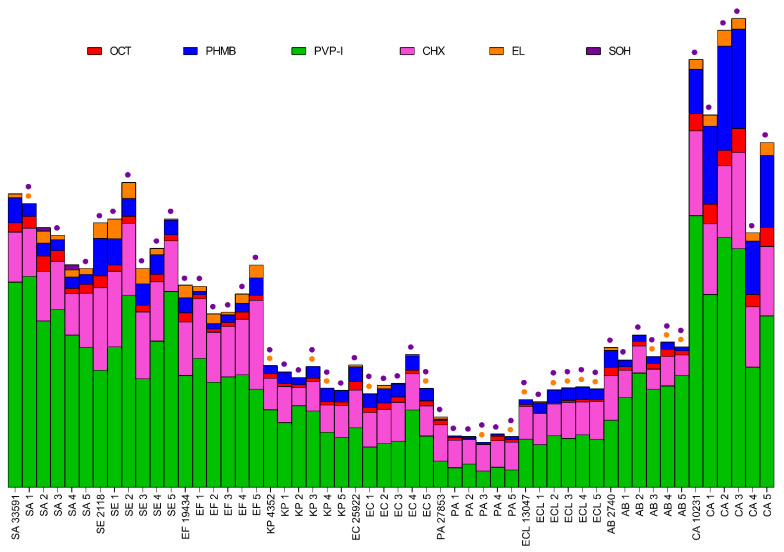
Comparison of surface areas of growth inhibition zones (mm^2^). SA—Staphylococcus aureus, SE—Staphylococcus epidermidis, EF—Enterococcus faecium, KP—Klebsiella pneumoniae, EC—Escherichia coli, PA—Pseudomonas aeruginosa, ECL—Enterobacter cloacae, AB—Acinetobacter baumannii, CA—Candida albicans. Tested substances: OCT—octenidine dihydrochloride, PHMB—polyhexanide, PVP-I—povidone-iodine, CHX—chlorhexidine, EL—ethacridine lactate, SOH—super-oxidized hypochlorites solution. Bar size represents the average surface area (mm^2^) of growth inhibition zones. Dots indicate ineffective compounds, color of dot corresponds to the tested compounds.

Figure 12 presents a comparison of the area of growth inhibition zones of all tested strains.

The most effective (concerning antimicrobial effect) compound released from BC was PVP-I and the difference between this and other antiseptics was statistically significant (K-W test, *p* < 0.001). Noteworthy, SOH antiseptic was effective against only three strains (*S. aureus* ATCC 6538, SA 2 and SA 4), while for other tested strains, no growth inhibition zones were observed.

OCT showed the strongest effect against *C. albicans* (average growth inhibition zone (AZ) = 243.91 mm^2^), *S. aureus* (AZ = 137.77 mm^2^) and *S. epidermidis* (AZ = 103.11 mm^2^). Intermediate, comparable activity was observed for *E. coli* (AZ = 76.93 mm^2^), *A. baumannii* (AZ = 75.14 mm^2^) and *E. faecium* (AZ = 74.79 mm^2^). The weakest activity was observed against *K. pneumoniae* (AZ = 47.04 mm^2^), *P. aeruginosa* (AZ = 36.32 mm^2^) and *E. cloacae* (AZ = 21.23 mm^2^).

PHMB showed the strongest effect against *C. albicans* (AZ = 1021.89 mm^2^) and *S. epidermidis* (AZ = 311.02 mm^2^). Intermediate activity was observed for *S. aureus* (AZ = 189.30 mm^2^), *E. coli* (AZ = 186.41 mm^2^), *E. cloacae* (AZ = 145.17 mm^2^), *K. pneumoniae* (AZ = 144.54 mm^2^) and *E. faecium* (AZ = 129.22 mm^2^). The weakest activity was observed for *A. baumannii* (AZ = 107.64 mm^2^) and *P. aeruginosa* (AZ = 26.27 mm^2^).

PVP-I showed the strongest effect against *C. albicans* (AZ = 2817.70 mm^2^), *S. aureus* (AZ = 2382.16 mm^2^) and *S. epidermidis* (AZ = 2038.13 mm^2^). Intermediate activity was observed against *E. faecium* (AZ = 1511.67 mm^2^), *A. baumannii* (AZ = 1320.36 mm^2^), *K. pneumoniae* (AZ = 918.70 mm^2^), *E. coli* (AZ = 720.76 mm^2^) and *E. cloacae* (AZ = 661.85 mm^2^). The weakest activity was observed against *P. aeruginosa* (AZ = 278.79 mm^2^).

CHX showed the strongest effect against *C. albicans* (AZ = 1025.84 mm^2^), *S. epidermidis* (AZ = 920.60 mm^2^) and *E. faecium* (AZ = 807.87 mm^2^). Intermediate activity was observed against *S. aureus* (AZ = 660.37 mm^2^), *E. coli* (AZ = 480.38 mm^2^) and *E. cloacae* (AZ = 458.76 mm^2^). The weakest activity was observed against *K. pneumoniae* (AZ = 393.81 mm^2^), *A. baumannii* (AZ = 382.12 mm^2^) and *P. aeruginosa* (AZ = 381.68 mm^2^).

EL showed the strongest effect against *S. epidermidis* (AZ = 168.23 mm^2^), *C. albicans* (AZ = 156.56 mm^2^) and *E. faecium* (AZ = 118.33 mm^2^). Intermediate activity was observed against *S. aureus* (AZ = 74.94 mm^2^) and *E. coli* (AZ = 16.36 mm^2^). The weakest activity was observed against *A. baumannii* (AZ = 8.22 mm^2^), *P. aeruginosa* (AZ = 6.52 mm^2^), *E. cloacae* (AZ = 2.20 mm^2^). and *K. pneumoniae* (AZ = 1.79 mm^2^).

SOH was effective only against *S. aureus* (AZ = 20.20 mm^2^). There were no growth inhibition zones observed in the case of other bacterial species and yeast.

**Figure 13:**
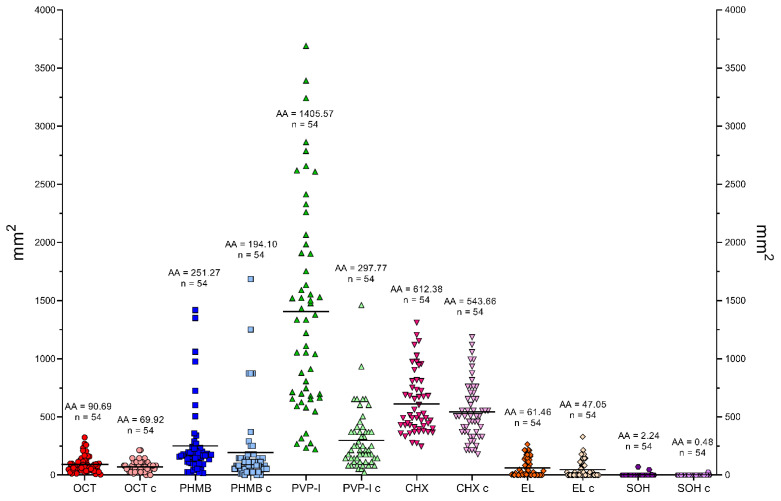
Distribution of average areas of growth inhibition zones (mm^2^). For every compound, there are 2 kinds of probes: chemisorbed BC dressings (OCT—octenidine dihydrochloride, PHMB—polyhexanide, PVP-I—povidone-iodine, CHX—chlorhexidine, EL—ethacridine lactate, SOH—super-oxidized hypochlorites solution) and soaked blotting paper discs compounds as a compound activity control (OCT c—control of OCT activity, PHMB c—control of PHMB activity, PVP-I c—control of PVP-I activity, CHX c—control of CHX activity, EL c—control of EL activity, SOH c—control of SOH activity). AA—arithmetic average of growth inhibition zones areas (black lines), n—number of tested strains (54).

The highest growth inhibition zones were observed for PVP-I (the average growth inhibition zone for all strains (AZ) = 1405.57 mm^2^) and CHX (AZ = 612.38 mm^2^). Smaller zones were obtained for PHMB (AZ = 6.52 mm^2^) and OCT (AZ = 6.52 mm^2^). EL and SOH did not act against every strain. EL was ineffective against 16/54, while SOH against 51/54 strains. These compounds caused the smallest average growth inhibition zones: EL = 61.46 mm^2^ and SOH = 2.24 mm^2^. PVP-I, PHMB and CHX demonstrate the largest dispersion of results. In turn, the standard deviations of mean in the case of OCT, EL and SOH were low. The distribution of average areas of growth inhibition zone is presented in Figure 13. Among the tested species, the most susceptible to the antiseptics were *C. albicans*, *S. epidermidis* and *S. aureus* (AZ = 877.65 mm^2^ vs. 590.18 mm^2^ vs. 577.46 mm^2^, respectively). *E. faecium* and *A. baumannii* showed the weakest susceptibility (AZ = 440.31 mm^2^ vs. 315.58 mm^2^, respectively), along with *K. pneumoniae*, *E. coli* and *E. cloacae* (AZ = 250.98 mm^2^ vs. 246.81 mm^2^ vs. 214.87 mm^2^, respectively). *P. aeruginosa* was the most resistant to all tested antimicrobial compounds (AZ = 121.60 mm^2^). Comprehensive data for all tested strains are summarized in Table S2 and are available in the Supplementary Materials.

### 2.5. Evaluation of Silver Dressings’ Activity Using the Modified Disk Diffusion Method

**Figure 14:**
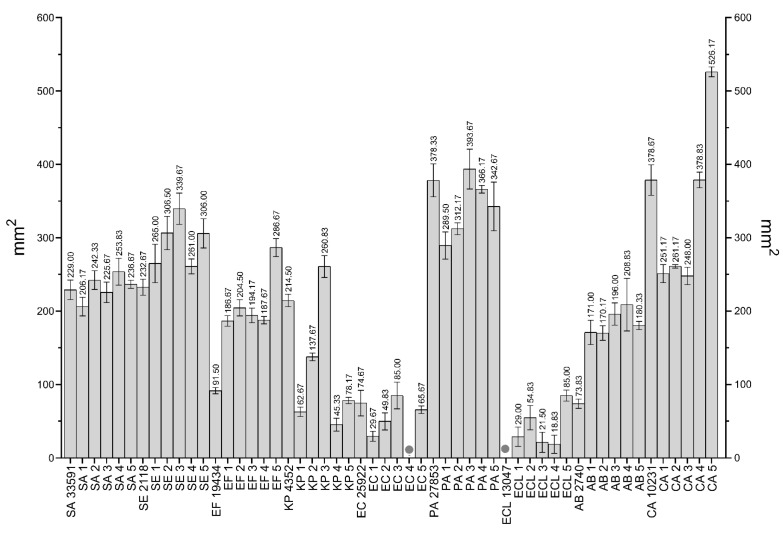
Average growth inhibition zones caused by silver dressing (mm^2^). The antimicrobial agent was silver dressing (Aquacel^®^ Ag, ConvaTec, Berkshire, England). Dots indicate strains against which the silver dressing was ineffective. Tested strains: SA—*Staphylococcus aureus*, SE—*Staphylococcus epidermidis*, EF—*Enterococcus faecium*, KP—*Klebsiella pneumoniae*, EC—*Escherichia coli*, PA—*Pseudomonas aeruginosa*, ECL—*Enterobacter cloacae*, AB—*Acinetobacter baumannii*, CA—*Candida albicans*.

A silver dressing was used to compare the antimicrobial activity of BC dressings chemisorbed with a clinical material of proven antimicrobial activity. Silver dressing was effective against 52/54 strains (96% of the tested strains). No growth inhibition zones were observed for EC4 and *E. cloacae* ATCC 13047. The highest activity of the silver dressing was observed against *P. aeruginosa*, *C. albicans* and *S. epidermidis* strains (average zones of growth inhibition: 347.08 mm^2^, 340.67 mm^2^ and 285.14 mm^2^, respectively). Moderate activity was observed against *S. aureus* (232.28 mm^2^), *E. faecium* (191.86 mm^2^) and *A. baumannii* (166.69 mm^2^) and weak activity against *K. pneumoniae* (133.19 mm^2^), *E. coli* (50.81 mm^2^) and *E. cloacae* (34.86 mm^2^). Average growth inhibition zones being a result of antimicrobial activity of silver dressing are presented in Figure 14.

Contrary to the results obtained using the modified disc-diffusion method, the application of BC dressing chemisorbed with OCT led to the formation of larger growth inhibition zones for 12/54 strains compared to silver dressing (*E. faecium* ATCC 19434, *E. coli* ATCC 25922, EC1, EC2, EC4, EC5, *E. cloacae* ATCC 13047, ECL3, ECL4, *A. baumannii* PCM 2740, CA1 and CA3). The rest of the strains were more susceptible to silver activity. BC/OCT inhibited the growth of all tested strains, while the silver dressing was ineffective against two strains. The analysis of the results concerning species has shown that the BC/OCT dressing had a stronger activity only against *E. coli*.

BC dressing chemisorbed with PHMB acted stronger than silver dressing against 27/54 (50%) strains (*S. aureus* ATCC 33591, *S. epidermidis* PCM 2118, SE1, SE4, *E. faecium* ATCC 19434, KP1, KP4, KP5, *E. coli* ATCC 25922, EC1–EC5, *E. cloacae* ATCC 13047, ECL1–ECL5, *A. baumannii* PCM 2740, *C. albicans* ATCC 10321, CA1–CA5). The rest of the strains were more susceptible to silver ions. BC/PHMB inhibited all tested strains’ growth, while the silver dressing was ineffective against two strains. The analysis of the results concerning species has shown that the BC/PHMB dressing displayed a stronger activity against 5 species: *C. albicans*, *E. coli*, *E. cloacae*, *S. epidermidis* and *K. pneumoniae*.

BC dressing chemisorbed with PVP-I acted stronger than the silver dressing against every strain, except *P. aeruginosa* reference and clinical strains (larger growth inhibition zones for 49/54 strains). BC/PVP-I inhibited the growth of all tested strains, while the silver dressing was ineffective against two strains. The analysis of the results concerning species has shown that BC/PHMB dressing had a stronger activity against every tested species except for *P. aeruginosa*.

BC dressing chemisorbed with CHX acted stronger than the silver dressing against every strain, except PA3 and PA4 clinical strains (larger growth inhibition zones for 52/54 strains). BC/CHX inhibited the growth of all tested strains, while the silver dressing was ineffective against two strains. The analysis of the results concerning species has shown that BC/CHX dressing had a stronger activity against every tested species.

BC dressing chemisorbed with EL acted stronger than the silver dressing only against 3/54 strains (SE1, *E. faecium* ATCC 19434, and EC4). BC/EL inhibited the growth of 38/54 strains, while the silver dressing was effective against 52/54 strains. The analysis of the results concerning species has shown that the silver dressing had a stronger activity against every tested species than the BC/EL dressing.

BC/SOH inhibited the growth of only 3/54 strains, while the silver dressing was effective against 52/54 strains. BC dressing chemisorbed with SOH acted weaker than the silver dressing for every tested strain. Analyzing the species’ results, the silver dressing had a stronger activity against every tested species than BC/SOH dressing.

**Figure 15:**
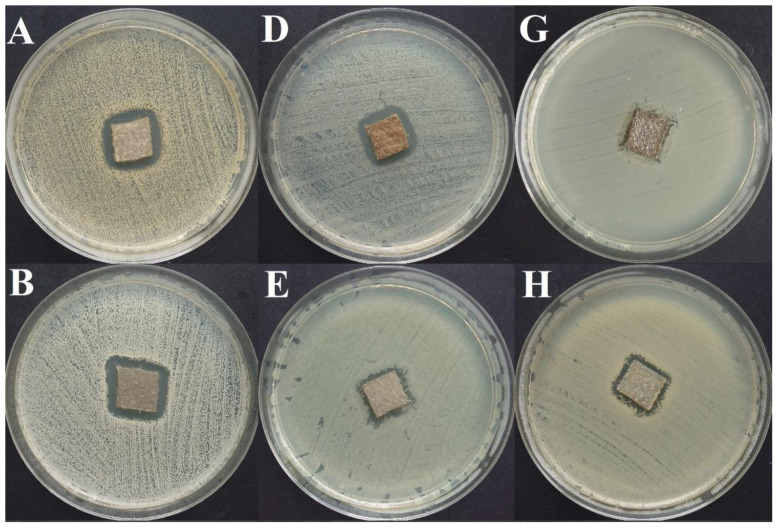

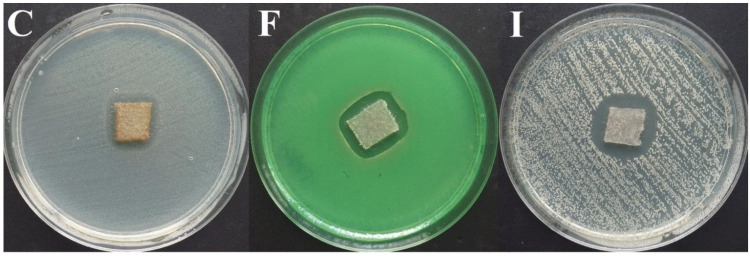
Growth inhibition zones in the modified disc-diffusion method. Aquacel^®^ Ag silver dressing was used, surface area c.a. 196 mm^2^. **A**—*Staphylococcus aureus* ATCC 33591, **B**—*Staphylococcus epidermidis* PCM 2118, **C**—*Enterococcus faecium* ATCC 19434, **D**—*Klebsiella pneumoniae* ATCC 4352, **E**—*Escherichia coli* ATCC 25922, **F**—*Pseudomonas aeruginosa* ATCC 27853, **G**—*Enterobacter cloacae* ATCC 13047, **H**—*Acinetobacter baumannii* PCM 2740, **I**—*Candida albicans* ATCC 10321.

A graphic representation of growth inhibition zone areas caused by the silver dressing compared to BC dressing chemisorbed with antiseptics is presented in Figures S10–S15 of Supplementary Materials. In turn, Table S3 of Supplementary Materials contains all data from the modified disc-diffusion method using the silver dressing. Sample results are shown in Figure 15.

### 2.6. Evaluation of Anti-Biofilm Activity of Chemisorbed Bacterial Cellulose Dressings and Silver Dressing Using the Modified Antibiofilm Dressing’s Activity Measurement (ADAM) Test

**Figure 16:**
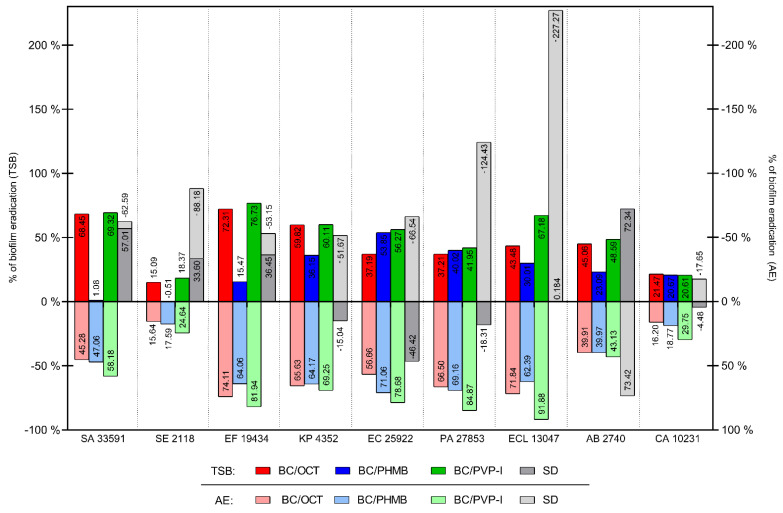
Results of the ADAM test presented an average percentage of metabolically active cells after treatment with BC dressing chemisorbed with antimicrobials or silver dressing. The test was carried out using two kinds of culture media: tryptic soy broth (TSB) and artificial exudate (AE). The left *Y*-axis demonstrates values for TSB and the right *Y*-axis for the AE medium. Tested dressings: BC/OCT—Bacterial cellulose with octenidine dihydrochloride, BC/PHMB—Bacterial cellulose with polyhexanide, BC/PVP-I—Bacterial cellulose with povidone-iodine, SD—Silver dressing. Tested strains: SA 33591—*Staphylococcus aureus* ATCC 33591 SE 2118—*Staphylococcus epidermidis* PCM 2118, EF 19434—*Enterococcus faecium* ATCC 19434 KP 4352—*Klebsiella pneumoniae* ATCC 4352 EC 25922—*Escherichia coli* ATCC 25922 PA 27853—*Pseudomonas aeruginosa* ATCC 27853 ECL 13047—*Enterobacter cloacae* ATCC 13047 AB 2740—*Acinetobacter baumannii* PCM 2740, CA 10231—*Candida albicans* ATCC 10231.

The ADAM test was performed for reference strains. The tested dressings consisted of BC chemisorbed with OCT, PHMB and PVP-I and a silver dressing. Two kinds of culture media were used—TSB and an artificial exudate (AE). For every strain, negative control with BC chemisorbed with normal saline was performed; biofilm grown in such conditions served as biofilm growth control. Figure 16 presents the percentage of eradication of living cells in biofilm grown in both culture media.

The BC dressing chemisorbed with OCT reduced the number of living cells in the biofilm formed by every tested strain in both culture media. The highest reduction in TSB was obtained for *E. faecium* ATCC 19434 (72.31%), *S. aureus* ATCC 33591 (68.45%) and *K. pneumoniae* ATCC 4352 (59.82%). The weakest activity of BC/OCT was observed for *S. epidermidis* PCM 2118 (15.09%) and *C. albicans* ATCC 10231 (21.47%). In AE, the strongest activity of BC/OCT was observed against *E. faecium* ATCC 19434 (74.11%), *E. cloacae* ATCC 13047 (71.84%), *P. aeruginosa* ATCC 27853 (66.50%) and *K. pneumoniae* ATCC 4352 (65.63%). The weakest activity, similar to the results obtained using the TSB culture medium, was observed for *C. albicans* ATCC 10231 (16.20%) and *S. epidermidis* PCM 2118 (15.64%). BC/OCT reduced more metabolically active microbial cells in biofilm in AE than in TSB (50.20% and 44.46% of reduction, respectively).

The BC dressing chemisorbed with PHMB reduced the number of living cells in the biofilm formed by every tested strain in AE and for 8/9 strains in TSB (all strains except *S. epidermidis* PCM 2118). The highest TSB reduction was obtained for *E. coli* ATCC 25922 (53.85%) and *P. aeruginosa* ATCC 33591 (40.02%). The weakest activity of BC with PHMB was observed against *S. aureus* ATCC 33591 (1.08%) and *E. faecium* ATCC 19434 (15.47%). A very slight increase in cell activity was observed for *S. epidermidis* PCM 2118 (0.51% of the increase), but due to its insignificance, the result was interpreted as no antimicrobial activity. In AE, the strongest activity of BC with PHMB was observed for *E. coli* ATCC 25922 (71.06%), *P. aeruginosa* ATCC 33591 (69.16%), *K. pneumoniae* ATCC 4352 (64.17%) and *E. faecium* ATCC 19434 (64.06%). The weakest activity was observed against *C. albicans* ATCC 10231 (18.77%) and *S. epidermidis* PCM 2118 (17.59%). BC with PHMB had about 2-times stronger activity in AE than in TSB (50.47% and 24.42%, respectively). BC dressing chemisorbed with PVP-I reduced the amount of metabolically active cells in biofilm formed by every tested strain in both culture media. The highest reduction in TSB was obtained for *E. faecium* ATCC 19434 (76.73%), *S. aureus* ATCC 33591 (69.32%) and *E. cloacae* ATCC 13047 (67.18%). The weakest activity of BC with PVP-I was observed against *C. albicans* ATCC 10231 (20.61%) and *S. epidermidis* PCM 2118 (18.37%). In AE, the strongest activity of BC with PVP-I was observed against *E. cloacae* ATCC 13047 (91.88%), *P. aeruginosa* ATCC 27853 (84.87%), *E. faecium* ATCC 19434 (81.94%), and *E. coli* ATCC 25922 (78.68%). The weakest activity, similar to the results obtained using the TSB culture medium, was observed against *C. albicans* ATCC 10231 (29.75%) and *S. epidermidis* PCM 2118 (24.64%). A bigger number of metabolically active microbial cells in biofilm was reduced by BC with PVP-I in AE than in TSB (62.48% and 51.01% of reduction, respectively). The silver dressing reduced the amount of metabolically active cells in biofilm formed by 4/9 tested strains in TSB (*S. aureus* ATCC 33591 *S. epidermidis* PCM 2118, *E. faecium* ATCC 19434 and *A. baumannii* PCM 2740) and 1/9 in AE (*A. baumannii* PCM 2740 only). The highest reduction in TSB was obtained for *A. baumannii* PCM 2740 (72.34%) and *S. aureus* ATCC 33591 (57.01%). The weakest activity of the silver dressing was observed against *E. faecium* ATCC 19434 (36.45%) and *S. epidermidis* PCM 2118 (33.60%). For *E. cloacae* ATCC 13047, a very slight reduction was observed (0.184%), but due to its insignificance, the result was interpreted as no antimicrobial activity. For *C. albicans* ATCC 10231 *K. pneumoniae* ATCC 4352 *P. aeruginosa* ATCC 27853 and *E. coli* ATCC 25922, an increase in microbial cell activity was observed (4.48%, 15.04%, 18.31% and 46.42% of active cell increase, respectively). In AE, the activity of the silver dressing was observed against *A. baumannii* PCM 2740 only (73.42% of active cells reduction). For the rest of the strains, there was an increase in the amount of metabolically active cells observed. The highest increase, over 100%, was observed for *E. cloacae* ATCC 13047 (227.27%) and *P. aeruginosa* ATCC 27853 (124.43%) and the lowest for *C. albicans* ATCC 10231 (17.65%). Opposite to BC dressings, the silver dressing had higher antimicrobial activity in TSB (12.82% of metabolically active cells reduction) than in AE (68.67% of metabolically active cells increase).

In TSB, BC dressing with PVP-I had the highest antimicrobial activity against 6/9 species (*S. aureus* ATCC 33591 *E. faecium* ATCC 19434 *P. aeruginosa* ATCC 27853 *K. pneumoniae* ATCC 4352 *E. coli* ATCC 25922 and *E. cloacae* ATCC 13047). Two species were more susceptible to silver dressing (*A. baumannii* PCM 2740 and *S. epidermidis* PCM 2118) and 1 to BC with OCT (*C. albicans* ATCC 10231) than to BC with PVP-I. In AE, BC dressing with PVP-I had the highest antimicrobial activity against 8/9 species (every species except *A. baumannii* PCM 2740). The silver dressing had the highest activity against *A. baumannii* PCM 2740. In both cultural media, the silver dressing results were the most scattered. The results in both media are presented in Figures S16 and S17, and individual stages of the modified ADAM test are presented in Figure S18 of Supplementary Materials.

## 3. Discussion

Chronic wounds are among the most persistent and serious health issues related to the so-called “lifestyle” changes affecting western societies. Chronic wounds, which do not follow the natural healing trajectory, are also at high risk of infection caused by microbial biofilms—settled cellular communities embedded within an external matrix and highly tolerant to therapeutic counter-measures. The complications resulting from microbial biofilm’s presence within chronic wounds often require aggressive treatment (including limb amputation). Moreover, a transition of wound local infection into a systemic one may pose a threat to the patient’s life.

Therefore, a number of approaches designed for chronic wound care have been proposed, including TIME(RS) [15,16,174], biofilm-based wound care strategy (BBWC) [175,176], and WAR [11]. All these strategies emphasize the need for local application of antiseptic substances and dressings chemisorbed with antimicrobials if a local infection appears in the wound.

Bacterial cellulose, which in this research served as a dressing material, is recognized as safe for use in wounds, thanks to its biocompatibility, absence of toxic effects and lack of inflammatory response stimulation [97,177,178,179,180]. Also, BC’s high water capacity, porosity and susceptibility to in situ and ex situ modifications are desirable features in the context of the matter at hand [99,179,181]. An additional advantage of BC, which distinguishes it from other biomaterials, is its chemical purity. No need to remove waxes, pectins and hemicellulose translates into facilitating the production process. Preparing cellulose sheets is easy, and there is no need for highly advanced equipment, which allows reducing production costs. What important, BC can be sterilized at a high temperature without the loss of structure and functionality. Native BC is transparent, does not leave fibers or lint in the wound bed, and adheres to the tissue without the need for any adhesives. These features allow to change dressing painlessly and to control the wound status without removing BC dressing [177,179]. Presently, the vast majority of modern dressings are chemisorbed with various forms of ionic silver. Despite its indisputable effectiveness, the application of the above element also has certain disadvantages, including the risk of material incompatibility (between silver and iodine povidone or chlorine) or such adverse effects as contact dermatitis or allergy [4,17,182,183].

In this line of investigation, we analyzed whether antimicrobials other than silver, specifically antiseptics, applied in the management of biofilm-based wound infections, may also be used as an antimicrobial additive to BC dressings. By making this, we aimed to provide proof of concept for new wound dressings featuring an innovative BC base and antimicrobials of high antibiofilm properties. Therefore, the specific aim of this investigation was to compare the activity of commonly used antiseptic molecules: Octenidine, polyhexanide, povidone-iodine, chlorhexidine, ethacridine lactate, hypochlorous solutions and to evaluate their usefulness as active substances of BC dressings against 54 microbial species, which are frequent etiological agents of wound infections.

Firstly, we have scrutinized the selected microbial strains concerning their ability to form biofilm in vitro. Therefore, we have assessed the metabolic activity of adhered cells and their total biomass. As can be observed in Figure 4, all strains displayed the above ability. Of note, the ratio of biomass to cell number was higher in the case of Gram-negative compared to Gram-positive pathogens, indicating a high share of extracellular slime in the biofilm structure of the former type of microorganisms. It not only stays in line with the results of other research teams [184,185,186] but is also crucial concerning the potential efficacy of antiseptics, bearing in mind the highly protective function of biofilm matrix [187]. In turn, the analyzed yeast strains displayed a ratio of biomass to cell number more resembling that of the analyzed Gram-positive cocci than of the Gram-negative rods.

In the next experimental setting, we have established minimal inhibitory concentrations of antiseptic solutions against the cells of the tested strains (Figure 5, Figure 6 and Figure 7) The rationale behind this analysis was the fact that such antiseptics as OCT, PHMB, CHX and EL act against microbial cells and membranes, while their activity against biofilm matrix is still unrecognized. In turn, the molecules of iodine (released from PVP-I) and chlorine ions (released from NaOCl) are known for their unspecific mode of action against a vast spectrum of organic compounds [188,189]. A correlation can be observed between the results presented in Figure 4 concerning a high share of extracellular slime in Gram-negative pathogens and MIC results (Figure 5 and Figure 6); the aforementioned microorganisms were more resistant to the applied antiseptics than Gram-positive pathogens. On the one hand, the laboratory setting for MIC analysis should prevent biofilm formation (and keep the cells in an un-adhered, planktonic state). Thus, it may be hypothesized that the tested antiseptics simply act better against the peptidoglycan structure of Gram-positive cell walls than against a double membrane of Gram-negative cell walls [33,190]. On the other hand, those familiar with the culturing of Gram-negative bacteria of *Pseudomonadaceae* or *Enterobacterales* group may notice that even the application of extensive shaking may not prevent cells from forming un-adhered aggregates of high-density, suggesting the presence of extracellular slime (which protects the bacteria from antiseptics’ activity). Although such a phenomenon would satisfactorily explain the discussed correlation, it requires an additional line of investigation.

Because wound biofilms may be poly-microbial in nature and are frequently formed by consortia of microorganisms originated from the patient’s skin or alimentary tract, Figure 7 presents the total susceptibility of all analyzed species to individual antiseptics. It should be noted that SOH antiseptic displays no antimicrobial activity within the tested range of concentrations, which stays in line with our earlier results [27] and with the results of other research teams [80,191].

The data presented in Figure 8, Figure 9 and Figure 10 show that the transition of planktonic, sessile cells into adhered biofilm community translates into significantly increased tolerability against antiseptics (cf. Figure 10 vs. Figure 7 and the data provided by [192]). Of note, the MBEC value of PVP-I in this experimental setting was comparable to the MBEC values of PHMB and OCT, while MIC of PVP-I was higher (less favorable) than MIC values of PHMB and OCT). It may be explained by the already-mentioned non-specific mechanism of PVP-I action, which in this particular experimental setting could lead to the destruction of the biofilm matrix in the first step, followed by exposure and death of microbial cells in consequence. In turn, the activity of SOH remained basically beyond the tested range of concentrations. Taking into consideration the lack of SOH’s significant activity against planktonic cells (Figure 7), the increased tolerance of biofilm formed by the cells of the same strains against SOH (Figure 10) is logical and stays in line with the acknowledged protective function of biofilm structure [193]. Importantly, except for the already-mentioned SOH (and EL to a major extent), average MBEC values of OCT, PHMB and PVP-I were approximately at 33% concentration of working solutions of these antiseptics, showing their high antibiofilm properties. Chlorhexidine was the most potent among the tested agents and performed particularly well against *S. aureus* biofilm.

In a subsequent analysis, we have chemisorbed BC with the aforementioned antiseptics (Figure 11). This approach, based on the functionalization of BC and improvement of its applicability concerning the fight against microbial biofilm, has been consistently developed by our team and has been presented in our earlier research [142,150,194,195]. Nevertheless, it is for the first time that we have tested such a high number of species and strains to get, besides other data, an idea concerning inter- and intraspecies variability in the tolerance against antimicrobials released from BC.

The results presented in Figure 12 show that the release of PVP-I from BC translated into the most favorable results concerning the zone of growth inhibition, regardless of the microbial species tested (K-W test, *p* < 0.001) (Figure 12). Such a result explicitly shows the need to apply various experimental settings; one should note that in MIC analysis, PVP-I displayed weak antimicrobial activity (comparing to CHX, PHMB, OCT) and comparable activity to CHX PHMB, OCT in MBEC analysis. In the experimental setting presented in Figure 11, the cells are seeded on a porous, moist structure of an agar polymer. Although the application of this microbiological technique of culturing dates back to the year 1887 [196], one may easily notice that this experimental setting reflects, to some extent, a situation when a wound surface (imitated here by agar polymer) becomes contaminated with microorganisms from the patient’s skin or from the environment. The extrapolation of the results obtained in this experimental setting into clinical practice leads to a conclusion that a BC dressing chemisorbed with PVP-I would protect a wound from microorganisms significantly better than other antiseptics tested in this setting, and conversely, coupling of BC with SOH or EL would not translate into inhibition of microorganisms’ growth within the wound (Figure 12 and Figure 13). In the next stage of our investigation, we have measured the antimicrobial activity of silver released from a commercial, broadly-applied silver dressing (Figure 14 and Figure 15). The silver dressing acted least effective against the *E. coli* and *E. cloacae* strains. It may be due to the widespread use of silver-containing compounds, which led to the emergence of silver-resistant strains of these species, as was observed by Hosny et al. [197]. Interestingly, staphylococcal strains displayed relatively high sensitivity (compared to other species analyzed) to silver cations, although these microbes are also ubiquitous opportunistic pathogens, constantly exposed to silver in a nosocomial environment. Such a phenomenon may be explained by the results presented by Loh et al. [198], who showed that MRSA strains displayed phenotypic sensitivity to silver dressings regardless of the presence of the *sil* gene cluster responsible for resistance to this element.

A comparison of results presented in Figure 12 and Figure 13 vs. Figure 14 shows that the activity of PVP-I or CHX released from BC was significantly higher than the activity of silver cations used in the commercial silver dressing (K-W test, *p* < 0.001). In turn, the antimicrobial activity of the silver dressing was comparable to the activity being a result of PHMB or OCT release from BC. Taking into account the increasing tolerability and resistance of nosocomial pathogens to silver [199], together with the lack of detected resistance mechanism to PVP-I, the results presented in this research indicate that the aforementioned antiseptic can be a promising alternative to be applied either alone or as an antimicrobial additive to BC dressings.

Finally, the results presented in Figure 16 indicate our approach’s validity, including an investigation of a high number of clinical strains. Although the results presented in the aforementioned figure are largely coherent with the results presented in Figure 12, Figure 13, Figure 14 and Figure 15, one may note that the susceptibility of two reference strains of *P. aeruginosa* and *E. cloacae* to silver (Figure 16) is distinctively higher than to PVP-I released from BC (Figure 12, Figure 13, Figure 14 and Figure 15). In fact, the intraspecies deviations in outcomes, recorded between clinical strains, reach a few hundred percent; it explicitly shows how important it is from the point of view of results to screen as big a number of strains as possible to avoid obtaining biased outcomes.

As far as the clinical application of antiseptics chemisorbed in BC or alone is concerned, also criteria other than antimicrobial activity should be carefully considered. The first one is the safety of use. Among the tested antimicrobials, PHMB and OCT are considered safe. There have been rare local skin inflammation incidents, and allergic reactions to OCT were reported. OCT is considered a compound of very low cytotoxicity [42,200,201,202,203]. In addition, PHMB is associated only with rare slight allergic reactions, and it is considered an uncommon allergen. Although there have been a few cases of severe anaphylactic reactions reported, there is no evidence of cytotoxicity, mutagenicity, carcinogenicity, teratogenicity, embryotoxicity or genotoxicity of PHMB [19,42,204,205,206]. Similarly, PVP-I is a rare cause of allergies, and there have only been a few cases of anaphylactic reactions reported worldwide. Nevertheless, the application of PVP-I has some limitations related to the risk of iodine accumulation in the organism. Therefore, PVP-I should not be used longer than seven days, and it should not be used in the elderly, premature babies, pregnant women, and people with thyroid diseases [42,55,206,207,208].

Unlike the aforementioned antiseptics, CHX is relatively often a trigger of allergic and anaphylactic reactions. In high concentrations, CHX is irritating to the skin and eyes. Chlorhexidine toxicity is considered high. In clinical practice, CHX is mostly used in the concentration range from 0.1% to 1%. A big number of research papers have already reported CHX cytotoxicity against eucaryotic cells, even in concentrations below these used in clinical practice [42,201,209,210,211,212]. Ethacridine lactate is considered to be a cytotoxic, genotoxic, cancerogenic and mutagenic compound [17,70,213]. Super-oxidized hypochlorous solutions of low chlorine concentration are considered safe, well-tolerated by tissues and do not delay the wound healing process [80,214,215,216,217].

The third important aspect, which needs to be considered, is the risk of induction of microbial resistance as a result of exposure to the antimicrobial agent. Out of six antimicrobial compounds analyzed in our research, the application of only two of them (PVP-I and SOH) was not related to the risk of resistance emergence. Until recently, also PHMB and OCT have been considered to have no potential to induce microbial resistance [52,218]. However, recent research suggests that long-term use of PHMB can lead to the development of reduced susceptibility displayed by the exposed strains. Renzoni et al. [219] and Landelle et al. [220] observed that single decolonization with PHMB was not sufficient to eradicate the specific MRSA strains. They found a mutation in *mprF* genes, which can be associated with reduced susceptibility to PHMB. In adition, there is a risk of cross-resistance between PHMB and such cell wall-active antibiotics as daptomycin, vancomycin and teicoplanin [219,220]. In 2018 Shepherd et al. observed that the exposure of *Pseudomonas aeruginosa* strains to 0.1% OCT led to increased tolerance to this antiseptic [40]. Also, in 2018 in Germany, *Burkholderia cepacia* growth in aqueous solutions of OCT was observed [41]. The studies mentioned above are just isolated reports and concern only single strains, but in the face of increasing microbial resistance, even slight evidence of resistance emergence should be carefully examined. Resistance to CHX, especially of Gram-negative rods, has been reported for a long time [221,222,223,224,225]. Abundant, worldwide use of CHX has led to a positive selection of resistant strains and to cross-resistance between CHX and other antiseptics or antibiotics, including last-chance drugs [52,224,226,227,228,229]. In turn, Gram-negative rods often develop resistance to EL to such an extent that they can incorporate this compound into their metabolism as nourishment. There have also been reports of resistance to EL developed by *S. aureus* [68,69,230].

The translation of the results of our in vitro study to clinical outcomes should be done carefully. Nevertheless, the data presented here show explicitly that BC’s chemisorption with antimicrobials may be considered a measure to prevent and fight biofilm-based infections of wounds. Another important conclusion is the statement that EL and SOH antiseptics display a very low antimicrobial and antibiofilm activity, regardless of whether released from BC or applied alone. In the era of the renaissance of new hypochlorite formulas, our data, coupled with analogical data provided by Severing et al. [80] and Rembe et al. [191], constitute an important voice in the ongoing discussion on the advisability of the application of SOH antiseptics in highly contaminated wounds. Furthermore, CHX, although in our analysis it displayed high efficiency, should be applied with full awareness of the increasing cross-resistance emergence due to the use of this antimicrobial.

In our study, we have tried to get a glimpse of complex phenomena occurring within various experimental settings and between biomaterial (BC), antiseptic and microbial biofilm. We are aware of the fact that all in vitro biofilm models are not devoid of certain methodological flaws, and we have tried to overcome this disadvantage by using a high number of species, strains and analytical techniques. Among the recurring observations from our study was the high antimicrobial efficacy of PVP-I released from BC; the results obtained for this antiseptic were also more favorable comparing to commercial silver dressing.

Bearing all the above-mentioned limitations of our study in mind, we believe that the data presented in this manuscript will pave the way for other research teams aiming to introduce BC dressings chemisorbed with efficient antiseptics to clinical practice.

## 4. Materials and Methods

### 4.1. Materials

#### 4.1.1. Tested Substances

1. Octenidine dihydrochloride (OCT)—Octenilin^®^ wound irrigation solution (Schülke, Norderstedt, Germany) contains 0.05% of OCT;
2. Polyhexamethylene biguanide hydrochloride (polyhexanide, PHMB)—Prontosan^®^ wound irrigation solution (B. Braun, Melsungen, Hessen, Germany), contains 0.1% of PHMB;
3. Iodine povidone (PVP-I)—Braunol^®^ skin solution liquid (B. Braun, Melsungen, Hessen, Germany), contains 7.5% of PVP-I;
4. Chlorhexidine (CHX)—20% water solution of CHX (Fagron Pharma Cosmetics, Rotterdam, The Netherlands), diluted in water to 0.5% of CHX (concentration recommended for wound irrigation);
5. Ethacridine lactate (EL)—Rivanol^®^ liquid (Prolab, Paterek, Poland), contains 0.1% of EL;
6. Super-oxidized solution with hypochlorites (SOH)—Microdacyn (Kikgel, Ujazd, Poland) contains 0.004% of sodium hypochlorite (NaOCl) and 0.004% of hypochlorous acid (HOCl).

#### 4.1.2. Test Strains

The research was performed on eight species of bacteria and one yeast species, which are common etiological factors of non-healing wound infections and/or cause significant damage within the wound. A total of 54 strains were used for the study, 6 of each species; each group consisting of 1 reference strain and 5 clinical strains:

1. *Staphylococcus aureus* ATCC 33591 and five clinical strains marked as SA1–SA5, (*n* = 6);
2. *Staphylococcus epidermidis* PCM 2118 and five clinical strains marked as SE1–SE5, (*n* = 6);
3. *Enterococcus faecium* ATCC 19434 and five clinical strains marked as EF1–EF5, (*n* = 6);
4. *Escherichia coli* ATCC 25922 and five clinical strains marked as EC1–EC5, (*n* = 6);
5. *Klebsiella pneumoniae* ATCC 4352 and five clinical strains marked as KP1–KP5, (*n* = 6);
6. *Enterobacter cloacae* ATCC 13047 and five clinical strains marked as ECL1–ECL5, (*n* = 6);
7. *Pseudomonas aeruginosa* ATCC 27853 and five clinical strains marked as PA1–PA5, (*n* = 6);
8. *Acinetobacter baumannii* PCM 2740 and five clinical strains marked as AB1–AB5, (*n* = 6);
9. *Candida albicans* ATCC 10231 and five clinical strains marked as CA1–CA5, (*n* = 6).

Three of the tested species were Gram-positive cocci (*S. aureus*, *S. epidermidis*, *E. faecium*), five were Gram-negative rods (*E. coli*, *K. pneumoniae*, *E. cloacae*, *P. aeruginosa*, *A. baumannii*), and one was Gram-positive yeast (*C. albicans*).

All used strains are a part of the Strains Collection of the Department of Pharmaceutical Microbiology and Parasitology, Medical University of Wroclaw, Poland.

#### 4.1.3. Control Materials

0.9% saline solution (Stanlab, Lublin, Poland) was used as a control substance of non-antimicrobial activity. The antimicrobial activity of chemisorbed bacterial cellulose dressings was compared with a silver dressing (Aquacel^®^ Ag, ConvaTec, Berkshire, England) or sterile blotting paper discs (Whatman, Maidstone, England). The Aquacel^®^ Ag silver dressing is a common silver dressing used worldwide, so it represents an appropriate usability control. In turn, sterile 0.9% saline solution and sterile blotting paper are a neutral substance and material, respectively, with no antibacterial/antifungal effect, and they do not affect the growth and reproduction of the tested bacterial and yeast strains.

### 4.2. Methods

#### 4.2.1. Production of Bacterial Cellulose Discs and Chemisorption with Antimicrobial Substances

*Komagataeibacter xylinus* ATCC 53524 culture in Hestrin-Schramm medium (2% glucose (*w/v*; Chempur, Piekary Slaskie, Poland), 0.5% yeast extract (*w/v*; VWR Chemicals, Radnor, PA, USA), 0.5% bactopeptone (*w/v*; VWR Chemicals, Radnor, PA, USA), 0.115% citric acid (*w/v*; POCH, Gliwice, Poland), 0.27% Na_2_HPO_4_ (*w/v*; POCH, Gliwice, Poland), 0.05% MgSO_4_·7H_2_O (*w/v*; POCH, Gliwice, Poland), and 1% ethanol (*v/v*; Stanlab, Lublin Poland) was carried out for 7 days at 28 °C in 24-well microtiter plates (F type, Nest Scientific Biotechnology, Wuxi, China). After incubation time, bacterial cellulose was removed from the medium surface and washed in 0.1 M NaOH (Chempur, Piekary Slaskie, Poland) solution at 80 °C until the BC became white. Then, the BC discs were purified with water to obtain pH = 7 (checked using pH measurement strips, Macherey–Nagel, Düren, Germany) and sterilized in a steam autoclave. The discs ready for further research had a diameter of 16 mm. BC discs were placed into 24-well plates (F type, Nest Scientific Biotechnology, Wuxi, China), and 1 mL of antimicrobial substances were added. The samples were incubated overnight at 4 °C.

#### 4.2.2. Evaluation of Test Strains Resistance Mechanisms

All strains (*n* = 54) were used in this test.

The methodology for determining resistance mechanisms is derived from The European Committee on Antimicrobial Susceptibility Testing (EUCAST) 2020/2021 guidelines. In the disc-diffusion method, the antibiotic discs (Oxoid Thermo Fisher Scientific, Hampshire, United Kingdom), 0.5 M ethylenediaminetetraacetic acid solution (EDTA, Merck, Darmstadt, Germany), 15 mg/mL solution of phenylboronic acid (Merck, Darmstadt, Germany) and Mueller–Hinton agar (Biomaxima, Lublin, Poland) were used. In the MIC method, the Mueller–Hinton broth (Biomaxima, Lublin, Poland) and vancomycin (Vancomycin MIP-500, MIP Pharma, Gdansk, Poland) were used [127,128].

To determine whether the test strains have developed resistance mechanisms, the disc-diffusion method and minimal inhibitory concentration test were performed; for all Gram-negative rods, the presence of β-lactamases (*Klebsiella pneumoniae* carbapenemase, KPC; metallo-β-lactamase, MBL; extended-spectrum β-lactamases, ESBL) was also checked. Moreover, for *E. coli*, *K. pneumoniae* and *E. cloacae*, the presence of D-class carbapenemase OXA-48 was checked. *S. aureus* and *S. epidermidis* strains were tested for the presence of methicillin resistance (methicillin-resistant *S. aureus*, MRSA and methicillin-resistant coagulase-negative *Staphylococci*, MRCNS) and macrolides, lincosamides and streptogramins B resistance (MLSB). *S. aureus* and *E. faecium* strains were tested for vancomycin resistance (vancomycin-resistant *S. aureus*, VRSA and vancomycin-resistant *Enterococci*, VRE). Additionally, *E. faecium* strains were also tested for the presence of high-level aminoglycoside resistance (HLAR). All the investigated resistance mechanisms are commonly determined during routine microbiological diagnostics. There were no resistance mechanisms checked for *C. albicans* strains because, in routine microbiological diagnostics, there are no guidelines for their determination.

#### 4.2.3. Comparison of the Amount of Formed Biofilm and Metabolic Activity of Bacteria/Yeast Cells in Biofilm Structure

All strains (*n* = 54) were used in this test.

To determine the amount of biofilm formed by different species and strains, crystal violet staining was performed. The metabolic activity of bacteria/yeast cells was determined by using 2,3,5-triphenyl tetrazolium chloride (TTC, PanReac AppliChem, Darmstadt, Germany). The 24 h bacterial/yeast broth cultures in tryptic-soy broth culture medium (TSB, Biomaxima, Lublin, Poland) were prepared and diluted in sterile 0.9% saline (Stanlab, Lublin, Poland) to 0.5 McF (McFarland turbidity scale) using a densitometer (DensiLaMeter II, Erba Lachema, Brno, Czech Republic). The suspensions were diluted 1000-times to obtain a cell density of ca. 105 CFU/mL (colony-forming unit, CFU). The wells of a 96-well microtitration plate (F type, Nest Scientific Biotechnology, Wuxi, China) were filled with 200 µL of diluted suspensions in 12 repetitions, separately for both tests. The samples were incubated at 37 °C for 24 h in static conditions to form biofilm. Then, the culture medium over the biofilm was removed (8.5 mL/min, Ismatec Reglo Digital, Ismatec, Wertheim, Germany). Two hundred µL of 20% water solution of crystal violet (CV, Aqua-Med, Lodz, Poland) or 200 µL of a 0.1% solution of TTC (PanReac AppliChem, Darmstadt, Germany) in TSB (Biomaxima, Lublin, Poland) was added. A 0.001% solution of resazurin (Acros Organics, Geel, Belgium) in TSB (Biomaxima, Lublin, Poland) was added instead of TTC (PanReac AppliChem, Darmstadt, Germany) to the samples containing *Candia albicans* biofilms due to a very poor ability of *Candida* *albicans* to metabolize TTC (PanReac AppliChem, Darmstadt, Germany). The samples with TTC (PanReac AppliChem, Darmstadt, Germany) and resazurin (Acros Organics, Geel, Belgium) were incubated for 2 h at 37 °C and the samples with CV (Aqua-Med, Lodz, Poland) for 10 min at room temp. CV (Aqua-Med, Lodz, Poland) and TTC (PanReac AppliChem, Darmstadt, Germany) was removed from above the biofilms. The samples stained with CV (Aqua-Med, Lodz, Poland) were rinsed twice with 0.9% saline (Stanlab, Lublin, Poland) to remove the dye from the polystyrene. The next step was adding a solvent—200 µL of 100% methanol (Chempur, Piekary Slaskie, Poland) for TTC (PanReac AppliChem, Darmstadt, Germany) and 200 µL of 30% water solution of acetic acid (Chempur, Piekary Slaskie, Poland) for CV-stained biofilms (Aqua-Med, Lodz, Poland). The samples with solvents were incubated for 15 min with shaking at 400 rpm (PSU 2-T, Biosan, Riga, Latvia). In the case of resazurin (Acros Organics, Geel, Belgium), the discoloration step was omitted because the dye transfers from the cells to the solution. One hundred µL of each sample was transported to a new 96-well plate (F type, Nest Scientific Biotechnology, Wuxi, China), and spectrophotometric measurements were performed (Multiscan Go, Thermo Fisher Scientific, Waltham, MA, USA)—for TTC (PanReac AppliChem, Darmstadt, Germany) at a wavelength of 490 nm, for resazurin (Acros Organics, Geel, Belgium) at 570 nm and for CV (Aqua-Med, Lodz, Poland) at 550 nm.

#### 4.2.4. Evaluation of Minimal Inhibitory Concentration (MIC) and Minimal Biofilm Eradication Concentration (MBEC) of Test Substances

All test substances (*n* = 6) and strains (*n* = 54) were used in MIC and MBEC tests.

The MICs of antiseptics were determined by the broth microdilution method. Each substance was tested in a concentration range of 50–0.098% of its working solution. If the MIC value was not determined within this concentration range, the dilution series was extended to 0.0015% of the working solution. Dilutions were prepared in tryptic soy broth (Biomaxima, Lublin, Poland). Every substance was tested in triplicate, and for every replicate, the bacteria/yeast growth control (without tested substances) and medium sterility control (the culture medium only) were done. The 24 h bacterial/yeast broth cultures in TSB (Biomaxima, Lublin, Poland) were prepared and diluted in sterile 0.9% saline (Stanlab, Lublin, Poland) to 0.5 McF using a densitometer (DensiLaMeter II, Erba Lachema, Brno, Czech Republic). The suspensions were diluted 1000 times to obtain a cell density of ca. 105 CFU/mL. Bacterial/yeast suspensions were added to dilution series of antiseptics in a volume ratio of 1:1 to obtain the test concentration range mentioned above. The test was carried out in 96-well sterile microtiter plates (F type, Nest Scientific Biotechnology, Wuxi, China). The samples were incubated overnight at 37 °C with shaking at 400 rpm (PSU 2-T, Biosan, Riga, Latvia). Before and after incubation, spectrophotometric measurements (Multiscan Go, Thermo Fisher Scientific, Waltham, MA, USA) at a wavelength of 580 nm were done to determine the minimal inhibitory concentration of the test substances. After the second measurement, 20 µL of 1.0% solution of TTC (PanReac AppliChem, Darmstadt, Germany) in TSB (Biomaxima, Lublin, Poland) was added to each well to visualize metabolically active cells. To the samples containing *Candia albicans* biofilms, 20 µL of 0.02% solution of resazurin (Acros Organics, Geel, Belgium) in TSB (Biomaxima, Lublin, Poland) instead of TTC (PanReac AppliChem, Darmstadt, Germany) was added due to the very poor ability of *Candida albicans* to metabolize TTC (PanReac AppliChem, Darmstadt, Germany). The samples were incubated for 2 h at 37 °C with shaking 400 at rpm (PSU 2-T, Biosan, Riga, Latvia), and MIC values were determined based on the change in medium color from yellow to red (TTC, PanReac AppliChem, Darmstadt, Germany) or from blue to pink (resazurin, Acros Organics, Geel, Belgium).

MBECs of antiseptics were also determined by the broth microdilution method. Every substance was tested in triplicate, and for every replicate, the bacteria/yeast growth control (without the test substances) and medium sterility control (the culture medium only) were done. The 24 h bacterial/yeast broth cultures in TSB (Biomaxima, Lublin, Poland) were prepared and diluted in sterile 0.9% saline (Stanlab, Lublin, Poland) to 0.5 McF. The suspensions were diluted 1000 times to obtain a cell density of about 105 CFU/mL (DensiLaMeter II, Erba Lachema, Brno, Czech Republic). Two hundred µL of bacterial/yeast suspensions were added to the wells of 96-well sterile microtiter plate (F type, Nest Scientific Biotechnology, Wuxi, China) and incubated at 37 °C for 24 h in static conditions to form biofilm. After incubation time, the culture medium from over the biofilms was removed. Serial dilutions of antiseptics were prepared by adding the TSB (Biomaxima, Lublin, Poland) medium to the test substances. The obtained concentration range was from 100% to 0.2% of the test substances working solutions. The dilution series was added to the wells with the formed biofilm and incubated overnight at 37 °C. Next, the fluid was removed, and 200 µL 0.1% solution of TTC (PanReac AppliChem, Darmstadt, Germany) in TSB (Biomaxima, Lublin, Poland) was added. Two hundred µL of 0.001% solution of resazurin (Acros Organics, Geel, Belgium) in TSB (Biomaxima, Lublin, Poland) instead of TTC (PanReac AppliChem, Darmstadt, Germany) was added to the samples containing *Candia albicans* biofilms. After 2 h of incubation at 37 °C, MBECs were determined based on culture medium color change from yellow to red (TTC) or blue to pink (resazurin).

#### 4.2.5. Evaluation of Antimicrobials’ Activity Using Disk-Diffusion Method

All the test substances (*n* = 6) and strains (*n* = 54) were used for the disc-diffusion test.

To evaluate the antimicrobial activity of the test substances, the disc diffusion method was used. Sterile blotting paper discs (diameter 16 mm, Whatman, Maidstone, England) were dipped in 0.5 mL of the test substances for 10 min. The 24 h bacterial/yeast broth cultures in TSB (Biomaxima, Lublin, Poland) were prepared and diluted in sterile 0.9% saline (Stanlab, Lublin, Poland) to 0.5 McF (DensiLaMeter II, Erba Lachema, Brno, Czech Republic). The microorganisms were spread evenly throughout the Mueller–Hinton agar plates (Biomaxima, Lublin, Poland) and soaked blotting paper discs were put on the plates. The cultures were incubated overnight at 37 °C, and then the growth inhibition zone diameters were measured. The areas of the zones were calculated, and the area of the paper disc was subtracted from them.

#### 4.2.6. Evaluation of Antimicrobial Activity of Chemisorbed Bacterial Cellulose Dressings Using Modified Disk-Diffusion Method

All the test substances (*n* = 6) and strains (*n* = 54) were used in the disc-diffusion test.

The 24 h bacterial/yeast broth cultures in TSB (Biomaxima, Lublin, Poland) were prepared, diluted in sterile 0.9% saline (Stanlab, Lublin, Poland) to 0.5 McF (DensiLaMeter II, Erba Lachema, Brno, Czech Republic) and spread evenly throughout Mueller–Hinton agar plates (Biomaxima, Lublin, Poland). The previously prepared chemisorbed bacterial cellulose discs were put on the plates. The cultures were incubated overnight at 37 °C. Then the growth inhibition zones diameters were measured. The areas of the zones were calculated, and the area of BC disc was subtracted from them.

#### 4.2.7. Evaluation of Silver Dressings’ Activity Using the Modified Disk Diffusion Method

All the test strains (*n* = 54) were used for the disc-diffusion test.

A silver dressing was used as comparative material for bacterial cellulose dressings. The Aquacel^®^ Ag (ConvaTec, Berkshire, England) dressing was cut into 14 mm side squares. The microorganisms were spread evenly throughout the Mueller–Hinton agar plates (Biomaxima, Lublin, Poland) and pieces of silver dressings were put on the plates. The cultures were incubated overnight at 37 °C, and then the growth inhibition zones sides were measured. The areas of the zones were calculated, and the areas of silver dressing pieces were subtracted from them.

#### 4.2.8. Evaluation of Anti-Biofilm Activity of Chemisorbed Bacterial Cellulose Dressings Using the Modified Antibiofilm Dressing’s Activity Measurement (ADAM) Test

The modified ADAM test was performed using 3 of the test substances: octenidine dihydrochloride (Octenilin^®^ Schülke, Norderstedt, Germany), polyhexanide (Prontosan ^®^, B. Braun, Melsungen, Hessen, Germany) and iodine povidone (Braunol^®^, B. Braun, Melsungen, Hessen, Germany). Sterile 0.9% NaCl solution (Stanlab, Lublin, Poland) was used as a negative control. The test was carried out for reference strains only (*n* = 9).

For the purposes of the modified ADAM test [129,130], mini agar-based reaction tubes and agar discs were prepared. The 24-well plates (F type, Nest Scientific Biotechnology, Wuxi, China) were filled with microbiological agar (VWR Chemicals, Radnor, PA, USA) to 4/5 of their height. Following congealing of the agar, the wells were cut in the middle of the agar using a cork-borer (well diameter 5.0 mm). Using the same size cork-borer, the agar discs were cut from the 5.0 mm thick agar plates. Two kinds of culture media were used for the ADAM test: TSB (Biomaxima, Lublin, Poland) as a basic culture medium and a mixture imitating an artificial exudate (AE). AE was prepared by mixing 1% of mucin (Merck, Darmstadt, Germany), 1% of bovine serum albumin (VWR Chemicals, Radnor, PA, USA), 10% of fetal bovine serum (Biowest, France) and 88% of RPMI 1640 cell culture medium (Biowest, Nuaille, France). The AE was filtered through 0.21 micron pore size filters (Filtropur S plus, Sarstedt, Nümbrecht, Germany) to obtain sterility. The 24 h bacterial/yeast broth cultures in TSB (Biomaxima, Lublin, Poland) were prepared and diluted in sterile 0.9% saline (Stanlab, Lublin, Poland) to 0.5 McF (DensiLaMeter II, Erba Lachema, Brno, Czech Republic). The suspensions were diluted 105 times in TSB (Biomaxima, Lublin, Poland) and AE to obtain a cell density of about 103 CFU/mL. The previously prepared agar discs were placed into 24-well plates (F type, Nest Scientific Biotechnology, Wuxi, China), and 2.0 mL of bacterial/yeast suspensions were added, with suspensions in different media added to separate plates. The samples were incubated at 37 °C for 24 h in static conditions to form biofilm on the agar discs. The BC discs were placed in 24-well plates (F type, Nest Scientific Biotechnology, Wuxi, China) and 1.0 mL of the substances (OCT (Octenilin^®^ Schülke, Norderstedt, Germany), PHMB (Prontosan^®^, B. Braun, Melsungen, Hessen, Germany), PVP-I (Braunol^®^, B. Braun, Melsungen, Hessen, Germany) and NaCl (Stanlab, Lublin, Poland) was added. The BC discs were incubated overnight at 4 °C. After incubation time, the agar discs with biofilms on them were gently pulled out of the 24-well plates (F type, Nest Scientific Biotechnology, Wuxi, China) and placed into the agar wells prepared in advance. The space above the biofilms was filled with culture media—one series with TSB (Biomaxima, Lublin, Poland), the second with AE. The chemisorbed bacterial cellulose discs were placed on top of the agar tube (6 repetitions of 1 substance per species per medium). The samples were incubated at 37 °C for 24 h under static conditions. Then the BC discs were removed, and the agar discs were gently moved to new 24-well plates (F type, Nest Scientific Biotechnology, Wuxi, China). Two mL of 0.1% solution of TTC (PanReac AppliChem, Darmstadt, Germany) in TSB (Biomaxima, Lublin, Poland) was added to the wells and incubated for 4 h at 37 °C to visualize metabolically active cells. A 0.001% solution of resazurin (Acros Organics, Geel, Belgium) in TSB (Biomaxima, Lublin, Poland) instead of TTC (PanReac AppliChem, Darmstadt, Germany) was added to the samples containing *Candia albicans* biofilms due to the very poor ability of *Candida albicans* to metabolize TTC (PanReac AppliChem, Darmstadt, Germany). Incubation time and conditions were the same as in the method with TTC (PanReac AppliChem, Darmstadt, Germany). Then, the fluid over the agar discs was removed, the discs were moved to new 24-well plates (F type, Nest Scientific Biotechnology, Wuxi, China), and 2.0 mL of methanol (Chempur, Piekary Slaskie, Poland) was added. The plates were incubated for 15 min at 37 °C with shaking at 300 rpm (PSU 2-T, Biosan, Riga, Latvia) to release the color to the solution. Two hundred µL of the obtained solutions were moved from each well to 96-well plates (F type, Nest Scientific Biotechnology, Wuxi, China) in 4 repetitions and measured spectrophotometrically (Multiscan Go, Thermo Fisher Scientific, Waltham, MA, USA) at 490 nm for TTC (PanReac AppliChem, Darmstadt, Germany) and at 570 nm for resazurin (Acros Organics, Geel, Belgium).

#### 4.2.9. Evaluation of Anti-Biofilm Activity of Silver Dressing Using the Modified Antibiofilm Dressing’s Activity Measurement (ADAM) Test

This test was performed for reference strains (*n* = 9). The silver dressing (Aquacel^®^ Ag, ConvaTec, Berkshire, England) was used as comparative material for bacterial cellulose dressings. The preparation and carrying out of the modified ADAM test were the same as described in the previous subsection. Instead of chemisorbed BC discs, square pieces of silver dressings with a side of 14 mm were used. BC discs saturated with sterile 0.9% NaCl solution (Stanlab, Lublin, Poland) were used as a controlled setting of the microorganisms’ growth.

#### 4.2.10. Statistical Analysis

Calculations were performed using the GraphPad Prism version 7 software (GraphPad Co., San Diego, CA, USA). The normality of distribution was assessed using the D’Agostino–Pearson’s omnibus test. Because all values were non-normally distributed, the Kruskal–Wallis test with post hoc Dunnett analysis was applied. The results of statistical analyses were considered significant if they produced *p*-values < 0.001.

## 5. Conclusions

- By chemisorption with various classes of antiseptics, BC can be functionalized into dressing displaying antimicrobial and antibiofilm properties;
- PVP-I released from BC displayed the highest antibiofilm activity among the tested antiseptics, while SOH and EL were of little or no usability in this aspect;
- BC dressings chemisorbed with PVP-I were more effective against biofilms than commercially applied silver dressings;
- The antimicrobial compound applied as an additive to the dressing should be selected not only based on its antimicrobial activity but also concerning its safety of use and the potential to induce microbial resistance.

### Limitations of This Study

All the tests in our research were performed in vitro and should be considered as preliminary studies. We used commercially available antimicrobial products and compared their activity against eight species of bacteria and one species of yeast, six strains per species. Confirmation of the superior antimicrobial efficacy of one of the tested compounds would require a further increase in the number of test strains and the performance of clinical trials. We have compared the antimicrobial activity of BC dressings chemisorbed with antimicrobials against only one type of silver dressing (Aquacel^®^ Ag). There are other silver dressings with a higher content of this element. The rationale behind our decision was to use a commercial product of high popularity among clinical practitioners, and Aquacel^®^ Ag definitely meets this criterion. Nevertheless, the application of other commercially available silver dressings could produce different outcomes.

## Data Availability

All data are provided in the main body of the manuscript and Supplementary Data.

## Supplementary Materials

The following are available online at https://www.mdpi.com/article/10.3390/ijms22083996/s1, Figure S1. Areas of growth inhibition zones for *Staphylococcus aureus* strains, Figure S2. Areas of growth inhibition zones for *Staphylococcus epidermidis* strains, Figure S3. Areas of growth inhibition zones for *Enterococcus faecium* strains, Figure S4. Areas of growth inhibition zones for *Klebsiella pneumoniae* strains, Figure S5. Areas of growth inhibition zones for *Escherichia coli* strains, Figure S6. Areas of growth inhibition zones for *Pseudomonas aeruginosa* strains, Figure S7. Areas of growth inhibition zones for *Enterobacter cloacae* strains, Figure S8. Areas of growth inhibition zones for *Acinetobacter baumannii* strains, Figure S9. Areas of growth inhibition zones for *Candida albicans* strains, Figure S10. Graphic representation of average growth inhibition zones areas (mm^2^) caused by silver dressing compared to o BC dressing chemisorbed with octenidine (BC with OCT), Figure S11. Graphic representation of average growth inhibition zones areas caused by silver dressing compared to BC dressing chemisorbed with polyhexanide (BC with PHMB), Figure S12. Graphic representation of average growth inhibition zones areas caused by silver dressing compared to BC dressing chemisorbed with povidone-iodine (BC with PVP-I), Figure S13. Graphic representation of average growth inhibition zones areas caused by silver dressing compared to BC dressing chemisorbed with chlorhexidine (BC with CHX), Figure S14. Graphic representation of average growth inhibition zones areas caused by silver dressing compared to BC dressing chemisorbed with ethacridine lactate (BC with EL), Figure S15. Graphic representation of average growth inhibition zones areas caused by silver dressing compared to BC dressing chemisorbed with super-oxidized hypochlorous solution (BC with SOH), Figure S16. The results of the ADAM test presented as the average percentage of metabolically active cells in biofilm eradication in tryptic-soy broth (TSB) culture medium, Figure S17. The results of the ADAM test presented as the average percentage of metabolically active cells in biofilm eradication in an artificial exudate (AE) culture medium, Figure S18. Stages of the modified ADAM test, Table S1. Resistance mechanisms of the test strains, Table S2. Areas of growth inhibition zones around BC dressings (BC) chemisorbed with the test antimicrobial compounds, Table S3. Average growth inhibition zones (mm^2^) obtained in the modified disc-diffusion method using a silver dressing.
