# Supplementary material for: In Vitro Efficacy of Bacterial Cellulose Dressings Chemisorbed with Antiseptics against Biofilm Formed by Pathogens Isolated from Chronic Wounds
Source: Int J Mol Sci. 2021 Apr 13;22(8):3996. doi: 10.3390/ijms22083996 (PMC8069587; doi:10.3390/ijms22083996)
Supplement: Supplementary file 1 [file ijms-22-03996-s001.pdf]

International Journal of *Molecular Sciences*

MDPI

Supplementary Materials

In Vitro Efficacy of Bacterial Cellulose Dressings Chemisorbed with Antiseptics Against Biofilm Formed by Pathogens Isolated from Chronic Wounds

Karolina Dydak [1], Adam Junka [1,*], Agata Dydak [2], Malwina Brożyna [1], Justyna Paleczny [1], Karol Fijalkowski [3], Grzegorz Kubielas [4], Olga Aniołek [5] and Marzenna Bartoszewicz [1]

[1] Department of Pharmaceutical Microbiology and Parasitology, Medical University of Wroclaw, 50-556 Wroclaw, Poland; karolina.dydak@umed.wroc.pl (K.D.); malwina.brozyna@student.umed.wroc.pl (M.B.); justyna.paleczny@student.umed.wroc.pl (J.P.); marzenna.bartoszewicz@umed.wroc.pl (M.B.)
[2] Faculty of Biological Sciences, University of Wroclaw, 51-148 Wroclaw, Poland; adam.junka@umed.wroc.pl
[3] Department of Microbiology and Biotechnology, Faculty of Biotechnology and Animal Husbandry, West Pomeranian University of Technology, Szczecin, Piastow 45, 70-311 Szczecin, Poland; kfijalkowski@zut.edu.pl
[4] Faculty of Health Sciences, Wroclaw Medical University, 50-996 Wroclaw, Poland; grzegorz.kubielas@umed.wroc.pl
[5] Faculty of Medicine, Lazarski University, 02-662 Warsaw, Poland; olga.aniolek@lazarski.pl
* Correspondence: adam.junka@umed.wroc.pl; Tel.: +48-889229341

Citation: Dydak, K.; Junka, A.; Dydak, A.; Brożyna, M.; Paleczny, J.; Fijalkowski, K.; Kubielas, G.; Aniołek, O.; Bartoszewicz, M. In Vitro Efficacy of Bacterial Cellulose Dressings Chemisorbed with Antiseptics Against Biofilm Formed by Pathogens Isolated from Chronic Wounds. *Int. J. Mol. Sci.* **2021**, *22*, x. https://doi.org/10.3390/xxxxx

Academic Editors: Giovanna Batoni; Giuseppantonio Maisetta and Semih Esin

Received: 30 March 2021
Accepted: 12 April 2021
Published: 13 April 2021

Publisher's Note: MDPI stays neutral with regard to jurisdictional claims in published maps and institutional affiliations.

Copyright: © 2021 by the authors. Licensee MDPI, Basel, Switzerland. This article is an open access article distributed under the terms and conditions of the Creative Commons Attribution (CC BY) license (http://creativecommons.org/licenses/by/4.0/).

Abstract: Local administration of antiseptics is required to prevent and fight against biofilm-based infections of chronic wounds. One of the methods used for delivering antiseptics to infected wounds is the application of dressings chemisorbed with antimicrobials. Dressings made of bacterial cellulose (BC) display several features, making them suitable for such a purpose. This work aimed to compare the activity of commonly used antiseptic molecules: octenidine, polyhexanide, povidone-iodine, chlorhexidine, ethacridine lactate, and hypochlorous solutions and to evaluate their usefulness as active substances of BC dressings against 48 bacterial strains (8 species) and 6 yeast strains (1 species). A silver dressing was applied as a control material of proven antimicrobial activity. The methodology applied included the assessment of minimal inhibitory concentrations (MIC) and minimal biofilm eradication concentration (MBEC), the modified disc-diffusion method, and the modified antibiofilm dressing activity measurement (A.D.A.M.) method. While in 96-well plate-based methods (MIC and MBEC assessment), the highest antimicrobial activity was recorded for chlorhexidine, in the modified disc-diffusion method and in the modified A.D.A.M test, povidone-iodine performed the best. In an in vitro setting simulating chronic wound conditions, BC dressings chemisorbed with polyhexanide, octenidine, or povidone-iodine displayed a similar or even higher antibiofilm activity than the control dressing containing silver molecules. If translated into clinical conditions, the obtained results suggest high applicability of BC dressings chemisorbed with antiseptics to eradicate biofilm from chronic wounds.

Keywords: bacterial cellulose; dressing; antiseptics; chronic wounds

Table S1. **Resistance mechanisms of tested strains.** KPC – *K. pneumoniae* carbapenemase; MBL – metallo-β-lactamase; ESBL – extended spectrum of β-lactamases; OXA-48 – class D carbapenemases; MRSA/MRCNS – methicillin resistant *S. aureus*/methicillin resistant coagulase negative *Staphylococci*; MLS_B – macrolides, lincosamides and streptogramin B resistance, con + - constitutive MLS_B, ind + - inductive MLS_B, MS_B - retained susceptibility to lincosamides; VRSA/VRE – vancomycin resistant *S. aureus*/vancomycin resistant *Enterococci*; HLAR – high level of aminoglycosides resistance. Tested strains: SA – *Staphylococcus aureus*, SE – *Staphylococcus epidermidis*, EF – *Enterococcus faecium*, KP – *Klebsiella pneumoniae*, EC – *Escherichia coli*, PA – *Pseudomonas aeruginosa*, ECL – *Enterobacter cloacae*, AB – *Acinetobacter baumannii*, CA – *Candida albicans*. Green – lack of resistance mechanism, red – resistance mechanism occurs, grey – this resistance mechanism is not relevant for the strain.

	KPC	MBL	ESBL	OXA-48	MRSA/MRCNS	MLSB	VRSA/VRE	HLAR
**SA 33591**					+	-	0,125 µg/ml	
**SA 1**					+	con +	0,0625 µg/ml	
**SA 2**					+	con +	0,5 µg/ml	
**SA 3**					+	ind +	0,25 µg/ml	
**SA 4**					+	con +	0,125 µg/ml	
**SA 5**					+	con +	0,25 µg/ml	
**SE 2118**					-	-		
**SE 1**					-	-		
**SE 2**					-	-		
**SE 3**					-	-		
**SE 4**					-	MSB +		
**SE 5**					-	-		
**EF 19434**							0,25 ug/ml	-
**EF 1**							128 µg/ml	+
**EF 2**							250 µg/ml	+
**EF 3**							250 µg/ml	+
**EF 4**							512 µg/ml	+
**EF 5**							128 µg/ml	+
**KP 4352**	-	-	-	-				
**KP 1**	-	-	+	-				
**KP 2**	-	+	+	+				
**KP 3**	+	+	+	+				
**KP 4**	-	-	+	-				
**KP 5**	-	-	+	-				
**EC 25922**	-	-	-	-				
**EC 1**	-	-	-	-				
**EC 2**	-	-	-	-				
**EC 3**	-	-	+	-				
**EC 4**	-	-	-	-				
**EC 5**	-	-	+	-				
**PA 27853**	-	+	-					
**PA 1**	-	-	-					
**PA 2**	-	+	-					
**PA 3**	-	+	-					
**PA 4**	-	+	-					
**PA 5**	-	+	-					
**ECL 13047**	-	-	-	-				
**ECL 1**	-	-	+	+				
**ECL 2**	-	+	+	-				
**ECL 3**	-	-	+	-				
**ECL 4**	-	+	+	-				
**ECL 5**	-	+	-	+				
**AB 2740**	-	-	-					
**AB 1**	-	-	-					
**AB 2**	-	-	-					
**AB 3**	-	-	-					
**AB 4**	-	-	-					
**AB 5**	-	-	-					

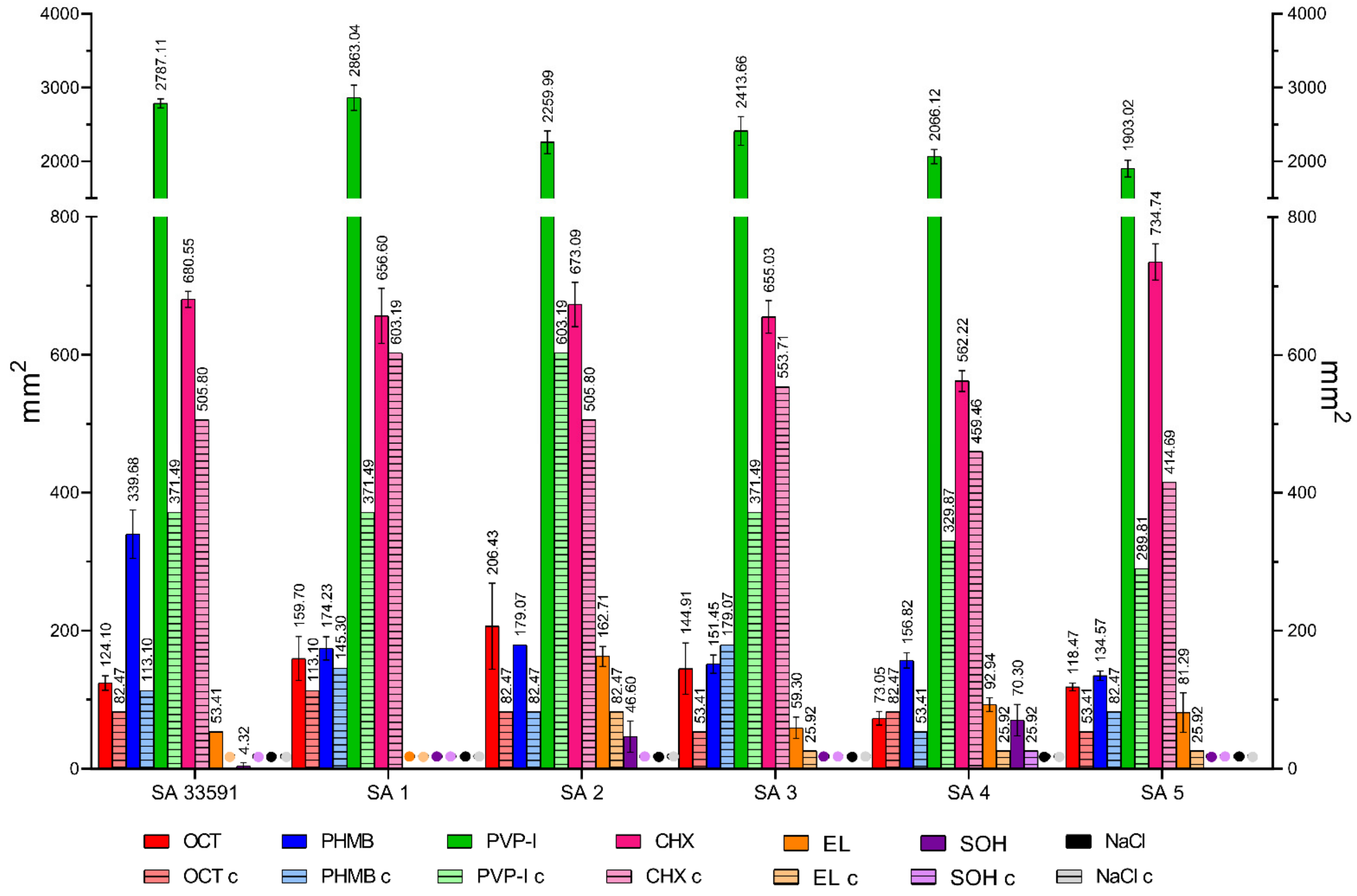

Figure S1. **Areas of growth inhibition zones for *Staphylococcus aureus* strains**. Coloured bars indicate average growth inhibition zones areas caused by bacterial cellulose (BC) dressings chemisorbed with tested compounds (OCT – octenidine dihydrochloride, PHMB – polyhexanide, PVP-I – povidone iodine, CHX – chlorhexidine, EL – ethacridine lactate, SOH – super-oxidized hypochlorites solution, NaCl – sodium chloride as a negative control); striped bars indicate areas of growth inhibition zones caused by blotting paper soaked with tested compounds (OCT c – control of OCT activity, PHMB c – control of PHMB activity, PVP-I c – control of PVP-I activity, CHX c – control of CHX activity, EL c – control of EL activity, SOH c – control of SOH activity, NaCl c – control of NaCl activity). Dots point ineffective BC/blotting paper dressings. Growth inhibition zones areas exclude BC/blotting paper dressings surface areas.

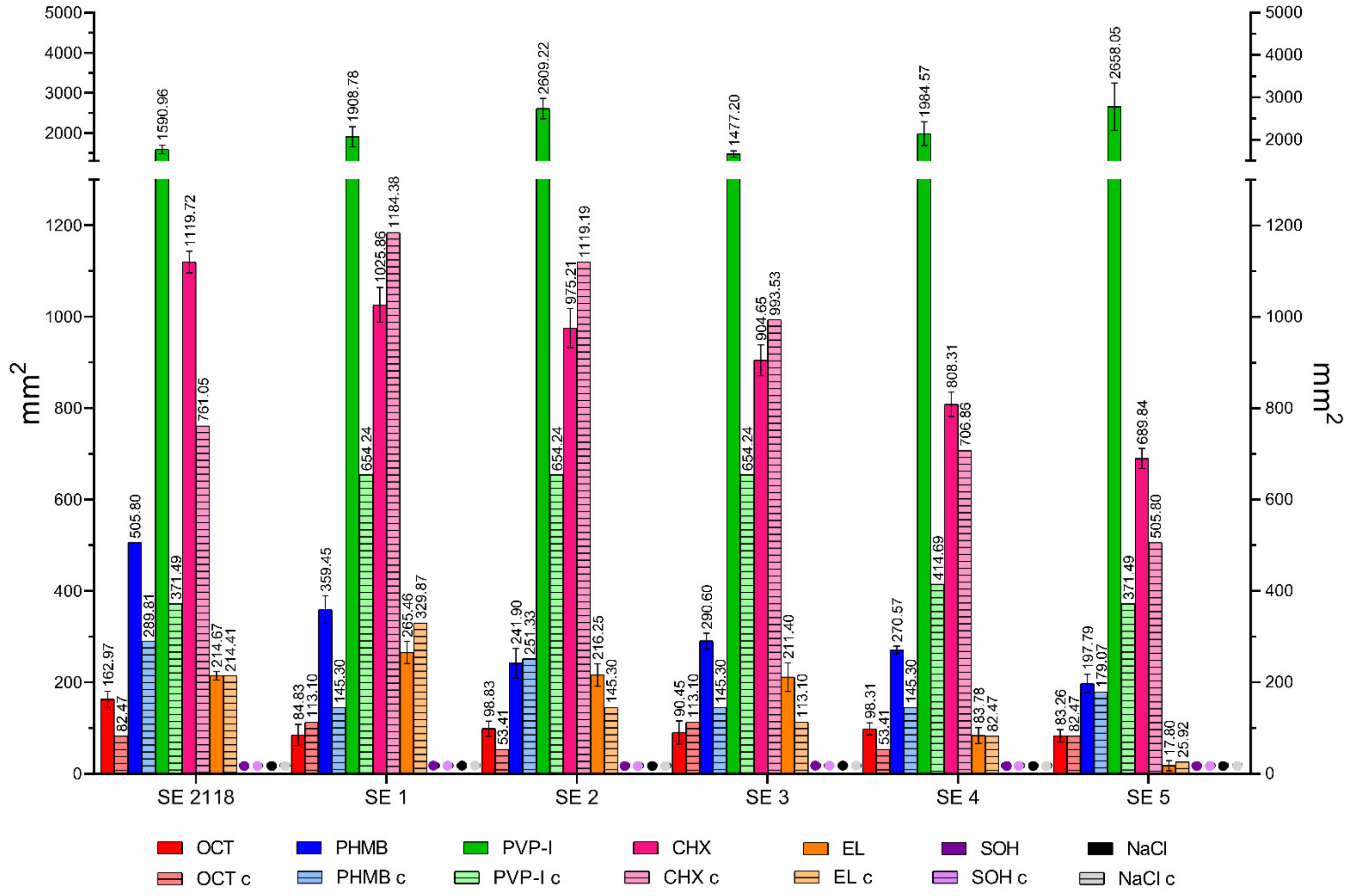

Figure S2. **Areas of growth inhibition zones for *Staphylococcus epidermidis* strains**. Coloured bars indicate average growth inhibition zones areas caused by bacterial cellulose (BC) dressings chemisorbed with tested compounds (OCT – octenidine dihydrochloride, PHMB – polyhexanide, PVP-I – povidone iodine, CHX – chlorhexidine, EL – ethacridine lactate, SOH – super-oxidized hypochlorites solution, NaCl – sodium chloride as a negative control); striped bars indicate areas of growth inhibition zones caused by blotting paper soaked with tested compounds (OCT c – control of OCT activity, PHMB c – control of PHMB activity, PVP-I c – control of PVP-I activity, CHX c – control of CHX activity, EL c – control of EL activity, SOH c – control of SOH activity, NaCl c – control of NaCl activity). Dots point ineffective BC/blotting paper dressings. Growth inhibition zones areas exclude BC/blotting paper dressings surface areas.

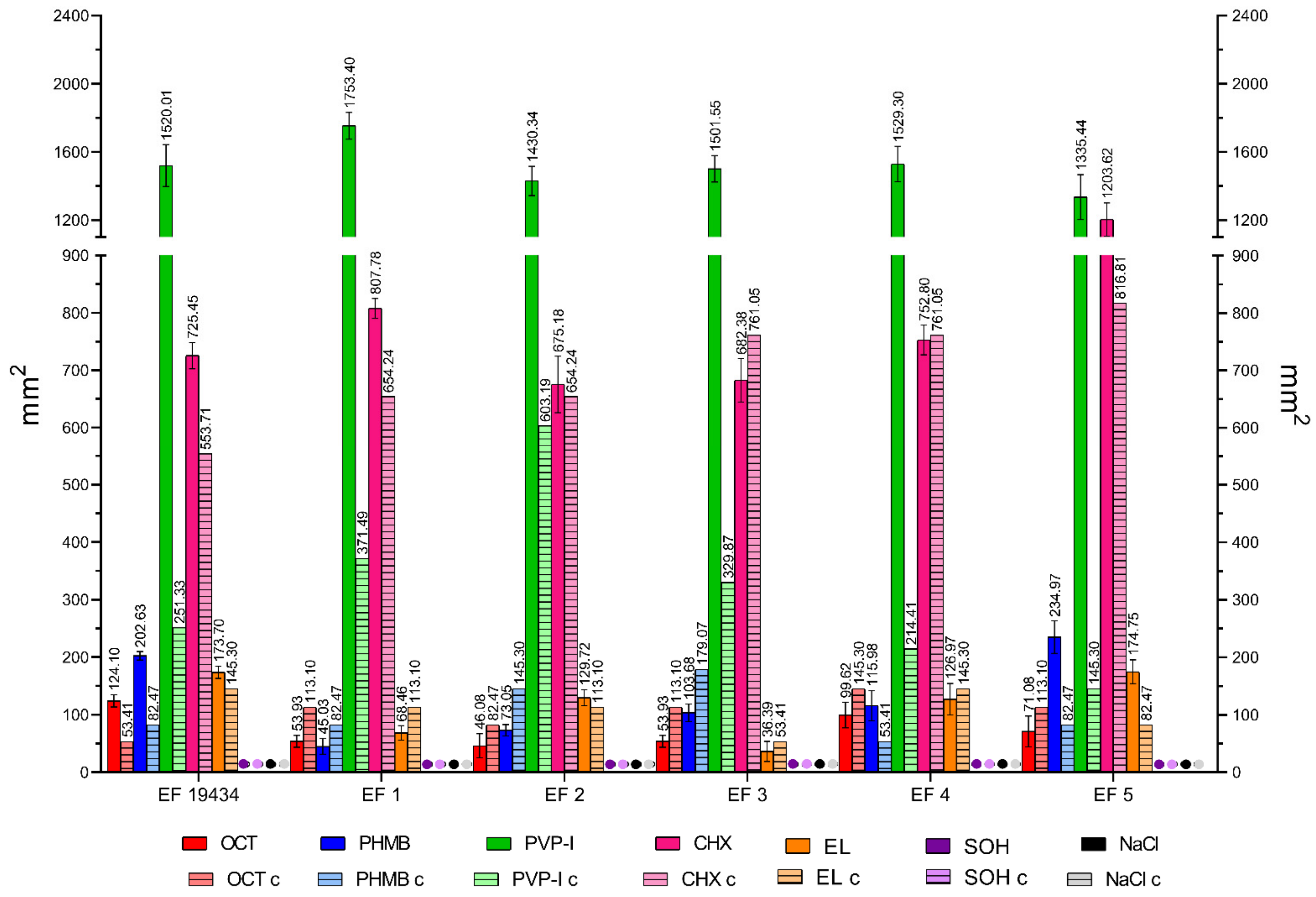

Figure S3. **Areas of growth inhibition zones for *Enterococcus faecium* strains**. Coloured bars indicate average growth inhibition zones areas caused by bacterial cellulose (BC) dressings chemisorbed with tested compounds (OCT – octenidine dihydrochloride, PHMB – polyhexanide, PVP-I – povidone iodine, CHX – chlorhexidine, EL – ethacridine lactate, SOH – super-oxidized hypochlorites solution, NaCl – sodium chloride as a negative control); striped bars indicate areas of growth inhibition zones caused by blotting paper soaked with tested compounds (OCT c – control of OCT activity, PHMB c – control of PHMB activity, PVP-I c – control of PVP-I activity, CHX c – control of CHX activity, EL c – control of EL activity, SOH c – control of SOH activity, NaCl c – control of NaCl activity). Dots point ineffective BC/blotting paper dressings. Growth inhibition zones areas exclude BC/blotting paper dressings surface areas.

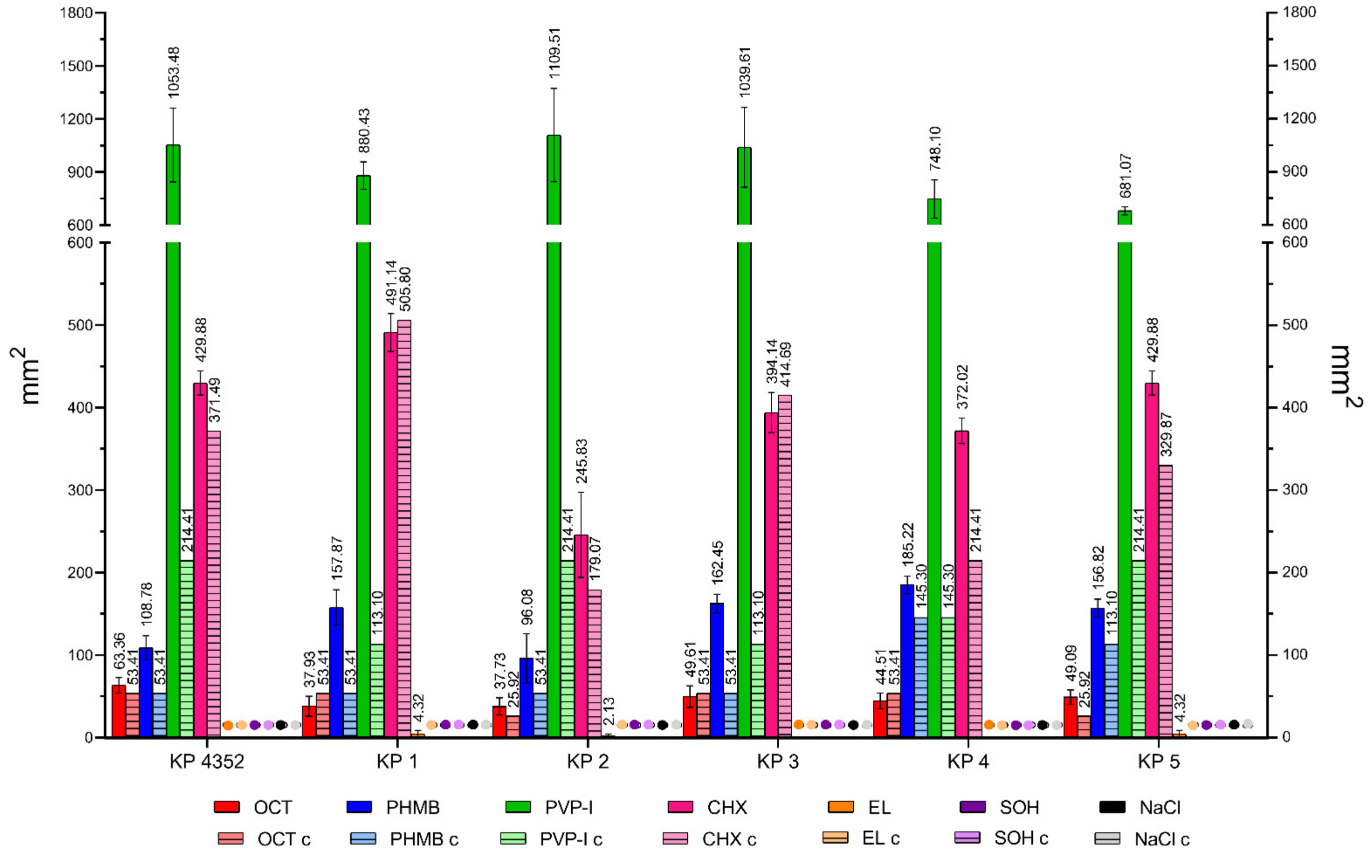

Figure S4. **Areas of growth inhibition zones for *Klebsiella pneumoniae* strains.** Coloured bars indicate average growth inhibition zones areas caused by bacterial cellulose (BC) dressings chemisorbed with tested compounds (OCT – octenidine dihydrochloride, PHMB – polyhexanide, PVP-I – povidone iodine, CHX – chlorhexidine, EL – ethacridine lactate, SOH – super-oxidized hypochlorites solution, NaCl – sodium chloride as a negative control); striped bars indicate areas of growth inhibition zones caused by blotting paper soaked with tested compounds (OCT c – control of OCT activity, PHMB c – control of PHMB activity, PVP-I c – control of PVP-I activity, CHX c – control of CHX activity, EL c – control of EL activity, SOH c – control of SOH activity, NaCl c – control of NaCl activity). Dots point ineffective BC/blotting paper dressings. Growth inhibition zones areas exclude BC/blotting paper dressings surface areas.

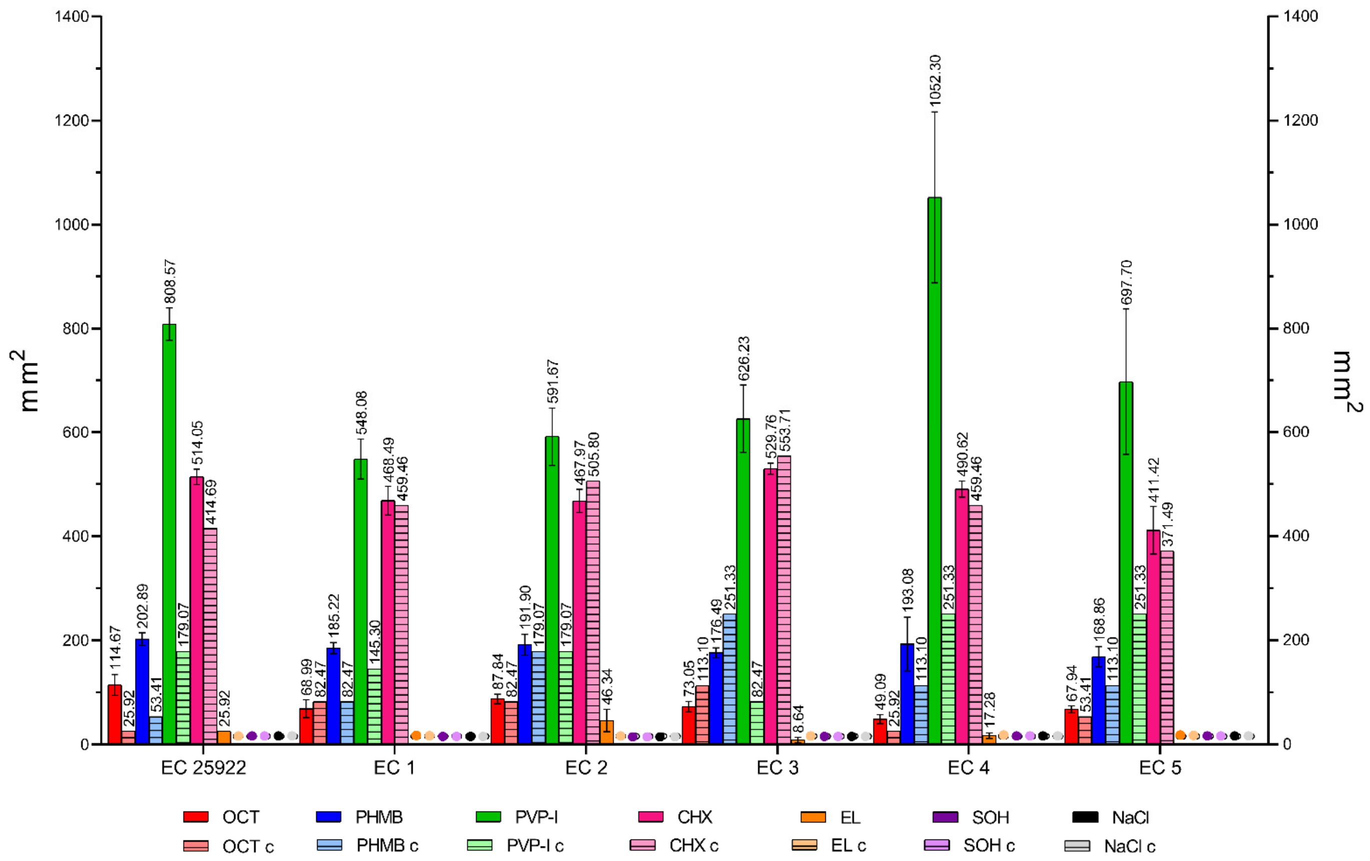

Figure S5. Areas of growth inhibition zones for *Escherichia coli* strains. Coloured bars indicate average growth inhibition zones areas caused by bacterial cellulose (BC) dressings chemisorbed with tested compounds (OCT – octenidine dihydrochloride, PHMB – polyhexanide, PVP-I – povidone iodine, CHX – chlorhexidine, EL – ethacridine lactate, SOH – super-oxidized hypochlorites solution, NaCl – sodium chloride as a negative control); striped bars indicate areas of growth inhibition zones caused by blotting paper soaked with tested compounds (OCT c – control of OCT activity, PHMB c – control of PHMB activity, PVP-I c – control of PVP-I activity, CHX c – control of CHX activity, EL c – control of EL activity, SOH c – control of SOH activity, NaCl c – control of NaCl activity). Dots point ineffective BC/blotting paper dressings. Growth inhibition zones areas exclude BC/blotting paper dressings surface areas.

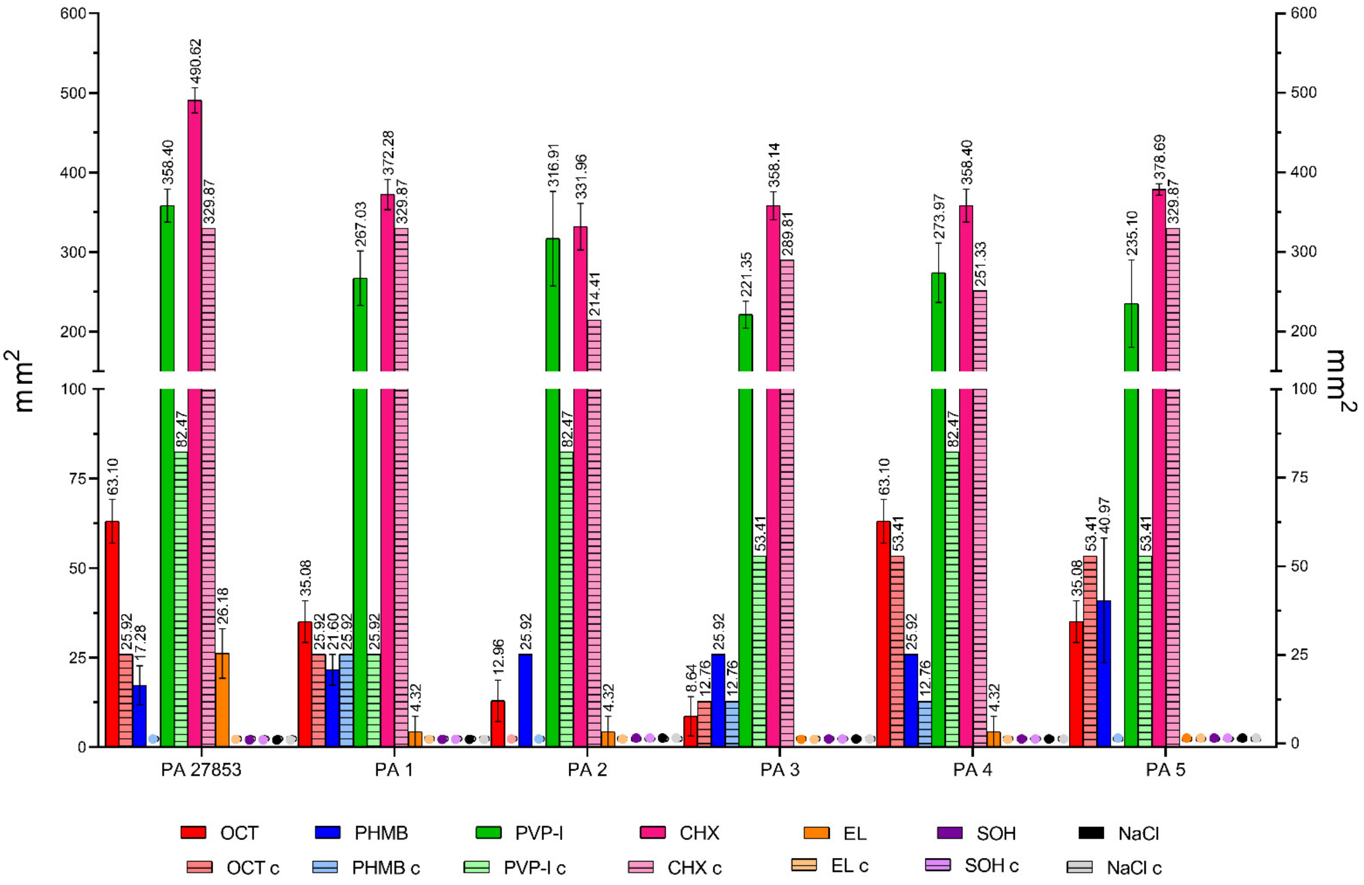

Figure S6. Areas of growth inhibition zones for *Pseudomonas aeruginosa* strains. Coloured bars indicate average growth inhibition zones areas caused by bacterial cellulose (BC) dressings chemisorbed with tested compounds (OCT – octenidine dihydrochloride, PHMB – polyhexanide, PVP-I – povidone iodine, CHX – chlorhexidine, EL – ethacridine lactate, SOH – super-oxidized hypochlorites solution, NaCl – sodium chloride as a negative control); striped bars indicate areas of growth inhibition zones caused by blotting paper soaked with tested compounds (OCT c – control of OCT activity, PHMB c – control of PHMB activity, PVP-I c – control of PVP-I activity, CHX c – control of CHX activity, EL c – control of EL activity, SOH c – control of SOH activity, NaCl c – control of NaCl activity). Dots point ineffective BC/blotting paper dressings. Growth inhibition zones areas exclude BC/blotting paper dressings surface areas.

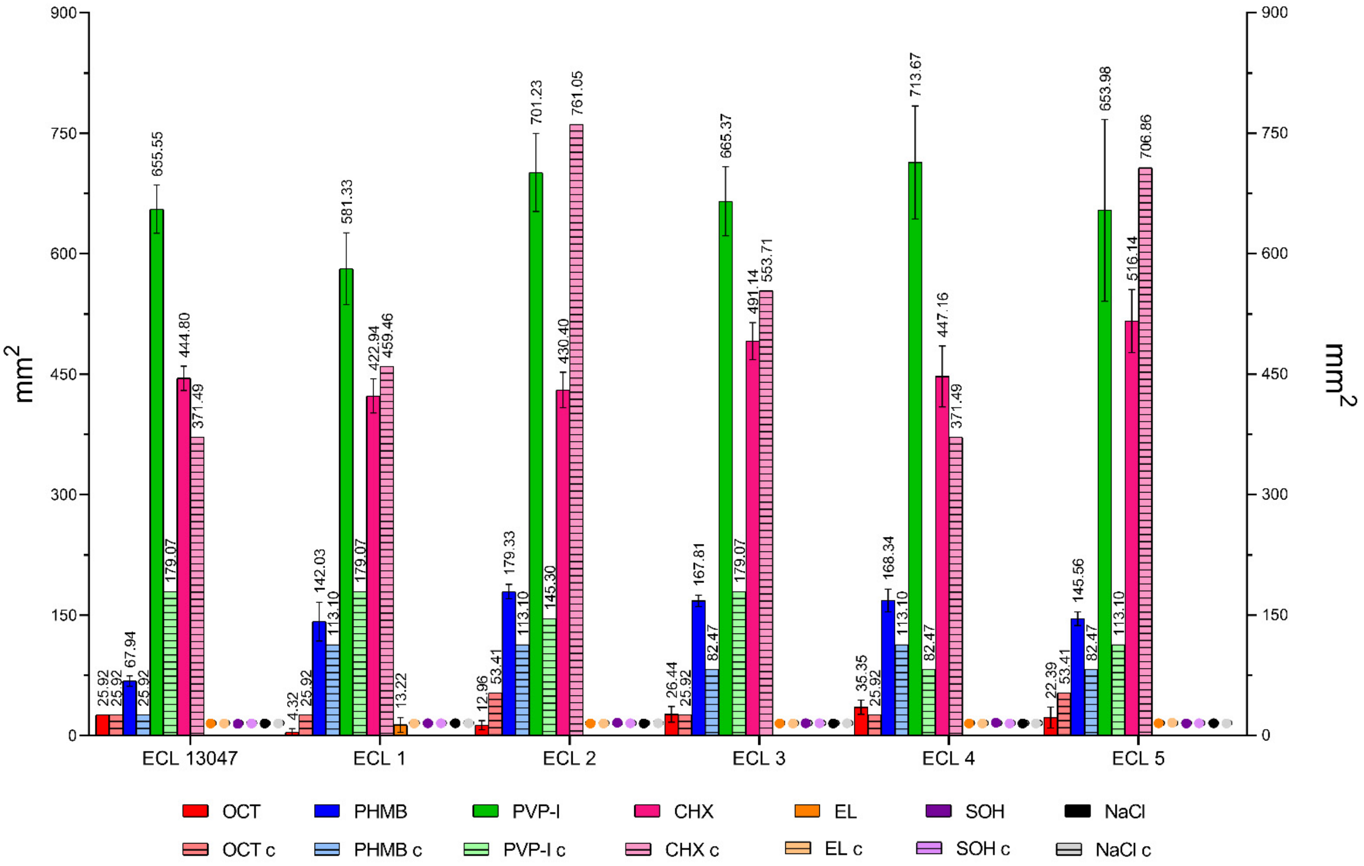

Figure S7. Areas of growth inhibition zones for *Enterobacter cloacae* strains. Coloured bars indicate average growth inhibition zones areas caused by bacterial cellulose (BC) dressings chemisorbed with tested compounds (OCT – octenidine dihydrochloride, PHMB – polyhexanide, PVP-I – povidone iodine, CHX – chlorhexidine, EL – ethacridine lactate, SOH – super-oxidized hypochlorites solution, NaCl – sodium chloride as a negative control); striped bars indicate areas of growth inhibition zones caused by blotting paper soaked with tested compounds (OCT c – control of OCT activity, PHMB c – control of PHMB activity, PVP-I c – control of PVP-I activity, CHX c – control of CHX activity, EL c – control of EL activity, SOH c – control of SOH activity, NaCl c – control of NaCl activity). Dots point ineffective BC/blotting paper dressings. Growth inhibition zones areas exclude BC/blotting paper dressings surface areas.

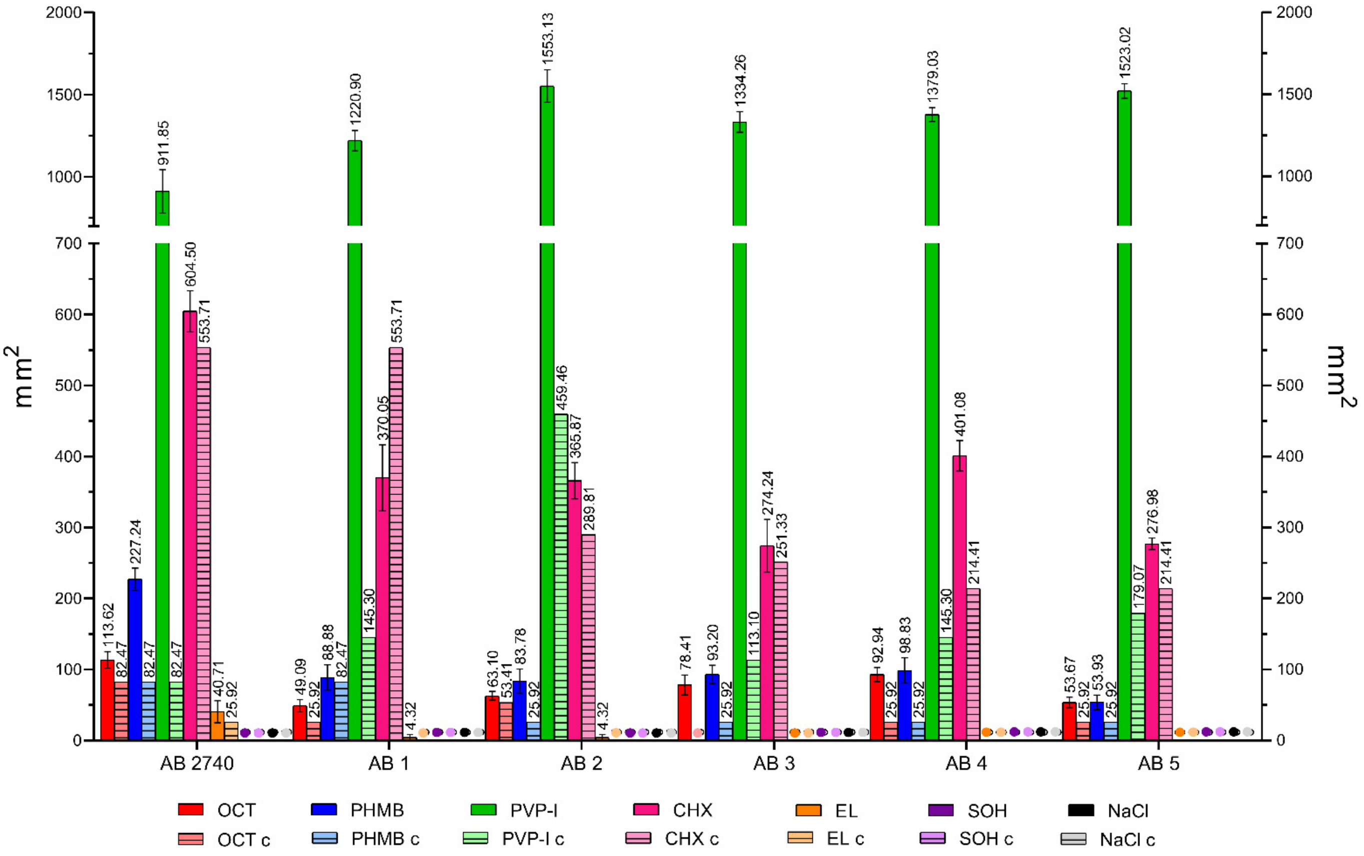

Figure S8. Areas of growth inhibition zones for *Acinetobacter baumannii* strains. Coloured bars indicate average growth inhibition zones areas caused by bacterial cellulose (BC) dressings chemisorbed with tested compounds (OCT – octenidine dihydrochloride, PHMB – polyhexanide, PVP-I – povidone iodine, CHX – chlorhexidine, EL – ethacridine lactate, SOH – super-oxidized hypochlorites solution, NaCl – sodium chloride as a negative control); striped bars indicate areas of growth inhibition zones caused by blotting paper soaked with tested compounds (OCT c – control of OCT activity, PHMB c – control of PHMB activity, PVP-I c – control of PVP-I activity, CHX c – control of CHX activity, EL c – control of EL activity, SOH c – control of SOH activity, NaCl c – control of NaCl activity). Dots point ineffective BC/blotting paper dressings. Growth inhibition zones areas exclude BC/blotting paper dressings surface areas.

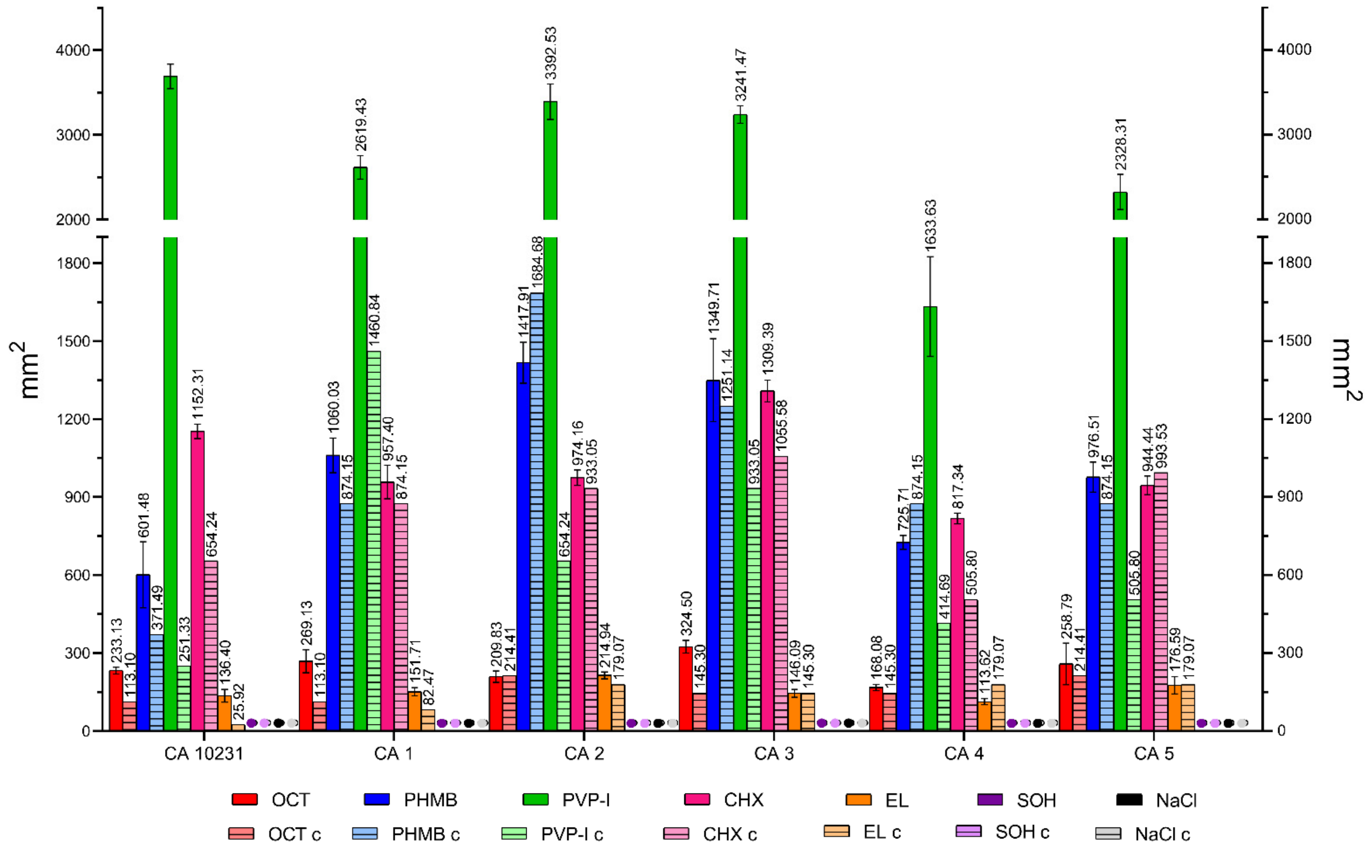

Figure S9. Areas of growth inhibition zones for *Candida albicans* strains. Coloured bars indicate average growth inhibition zones areas caused by bacterial cellulose (BC) dressings chemisorbed with tested compounds (OCT – octenidine dihydrochloride, PHMB – polyhexanide, PVP-I – povidone iodine, CHX – chlorhexidine, EL – ethacridine lactate, SOH – super-oxidized hypochlorites solution, NaCl – sodium chloride as a negative control); striped bars indicate areas of growth inhibition zones caused by blotting paper soaked with tested compounds (OCT c – control of OCT activity, PHMB c – control of PHMB activity, PVP-I c – control of PVP-I activity, CHX c – control of CHX activity, EL c – control of EL activity, SOH c – control of SOH activity, NaCl c – control of NaCl activity). Dots point ineffective BC/blotting paper dressings. Growth inhibition zones areas exclude BC/blotting paper dressings surface areas.

Table S2. Areas of growth inhibition zones around BC dressings (BC) chemisorbed with tested antimicrobial compounds. OCT – octenidine dihydrochloride, PHMB – polyhexanide, PVP-I – povidone iodine, CHX – chlorhexidine, EL – ethacridine lactate, SOH – super-oxidized hypochlorites solution. As a compound's activity control, soaked blotting paper discs were used. NaCl was used as a negative control. Values presented in the Table are reduced by areas of BC dressings or blotting paper discs. Rep – repetitions; con – blotting paper control; AA – arithmetic average, SD – standard deviation, SEM – standard error of mean. Tested strains: SA – *Staphylococcus aureus*, SE – *Staphylococcus epidermidis*, EF – *Enterococcus faecium*, KP – *Klebsiella pneumoniae*, EC – *Escherichia coli*, PA – *Pseudomonas aeruginosa*, ECL – *Enterobacter cloacae*, AB – *Acinetobacter baumannii*, CA – *Candida albicans*.

	OCT											PHMB										
	BC dressings						AA	SD	SEM		con		BC dressings						AA	SD	SEM		con
rep	1	2	3	4	5	6						rep	1	2	3	4	5	6					
SAW33591	113,10	82,47	145,30	145,30	145,30	113,10	124,09	25,78	10,53		82,47	SAW33591	214,41	371,49	414,69	414,69	251,33	371,49	339,68	85,76	35,01		113,10
SA 1	145,30	289,81	214,41	113,10	113,10	82,47	159,70	78,02	31,85		113,10	SA 1	179,07	145,30	145,30	145,30	179,07	251,33	174,23	41,24	16,83		145,30
SA 2	113,10	505,80	179,07	214,41	113,10	113,10	206,43	152,69	62,33		82,47	SA 2	179,07	179,07	179,07	179,07	179,07	179,07	179,07	0,00	0,00		82,47
SA 3	25,92	251,33	113,10	82,47	251,33	145,30	144,91	91,35	37,29		53,41	SA 3	179,07	179,07	113,10	145,30	179,07	113,10	151,45	32,46	13,25		179,07
SA 4	82,47	113,10	53,41	82,47	53,41	53,41	73,04	24,24	9,90		82,47	SA 4	179,07	113,10	179,07	179,07	145,30	145,30	156,82	27,06	11,05		53,41
SA 5	113,10	113,10	145,30	113,10	113,10	113,10	118,46	13,15	5,37		53,41	SA 5	113,10	145,30	145,30	145,30	113,10	145,30	134,56	16,63	6,79		82,47
						AA	137,77			AA	77,89							AA	189,30			AA	109,30
						SD	44,71			SD	22,37							SD	75,41			SD	46,35
						SEM	18,25			SEM	54,79							SEM	30,78			SEM	113,54
SE 2118	145,30	251,33	145,30	145,30	145,30	145,30	162,97	43,29	17,67		82,47	SE 2118	505,80	505,80	505,80	505,80	505,80	505,80	505,80	0,00	0,00		289,81

[illegible]

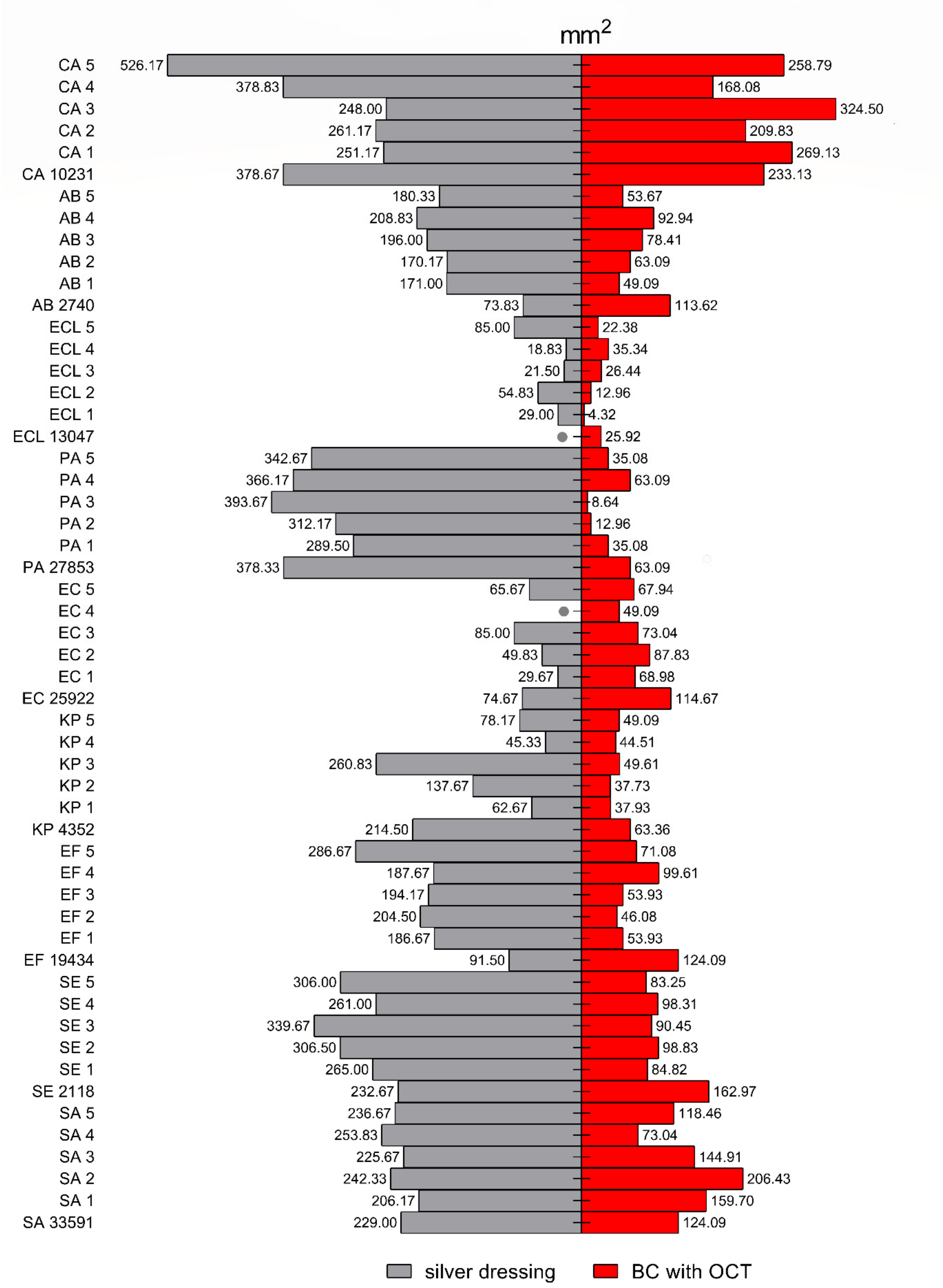

Figure S10. Graphic demonstration of average growth inhibition zones areas [mm²] caused by silver dressing comparison to BC dressing chemisorbed with octenidine (BC with OCT). Silver dressing – Aquacel® Ag (ConvaTec, Berkshire, England). Tested strains: SA – *Staphylococcus aureus*, SE – *Staphylococcus epidermidis*, EF – *Enterococcus faecium*, KP – *Klebsiella pneumoniae*, EC – *Escherichia coli*, PA – *Pseudomonas aeruginosa*, ECL – *Enterobacter cloacae*, AB – *Acinetobacter baumannii*, CA – *Candida albicans*. Dots point ineffective BC/silver dressings. Demonstrated growth inhibition zones areas [mm²] exclude BC/silver dressings surface areas.

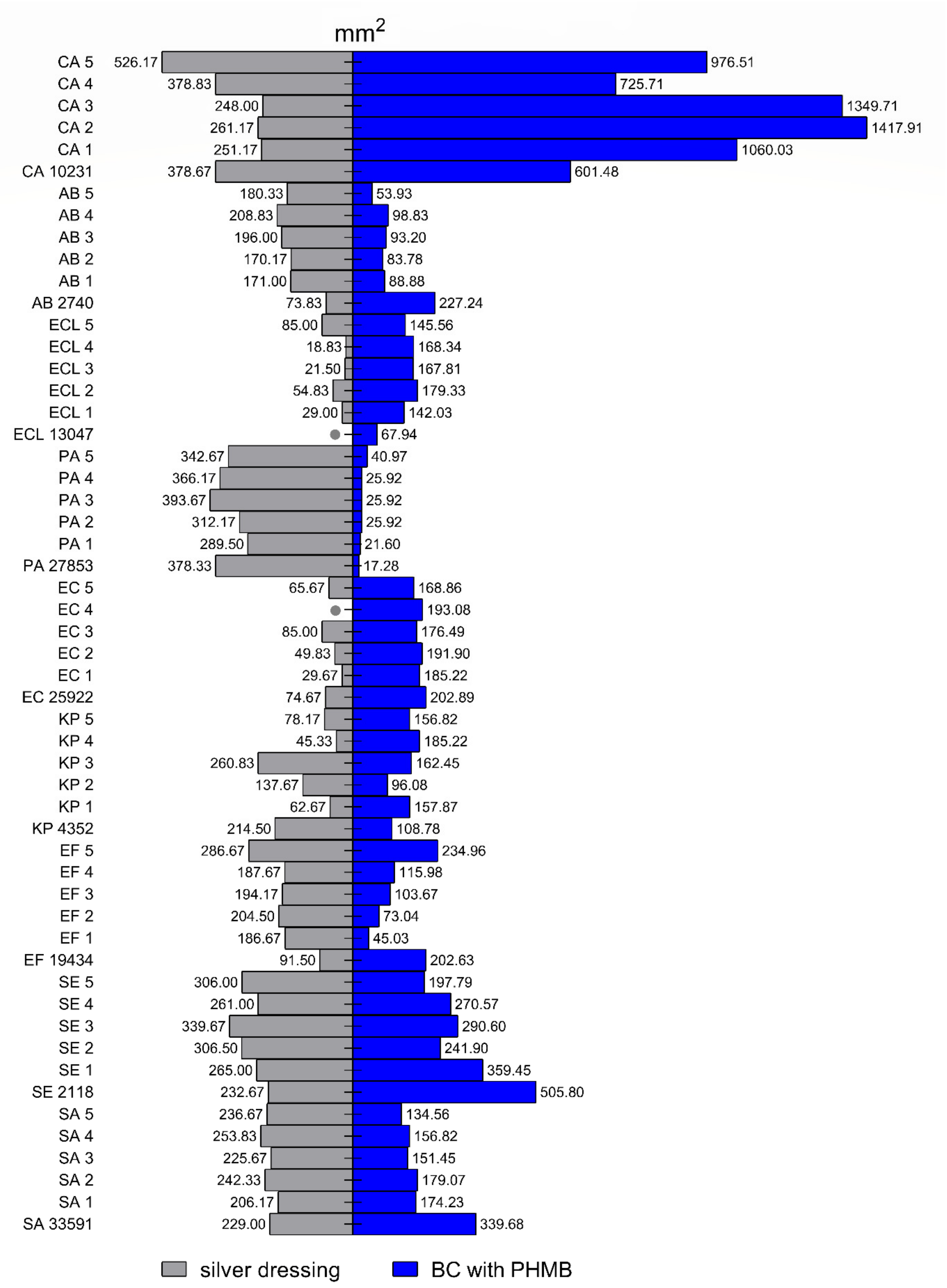

Figure S11. Graphic demonstration of average growth inhibition zones areas caused by silver dressing comparison to BC dressing chemisorbed with polyhexanide (BC with PHMB). Silver dressing – Aquacel® Ag (ConvaTec, Berkshire, England). Tested strains: SA – *Staphylococcus aureus*, SE – *Staphylococcus epidermidis*, EF – *Enterococcus faecium*, KP – *Klebsiella pneumoniae*, EC – Escherichia coli, PA – *Pseudomonas aeruginosa*, ECL – *Enterobacter cloacae*, AB – *Acinetobacter baumannii*, CA – *Candida albicans*. Dots point ineffective BC/silver dressings. Demonstrated growth inhibition zones areas [mm^2] exclude BC/silver dressings surface areas.

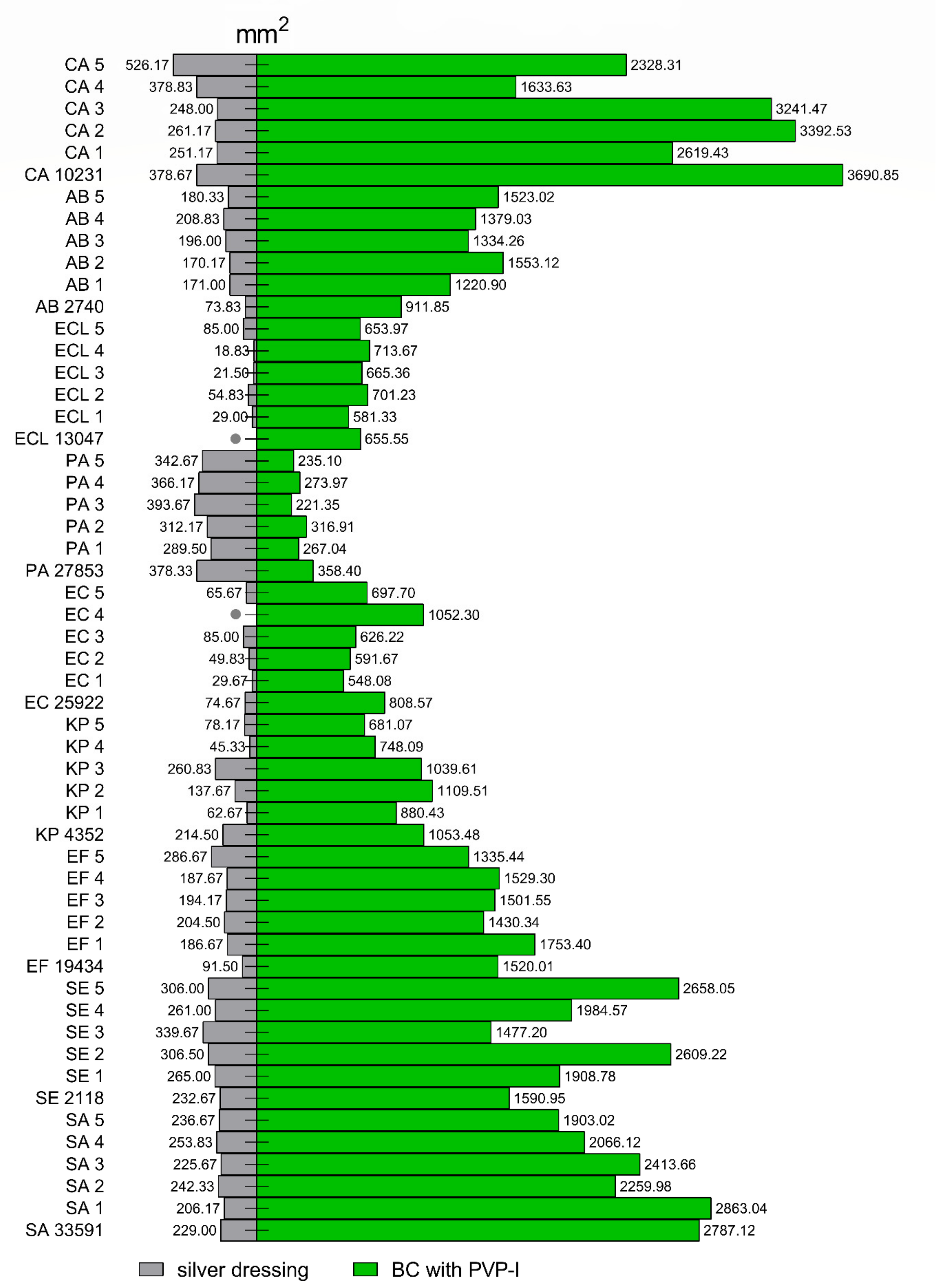

Figure S12. Graphic demonstration of average growth inhibition zones areas caused by silver dressing comparison to BC dressing chemisorbed with povidone iodine (BC with PVP-I). Silver dressing – Aquacel® Ag (ConvaTec, Berkshire, England). Tested strains: SA – *Staphylococcus aureus*, SE – *Staphylococcus epidermidis*, EF – *Enterococcus faecium*, KP – *Klebsiella pneumoniae*, EC – *Escherichia coli*, PA – *Pseudomonas aeruginosa*, ECL – *Enterobacter cloacae*, AB – *Acinetobacter baumannii*, CA – *Candida albicans*. Dots point ineffective BC/silver dressings. Demonstrated growth inhibition zones areas [mm²] exclude BC/silver dressings surface areas.

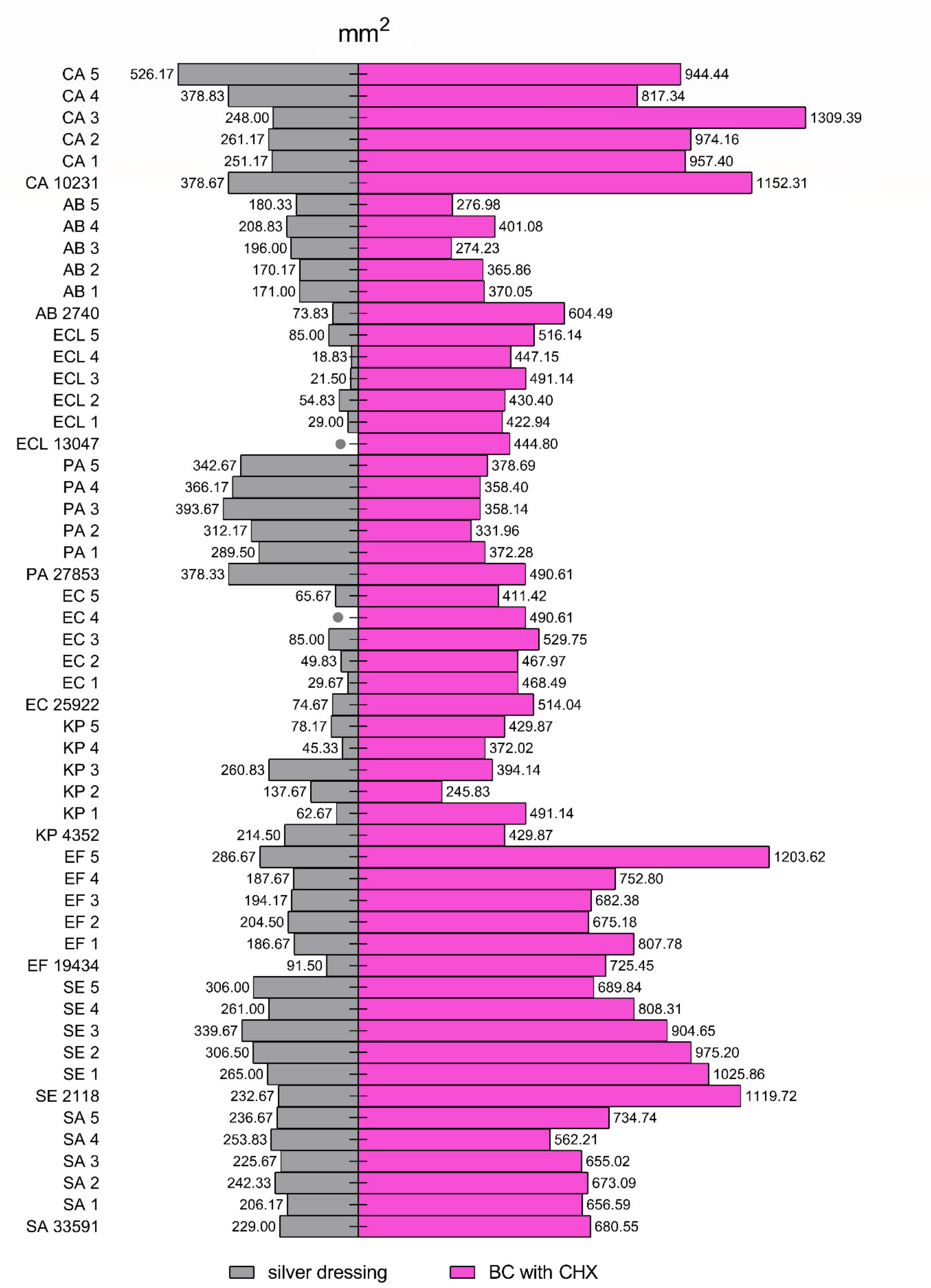

Figure S13. Graphic demonstration of average growth inhibition zones areas caused by silver dressing comparison to BC dressing chemisorbed with chlorhexidine (BC with CHX). Silver dressing – Aquacel® Ag (ConvaTec, Berkshire, England). Tested strains: SA – *Staphylococcus aureus*, SE – *Staphylococcus epidermidis*, EF – *Enterococcus faecium*, KP – *Klebsiella pneumoniae*, EC – *Escherichia coli*, PA – *Pseudomonas aeruginosa*, ECL – *Enterobacter cloacae*, AB – *Acinetobacter baumannii*, CA – *Candida albicans*. Dots point ineffective BC/silver dressings. Demonstrated growth inhibition zones areas [mm^2] exclude BC/silver dressings surface areas.

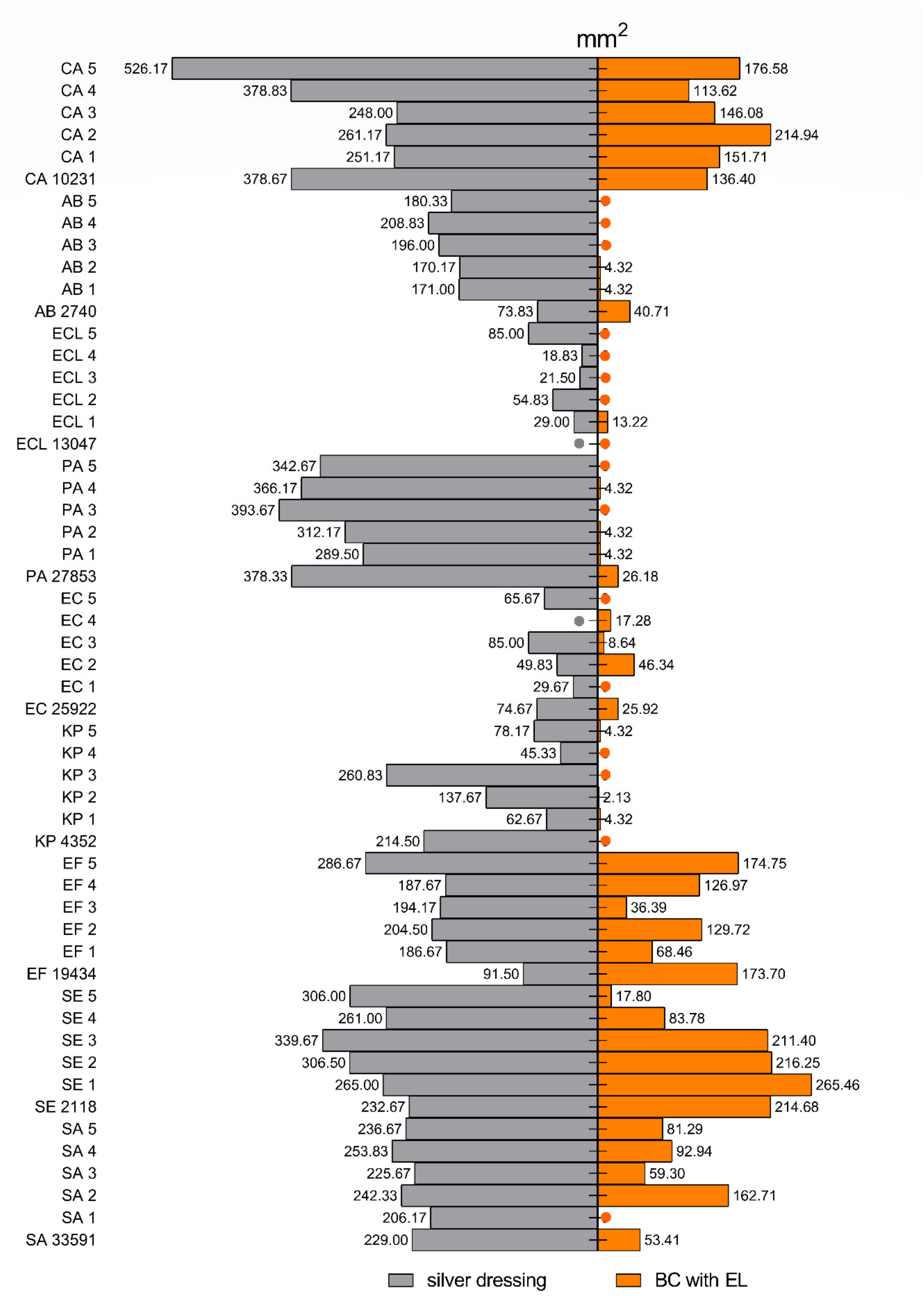

Figure S14. Graphic demonstration of average growth inhibition zones areas caused by silver dressing comparison to BC dressing chemisorbed with ethacridine lactate (BC with EL). Silver dressing – Aquacel® Ag (ConvaTec, Berkshire, England). Tested strains: SA – *Staphylococcus aureus*, SE – *Staphylococcus epidermidis*, EF – *Enterococcus faecium*, KP – *Klebsiella pneumoniae*, EC – *Escherichia coli*, PA – *Pseudomonas aeruginosa*, ECL – *Enterobacter cloacae*, AB – *Acinetobacter baumannii*, CA – *Candida albicans*. Dots point ineffective BC/silver dressings. Demonstrated growth inhibition zones areas [mm^2] exclude BC/silver dressings surface areas.

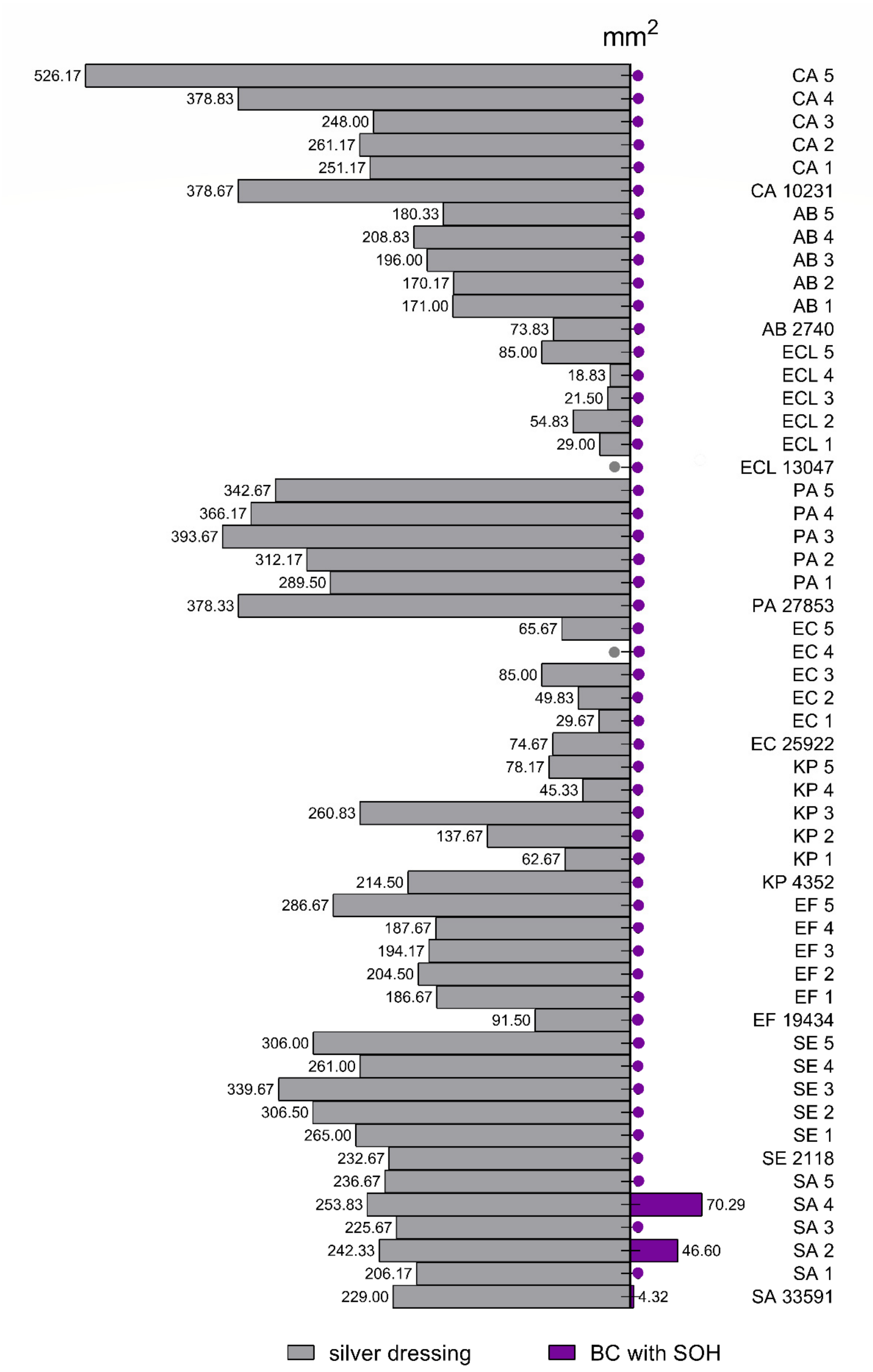

Figure S15. Graphic demonstration of average growth inhibition zones areas caused by silver dressing comparison to BC dressing chemisorbed with super-oxidized hypochlorous solution (BC with SOH). Silver dressing – Aquacel® Ag (ConvaTec, Berkshire, England). Tested strains: SA – *Staphylococcus aureus*, SE – *Staphylococcus epidermidis*, EF – *Enterococcus faecium*, KP – *Klebsiella pneumoniae*, EC – *Escherichia coli*, PA – *Pseudomonas aeruginosa*, ECL – *Enterobacter cloacae*, AB – *Acinetobacter baumannii*, CA – *Candida albicans*. Dots point ineffective BC/silver dressings. Demonstrated growth inhibition zones areas [mm^2] exclude BC/silver dressings surface areas.

Table S3. Average growth inhibition zones [mm^2] obtained in modified disc-diffusion method with silver dressing use. For every strain there were 6 repetitions. Values presented in the Table are reduced by areas of silver dressings. Silver dressing – Aquacel® Ag (ConvaTec, Berkshire, England). AA – arithmetic average, SD – standard deviation, SEM – standard error of mean, AAs – arithmetic average for whole species, SDs – standard deviation for whole species, SEMs – standard error of mean for whole species. Tested species: SA – *Staphylococcus aureus*, SE – *Staphylococcus epidermidis*, EF – *Enterococcus faecium*, KP – *Klebsiella pneumoniae*, EC – *Escherichia coli*, PA – *Pseudomonas aeruginosa*, ECL – *Enterobacter cloacae*, AB – *Acinetobacter baumannii*, CA – *Candida albicans*.

strain	growth inhibition zones [mm^2]						AA	SD	SEM	AAs	SDs	SEMs
**SA 33591**	274	224	245	245	203	183	**229,00**	**32,72**	**13,36**	**232,28**	**33,68**	**5,61**
**SA 1**	165	238	245	189	190	210	**206,17**	**30,94**	**12,63**			
**SA 2**	230	250	287	217	204	266	**242,33**	**31,23**	**12,75**			
**SA 3**	244	266	230	179	190	245	**225,67**	**34,07**	**13,91**			
**SA 4**	304	287	273	259	190	210	**253,83**	**44,74**	**18,26**			
**SA 5**	259	245	224	230	224	238	**236,67**	**13,68**	**5,58**			
**SE 2118**	231	252	210	204	224	275	**232,67**	**26,76**	**10,92**	**285,14**	**57,05**	**9,51**
**SE 1**	170	320	351	259	259	231	**265,00**	**64,30**	**26,25**			
**SE 2**	280	383	324	301	333	218	**306,50**	**55,54**	**22,68**			
**SE 3**	380	280	302	347	311	418	**339,67**	**52,19**	**21,31**			
**SE 4**	304	259	245	238	245	275	**261,00**	**24,86**	**10,15**			
**SE 5**	311	342	334	274	224	351	**306,00**	**48,74**	**19,90**			
**EF 19434**	76	87	90	93	107	96	**91,50**	**10,25**	**4,19**	**191,86**	**60,85**	**10,14**
**EF 1**	204	198	165	165	198	190	**186,67**	**17,36**	**7,09**			
**EF 2**	198	184	198	217	178	252	**204,50**	**26,91**	**10,98**			
**EF 3**	204	168	174	230	211	178	**194,17**	**24,56**	**10,03**			
**EF 4**	192	198	167	179	198	192	**187,67**	**12,27**	**5,01**			
**EF 5**	259	258	324	320	293	266	**286,67**	**30,20**	**12,33**			
**KP 4352**	198	198	204	252	211	224	**214,50**	**20,80**	**8,49**	**133,19**	**83,76**	**13,96**
**KP 1**	88	60	41	56	60	71	**62,67**	**15,74**	**6,43**			
**KP 2**	155	128	124	128	150	141	**137,67**	**12,94**	**5,28**			
**KP 3**	204	273	272	238	266	312	**260,83**	**36,52**	**14,91**			
**KP 4**	14	72	46	44	64	32	**45,33**	**21,08**	**8,60**			
**KP 5**	76	76	71	74	73	99	**78,17**	**10,38**	**4,24**			
**EC 25922**	58	29	29	114	90	128	**74,67**	**42,60**	**17,39**	**50,81**	**39,70**	**6,62**
**EC 1**	30	0	28	29	46	45	**29,67**	**16,65**	**6,80**			
**EC 2**	76	59	76	44	0	44	**49,83**	**28,30**	**11,55**			
**EC 3**	160	76	58	57	43	116	**85,00**	**44,60**	**18,21**			
**EC 4**	0	0	0	0	0	0	**0,00**	**0,00**	**0,00**			
**EC 5**	58	58	58	70	60	90	**65,67**	**12,80**	**5,23**			
**PA 27853**	304	370	418	436	418	324	**378,33**	**54,81**	**22,38**	**347,08**	**61,38**	**10,23**
**PA 1**	238	324	324	259	342	250	**289,50**	**45,34**	**18,51**			
**PA 2**	333	333	297	324	294	292	**312,17**	**19,87**	**8,11**			
**PA 3**	272	456	442	404	418	370	**393,67**	**66,76**	**27,25**			
**PA 4**	370	370	365	380	370	342	**366,17**	**12,81**	**5,23**			
**PA 5**	394	434	326	259	238	405	**342,67**	**81,36**	**33,21**			
**ECL 13047**	0	0	0	0	0	0	**0,00**	**0,00**	**0,00**	**34,86**	**38,84**	**6,47**
**ECL 1**	58	58	0	0	0	58	**29,00**	**31,77**	**12,97**			
**ECL 2**	124	58	60	0	43	44	**54,83**	**40,21**	**16,42**			
**ECL 3**	0	73	0	0	56	0	**21,50**	**33,74**	**13,77**			
**ECL 4**	0	0	0	0	42	71	**18,83**	**30,58**	**12,49**			
**ECL 5**	48	87	93	99	93	90	**85,00**	**18,56**	**7,58**			
**AB 2740**	90	65	78	93	58	59	**73,83**	**15,46**	**6,31**	**166,69**	**60,41**	**10,07**
**AB 1**	184	223	204	160	142	113	**171,00**	**40,71**	**16,62**			
**AB 2**	198	165	196	165	132	165	**170,17**	**24,41**	**9,96**			
**AB 3**	198	198	259	152	204	165	**196,00**	**37,25**	**15,21**			
**AB 4**	210	145	370	170	128	230	**208,83**	**87,80**	**35,84**			
**AB 5**	160	174	189	198	173	188	**180,33**	**13,81**	**5,64**			
**CA 10231**	393	337	418	337	333	454	**378,67**	**50,96**	**20,80**	**340,67**	**105,29**	**17,55**
**CA 1**	245	245	280	204	245	288	**251,17**	**30,09**	**12,28**			
**CA 2**	259	259	259	259	258	273	**261,17**	**5,81**	**2,37**			
**CA 3**	204	274	245	224	267	274	**248,00**	**29,10**	**11,88**			
**CA 4**	380	334	404	405	370	380	**378,83**	**26,11**	**10,66**			
**CA 5**	519	520	546	506	520	546	**526,17**	**16,25**	**6,64**			

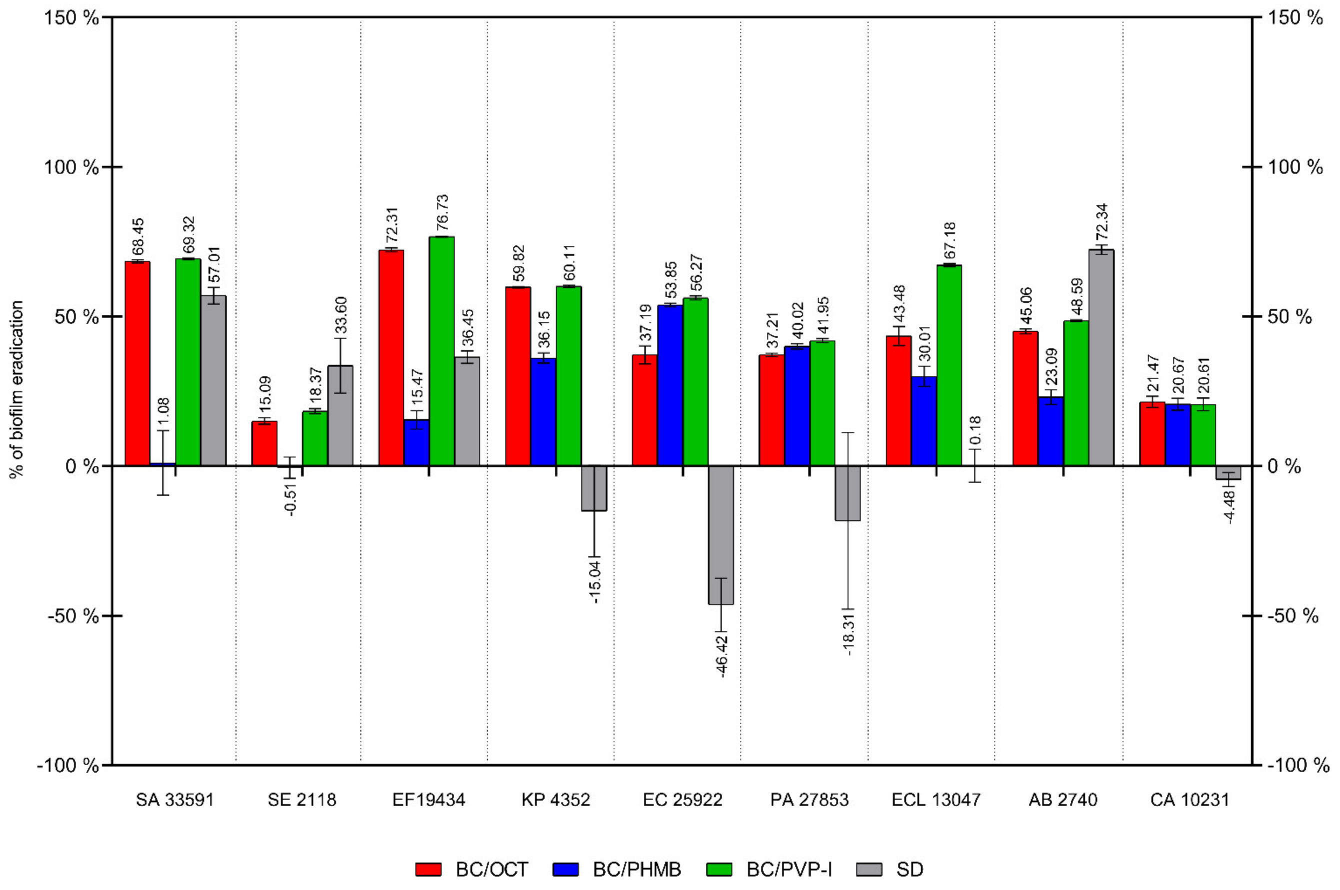

Figure S16. The results of A.D.A.M. test presented as an average percent of metabolically active cells in biofilm eradication in tryptic-soy broth (TSB) culture medium. Tested dressings: BC/OCT – bacterial cellulose with octenidine dihydrochloride, BC/PHMB – bacterial cellulose with polyhexanide, BC/PVP-I – bacterial cellulose with povidone iodine, SD – silver dressing (Aquacel® Ag, ConvaTec, Berkshire, England). Tested strains: SA 33591 – *Staphylococcus aureus* ATCC 33591, SE 2118 – *Staphylococcus epidermidis* PCM 2118, EF 19434 – *Enterococcus faecium* ATCC 19434, KP 4352 – *Klebsiella pneumoniae* ATCC 4352, EC 25922 – *Escherichia coli* ATCC 25922, PA 27853 – *Pseudomonas aeruginosa* ATCC 27853, ECL 13047 – *Enterobacter cloacae* ATCC 13047, AB 2740 – *Acinetobacter baumannii* PCM 2740, CA 10231 – *Candida albicans* ATCC 10231.

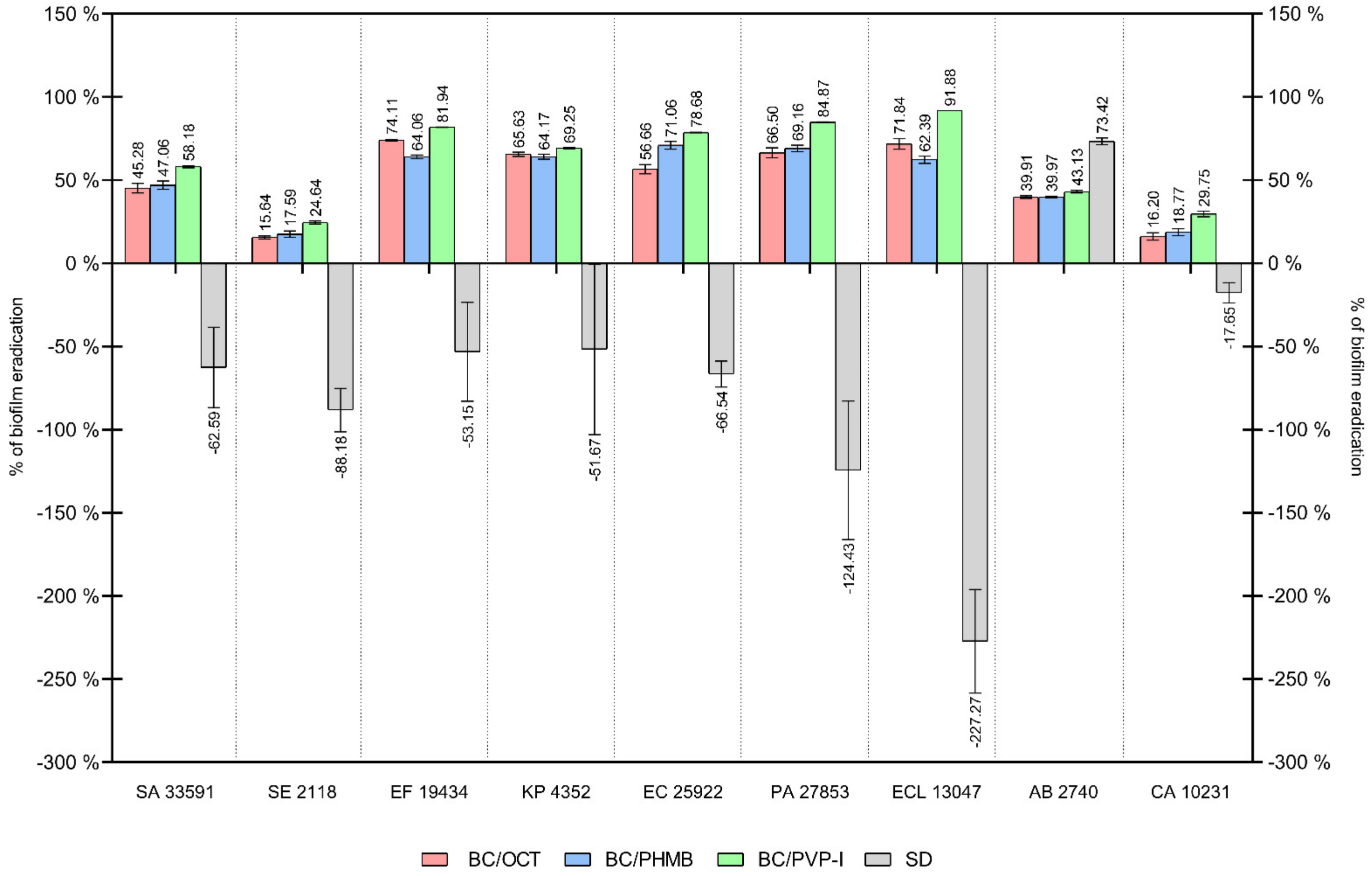

Figure S17. The results of A.D.A.M. test presented as an average percent of metabolically active cells in biofilm eradication in artificial exudate (AE) culture medium. Tested dressings: BC/OCT – bacterial cellulose with octenidine dihydrochloride, BC/PHMB – bacterial cellulose with polyhexanide, BC/PVP-I – bacterial cellulose with povidone iodine, SD – silver dressing (Aquacel® Ag, ConvaTec, Berkshire, England). Tested strains: SA 33591 – *Staphylococcus aureus* ATCC 33591, SE 2118 – *Staphylococcus epidermidis* PCM 2118, EF 19434 – *Enterococcus faecium* ATCC 19434, KP 4352 – *Klebsiella pneumoniae* ATCC 4352, EC 25922 – *Escherichia coli* ATCC 25922, PA 27853 – *Pseudomonas aeruginosa* ATCC 27853, ECL 13047 – *Enterobacter cloacae* ATCC 13047, AB 2740 – *Acinetobacter baumannii* PCM 2740, CA 10231 – *Candida albicans* ATCC 10231.

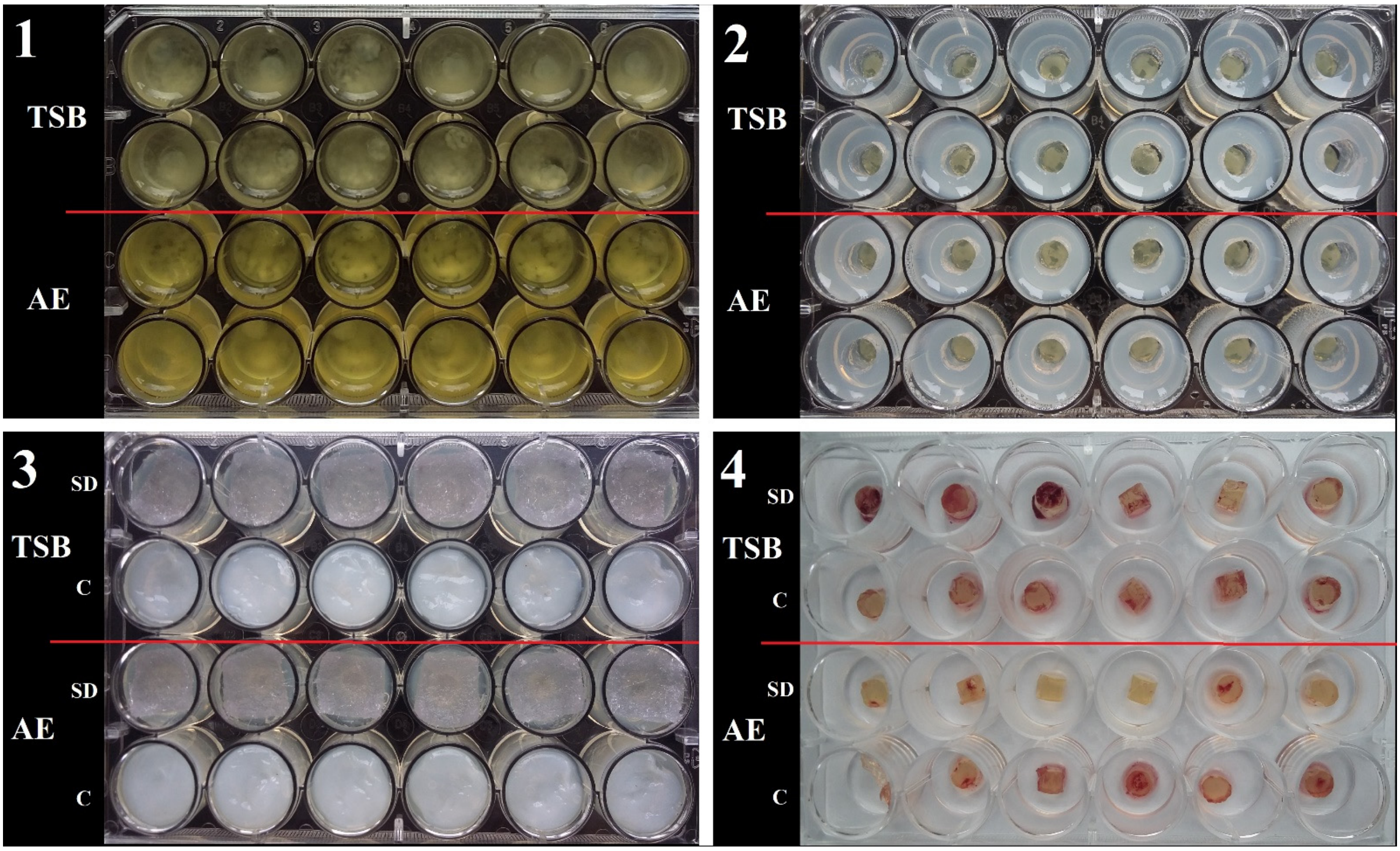

Figure S18. Stages of the modified A.D.A.M. test. 1 – *Staphylococcus aureus* ATCC 33591 biofilm culture on agar discs; 2 – placing agar discs with biofilm into agar tubes and filling the tubes with culture media; 3 – covering the tubes with tested dressings; 4 – staining of biofilm with the 2, 3, 5- triphenyl tetrazolium chloride after 24h incubation with tested dressings. TSB – tryptic soy broth culture medium; AE – artificial exudate; SD - Aquacel® Ag silver dressing (ConvaTec, Berkshire, England); C – negative control: bacterial cellulose chemisorbed with sterile saline.
